# Histone variant macroH2A1 regulates synchronous firing of replication origins in the inactive X chromosome

**DOI:** 10.1093/nar/gkae734

**Published:** 2024-08-27

**Authors:** Maria Arroyo, Corella S Casas-Delucchi, Maruthi K Pabba, Paulina Prorok, Sunil K Pradhan, Cathia Rausch, Anne Lehmkuhl, Andreas Maiser, Marcus Buschbeck, Vincent Pasque, Emily Bernstein, Katja Luck, M Cristina Cardoso

**Affiliations:** Cell Biology and Epigenetics, Department of Biology, Technical University of Darmstadt, 64287 Darmstadt, Germany; Cell Biology and Epigenetics, Department of Biology, Technical University of Darmstadt, 64287 Darmstadt, Germany; Cell Biology and Epigenetics, Department of Biology, Technical University of Darmstadt, 64287 Darmstadt, Germany; Cell Biology and Epigenetics, Department of Biology, Technical University of Darmstadt, 64287 Darmstadt, Germany; Cell Biology and Epigenetics, Department of Biology, Technical University of Darmstadt, 64287 Darmstadt, Germany; Cell Biology and Epigenetics, Department of Biology, Technical University of Darmstadt, 64287 Darmstadt, Germany; Cell Biology and Epigenetics, Department of Biology, Technical University of Darmstadt, 64287 Darmstadt, Germany; Faculty of Biology and Center for Molecular Biosystems (BioSysM), Human Biology and BioImaging, LMU Munich, Munich 81377, Germany; Program of Myeloid Neoplasms, Program of Applied Epigenetics, Josep Carreras Leukaemia Research Institute (IJC), Germans Trias i Pujol Research Institute (IGTP), Campus Can Ruti, Camí de les Escoles, 08916 Badalona, Barcelona, Spain; Department of Development and Regeneration, Leuven Stem Cell Institute, Leuven Institute for Single-Cell Omics (LISCO), KU Leuven-University of Leuven, 3000 Leuven, Belgium; Department of Oncological Sciences, Icahn School of Medicine at Mount Sinai, Tisch Cancer Institute, NY, NY 10029, USA; Institute of Molecular Biology (IMB) gGmbH, 55128 Mainz, Germany; Cell Biology and Epigenetics, Department of Biology, Technical University of Darmstadt, 64287 Darmstadt, Germany

## Abstract

MacroH2A has been linked to transcriptional silencing, cell identity, and is a hallmark of the inactive X chromosome (Xi). However, it remains unclear whether macroH2A plays a role in DNA replication. Using knockdown/knockout cells for each macroH2A isoform, we show that macroH2A-containing nucleosomes slow down replication progression rate in the Xi reflecting the higher nucleosome stability. Moreover, macroH2A1, but not macroH2A2, regulates the number of nano replication foci in the Xi, and macroH2A1 downregulation increases DNA loop sizes corresponding to replicons. This relates to macroH2A1 regulating replicative helicase loading during G1 by interacting with it. We mapped this interaction to a phenylalanine in macroH2A1 that is not conserved in macroH2A2 and the C-terminus of Mcm3 helicase subunit. We propose that macroH2A1 enhances the licensing of pre-replication complexes via DNA helicase interaction and loading onto the Xi.

## Graphical Abstract

**Graphical Abstract:**
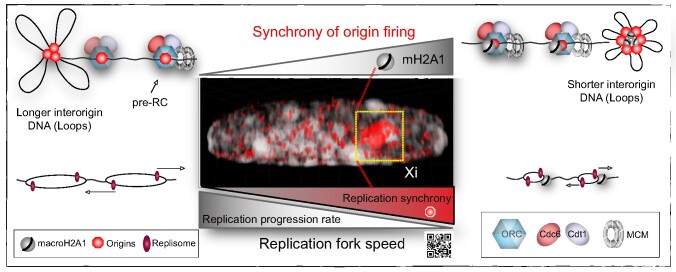

## Introduction

**Figure 1.**
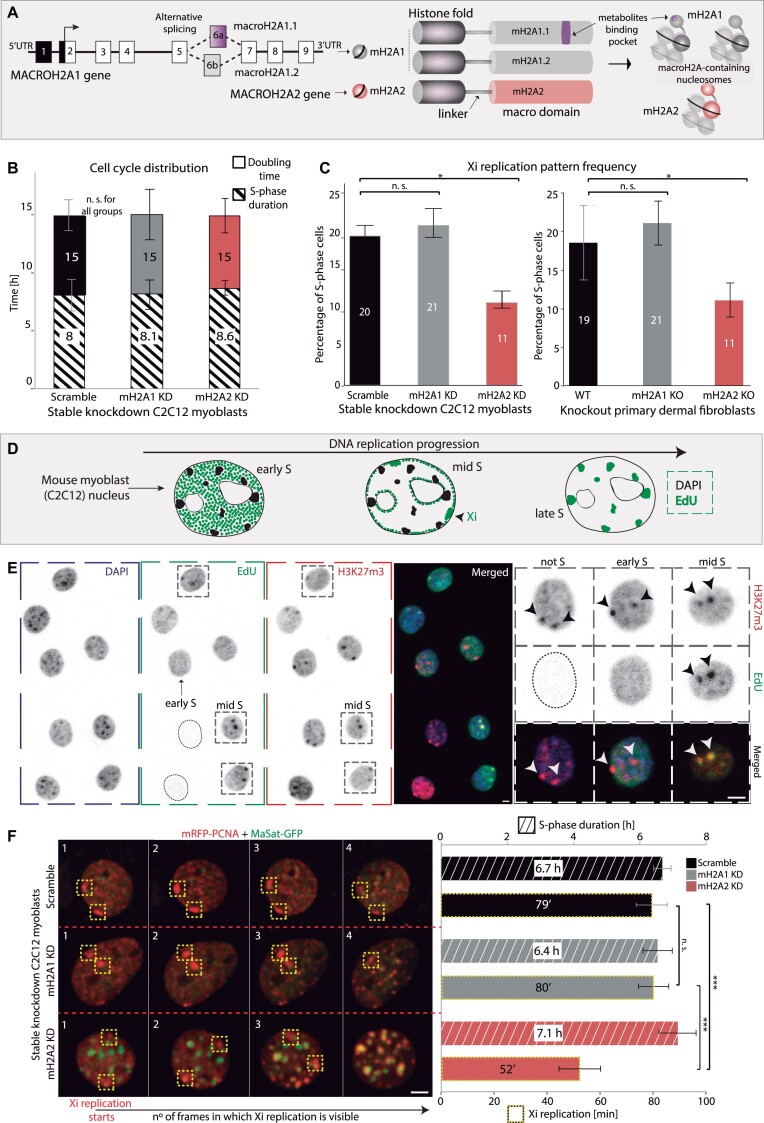
MacroH2A2 depletion results in a lower frequency of Xi replication patterns and faster Xi replication without affecting global cell cycle progression. (**A**) Diagram depicting the structure and splicing of the gene encoding macroH2A1 isoforms (left). Black boxes represent non-coding exons, while white boxes represent coding exons. MacroH2A1.1 specific exon is in purple and macroH2A1.2 specific exon is in grey. To the right, is a schematic representation of the three macroH2A variants and their domain architecture. (**B**) Cell cycle analysis in C2C12 mouse myoblast stable knockdown cell lines: the barplots show the quantification of doubling time and S-phase duration. N-numbers (fixed cells): 673, 551, 449 (Doubling time); *N*-numbers (live-cells): 11 (S-phase duration). Three independent replicates. (**C**) Xi replication pattern quantification is expressed as a percentage from the total S-phase cell population, in both stable knockdown and knockout cell lines for macroH2A. N-numbers (cells)/Replicates: Scramble 1524/9, mH2A1 KD 1492/7, mH2A2 KD 1578/9, WT 521/4, mH2A1 KO 510/3, mH2A2 KO 523/3. (**D**) Scheme showing the well-conserved spatiotemporal dynamics of DNA replication and the different replication patterns over the cell cycle (early, mid, and late), distinguishable by EdU or PCNA (replication machinery component) signal. (**E**) Fluorescence microscopy images of immunofluorescence detection of H3K27me3, EdU replication labeling, and DAPI. Some examples of the different S-phase patterns illustrated in (D) are shown for C2C12 cells, marked with a gray box. A magnified image of a mid-S-phase cell (right) shows replicating Xi chromosomes (black arrowheads) with H3K27me3 accumulation. (**F**) Living cells expressing mRFP-PCNA (red) as a marker for sites of active DNA replication and MaSat-GFP (green) as a marker of late-replicating mouse constitutive heterochromatin (chromocenters) were imaged at 20 min intervals for several hours using a spinning disk confocal microscope. Total replication times and duration of the Xi replication could be visualized and quantified over time by the appearance of the Xi synchronous replication pattern during the mid-S-phase (yellow boxes). N-numbers (live-cells): 4, 6, 6 (S-phase); 32, 26, 28 (Xi replication). Two independent replicates. Barplots show the average value of the distribution and the whiskers represent the standard error with a 95% confidence interval. Statistical significance was tested with a paired two-sample Wilcoxon test (n.s., not significant, is given for *P-*values ≥ 0.05; one star (*) for *P-*values <0.05 and ≥0.005; two stars (**) is given for values <0.005 and ≥0.0005; three stars (***) is given for values <0.0005). *N*-numbers and *P-*values are shown in Supplementary Table 8 (Statistics). Scale bars = 5 μm.

MacroH2A, the largest histone H2A variant (1), has been extensively linked to a repressive chromatin state (2–4) and suggested to be involved in maintaining the silenced state of the inactive X chromosome (Xi) (5,6). A substantial fraction of macroH2A is associated with heterochromatic sequences, specifically on sites enriched in H3K9me3 (7), and large macroH2A-bound regions overlap with H3K27me3 in facultative heterochromatin (8). MacroH2A has the most unique structural organization among histone variants harboring a non-histone region at the C-terminus, the macrodomain, which makes it the biggest known histone (1), together with an N-terminal histone domain, and an unstructured linker (Figure 1A) (9–11). Unsurprisingly, the substitution of the canonical histone for this huge variant (three times the size of H2A) can change the chromatin environment dramatically. Interestingly, macroH2A constitutes a barrier to cellular reprogramming and Xi reactivation. Therefore, this histone variant is considered an important factor in maintaining cell identity, conferring stability to transcriptional states in somatic cells (12–14). MacroH2A knockout mice showed specific effects on the expression of genes related to metabolism and metabolic regulation (15). Due to the similarity of the H2A histone fold domain, macroH2A-containing nucleosomes have an overall similar structure to canonical nucleosomes and span the same DNA length of 146 bp (10,16). Still, differences in a four amino acid sequence in L1 of the histone fold domain, the region responsible for H2A–H2B dimer formation, were proposed to increase the stability of macroH2A-containing nucleosomes. In fact, in contrast to canonical nucleosomes, macroH2A nucleosomes are stable at significantly higher salt concentrations (17,18) and exhibit slower exchange dynamics *in vivo*, as shown by FRAP (fluorescence recovery after photobleaching) analysis (19). This enables stronger DNA compaction and reduction in its accessibility (20). Reduced nucleosome accessibility is related to heterochromatin architecture, affecting DNA repair pathways (7,21) and increasing the stability of repressed chromatin (22). In addition, recent studies have found dysregulated expression of macroH2A in different cancer subtypes, with diverse outcomes depending on the cellular context (reviewed in (23,24)).

Two different genes, *MACROH2A1* and *MACROH2A2* (previously named *H2AFY* and *H2AFY2*), code for three different macroH2A isoforms: macroH2A1.1 and macroH2A1.2 isoforms arise from alternative splicing of a mutually exclusive exon in MACROH2A1, while macroH2A2 is encoded by MACROH2A2 (25) (Figure 1A-left). Interestingly, only macroH2A1.1 can bind adenosine diphosphate ribose (ADP-ribose or ADPR) (26), while macroH2A1.2 and macroH2A2 have no affinity for this metabolite (21,26). In this study, macroH2A1 will be used as a collective term for both isoforms and macroH2A for data concerning all three. Besides the above, little is known about the functional differences between macroH2A1 and macroH2A2. Interestingly, the histone domain of macroH2A1 has the highest structural similarity to the histone fold of the canonical H2A (10). H2A replacement with macroH2A1 tweaks the configuration of chromatin, thus, affecting nucleosome composition and chromatin repositioning to specific nuclear landmarks (27). These chromatin features have a role in regulating the spatiotemporal program of transcription during cell-fate decision (28). Despite this, far less is known about the role of macroH2A during DNA replication, particularly in the duplication of the Xi. In a previous study, we have shown that the Xi chromosome of female mammalian cells starts replicating later than the active homolog and the autosomes. But the Xi is still replicated during the first half of the S-phase when replication of euchromatin takes place, and its replication is highly synchronic (most of the chromosome is duplicated in an average of one and a half hours) (29,30). Subsequent genome-wide analysis of the replication of active versus inactive X chromosomes further validated this outcome (31), with an equivalent origin usage and efficiency for both alleles (32). Being that macroH2A is a hallmark of the X inactivation process, we wondered whether and how its enrichment on this chromosome (33) and accompanying effects on the chromatin environment would determine the extremely fast Xi replication dynamics.

To study the effects of macroH2A variants on DNA replication, we used both knockout primary dermal fibroblasts from macroH2A1 and macroH2A2 knockout mice (13,15), as well as stable knockdown myoblast cell lines, of either macroH2A1 or macroH2A2. In these models, we assessed the total duration of the Xi replication, the synchrony of replication origin firing, and the replication fork speed, to ascertain the role of each macroH2A isoform. Our findings show that macroH2A-containing nucleosomes present an obstacle for *de novo* DNA synthesis on the Xi due to their higher stability. Despite this, only macroH2A2 depletion showed a reduction in the total duration of Xi replication, while macroH2A1 appeared to have no effect. This apparent discrepancy was unraveled by the finding that macroH2A1 positively regulates the synchrony of replication origin firing. The latter occurs through a mechanism that relies on chromatin looping organization and the loading of DNA helicase complexes at the Xi during the G1 phase.

Taken together, we elucidated a novel role of this histone variant in DNA replication, and moreover, the isoform-specific function of macroH2A1 in origin firing at the Xi. This makes the Xi an interesting model to study when replication timing is established early in development and its maintenance in subsequent cell cycles and further differentiated stages.

## Materials and methods

### shRNA plasmids for stable knockdown generation and expression constructs

shRNA plasmids (pSUPER retro puro retroviral vectors) targeting mouse macroH2A1 (pc2802) and 2 (pc2803) as well as the scramble shRNA control (pc2801) were described in (14) and are listed in Supplementary Table 1, together with the expression constructs used for transfections in live-cell and co-immunoprecipitation experiments. To generate mammalian expression plasmids encoding macroH2A1.1-GFP (pc2188), macroH2A1.2-GFP (pc2189) or macroH2A2-GFP (pc2191), the cDNA of each macroH2A isoform was cloned in a pEGFP-N1 backbone (pc0713). To mutate the phenylalanine 192 to valine (F192V) in macroH2A1.1 and macroH2A1.2 (pc5114 and pc5115), and valine 192 to phenylalanine (V192F) in macroH2A2 (pc5116), site directed mutagenesis was performed using a modification of the method described by Kunkel (34). Oligos used to generate macroH2A point mutations are available in Supplementary Table 2. All plasmids were fully sequenced.

### Cell culture, transfection, treatments and cell cycle synchronization

Stable shRNA expressing C2C12 cells (35) (Supplementary Table 3), were cultivated at 37°C, 5% CO_2_ in DMEM (Cat. No.: 41 965 039, Gibco, Massachusetts, USA) supplemented with 20% FBS (Fetal Bovine Serum, Capricorn Scientific, Cat.No.: FBS-22A), 50 μg/ml gentamicin (Sigma-Aldrich, Cat. No.: 1405-41-0), sodium pyruvate 110 mg/l (Sigma-Aldrich, Cat. No.: 113-24-6), and 2 mM l-glutamate (VWR, Cat. No.: 56-85-9). Primary dermal fibroblasts described in (13), derived from macroH2A knockout mice (15), were cultured in DMEM with 10% FBS and 1% penicillin/streptomycin (Sigma-Aldrich, Cat. No.: P0781). Mouse embryonic fibroblasts (MEF) were cultured in DMEM containing 15% FBS (36). HEK Platinum-E retroviral packaging cells ((37) used for retroviral production, were cultured at 37°C, 5% CO_2_ in DMEM supplemented with 10% FBS, 50 μg/ml gentamicin, and 2 mM l-glutamate. All cells were frozen in freezing media (DMEM supplemented with 20% FBS, 50 μg/ml gentamicin and 2 mM l-glutamate, 10% DMSO), and were regularly tested for mycoplasma to ensure that they were contamination-free. All the references and details are given in Supplementary Table 3 (cell lines).

For live-cell experiments, stable knockdown C2C12 cells were transfected by electroporation with the AMAXA Nucleofector system II (Lonza, S/N: 10 700 731), using a self-made buffer (5 mM KCl (Sigma-Aldrich Cat.No.: 7447-40-7), 15 mM MgCl_2_ (Sigma-Aldrich Cat. No.: 7786-30-3), 120 mM Na2HPO4/NaH2PO4 (Sigma-Aldrich Cat. No.: 7558-79-4) pH 7.2, 50 mM Mannitol (Caesar & Loretz, Cat. No.: 69-65-8)) (38) with default program B032. For co-immunoprecipitation experiments, C2C12 cells were transfected with polyethyleneimine (PEI) (pH-10, Cat. No.: 40827-7, Sigma-Aldrich Chemie GmbH, Steinheim, Germany) as previously described (39), and to enrich the protein lysate in cells in G1/S-phase stage, synchronization was performed by double thymidine arrest (40) (Sigma-Aldrich, Cat.No.: 50-89-5) to a final concentration of 2 mM. Cells were processed for co-immunoprecipitation 7 h after release into growth medium (DMEM supplemented with 20% FBS, 50 μg/ml gentamicin, sodium pyruvate 110 mg/l and 2 mM l-glutamate) without thymidine, approximately 48 h after transfection. The cell cycle synchronization procedure in M/G1 to study Mcm2/ORC1/Cdc6/Cdt1 levels throughout G1 was performed exclusively by mitotic shake-off of loosely adherent mitotic cells and without drug treatment. After collection, cells were centrifuged, the pellets were resuspended in warm medium and seeded on gelatin-coated glass coverslips. Cells growing onto coverslips were processed for immunofluorescence in one-hour intervals (see ‘Immunofluorescent visualization’ section). The cell cycle stage of the cells was verified by measuring their DNA amount (DAPI sum intensity, see section below), which was always within the value range expected for cells in G1.

### C2C12 stable knockdown cells generation: production of retrovirus and infection of cells

Retroviral production and infection were performed as described previously (41), implementing minor modifications. First, HEK Platinum-E retroviral packaging cells were seeded at 0.6 × 10^6^ cells per p100 plate (Cat. No.: 83.3902, Sarstedt, Nümbrecht, Germany). After 24 h of seeding, 30 μg pSUPER retro puro retroviral vectors in 900 μl DMEM without antibiotics were mixed, and incubated for 30 min with 90 μl polycation polyethyleneimine (PEI) (pH 7, Cat. No.: 40827-7, Sigma-Aldrich Chemie GmbH, Steinheim, Germany) in 900 μl DMEM without antibiotics. This mixture was added dropwise onto the cells. Control for retrovirus production was performed by transfection with the fluorescent protein Tomato (pc2804). Twenty-four hours after that, fresh medium was added to the Plat-E cells. Seventy-two hours post-transfection viral particles were collected, filtered through a 0.45 μm pore size cellulose acetate filter (Cat. No.: 1110647ACN, Sartorius, Goettingen, Germany) and supplemented with 10 μg/ml polybrene (Cat. No.: TR1003-G, Merck Millipore GmbH, Darmstadt, Germany). C2C12 cells were seeded 24 h before infection at 5 × 10^4^ cells per well in a 6-well plate. The mix of retrovirus-containing medium and polybrene was added to the C2C12 cells for 24 h. Antibiotic selection was started 24 h after infection with 2 μg/ml puromycin (InvivoGen, Cat, No.: ant-pr-1) for 24 h. The cells were expanded for a week and then used for experiments or frozen in freezing media (DMEM supplemented with 20% FBS, 50 μg/ml gentamicin, and 2 mM l-glutamate, 10% DMSO) for storage at −150°C.

### RNA extraction, cDNA synthesis and qPCR

Total RNA of 2.5 × 10^6^ cells were extracted using the RNAeasy kit (Qiagen, Germantown, USA, Cat. No.: 74 104) following the manufacturer's instructions. 4 μg RNA from each cell line was converted to cDNA using 250 ng of random 9-mer primers (Agilent Technologies, Boeblingen, Germany) and the SuperScript First-Strand Synthesis System (Invitrogen, Carlsbad, USA) according to the manufacturer's instructions. qPCR was performed using EXPRESS SYBR GreenER qPCR supermix with premixed ROX (Invitrogen, Carlsbad, USA) following the manufacturer's instructions in a StepOne real-time PCR system (Applied Biosystems, Foster City, USA). For each cell line, 1 μl of the reverse transcription reaction product was used as input for qPCR. Primers for macroH2A1 and macroH2A2 were published in (42), primers used for GAPDH normalization control were: GAPDH-forward: CCA TAC ATA CAG GTT TCT CCA G and GAPDH-reverse: CTG GAA AGC TGT GGC GTG ATG G (43). PCR conditions were set as follows: pre-incubation at 50°C for 2 min, denaturation at 95°C for 2 min, 40 cycles of 95°C for 15 s and 60°C for 45 s followed by a final extension at 72°C for 7 min and a melting curve analysis. qPCR products were analyzed on a 1% agarose gel stained with ethidium bromide to a final concentration of 0.5 μg/ml, and imaged with the Amersham AI600 Imager (GE Healthcare, Chicago, IL, USA).

### Co-immunoprecipitation, chromatin fractionation, SDS PAGE and western blot

For Western blot analysis, cells were trypsinized, counted to 5 × 10^5^ cells per lane, and lysed for 15 min on ice in 800 mM NaCl containing lysis buffer (20 mM Tris–HCl (pH 8), 1.5 mM MgCl_2_, 0.2 mM EDTA, 0.4% NP-40 and protease inhibitors). The lysate was drawn 10 times through a 21G needle, incubated on ice for 25 more minutes, and diluted to 400 mM NaCl. This lysate was cooked in Laemmli buffer for 10 min at 95°C. Co-immunoprecipitations were essentially performed as described before (38). In brief, C2C12 cells growing in 100 mm dishes were PEI-transfected (macroH2A-GFP vectors, Supplementary Table 1) and harvested by centrifugation 48 h later at 90% confluence to ensure that overexpressed macroH2A histone were incorporated into chromatin (44). The cell pellet was washed with ice-cold 1× PBS (phosphate-buffered saline: 137 mM NaCl, 2.7 mM KCl, 10 mM Na_2_HPO_4_, 1.8 mM KH_2_PO_4_, in ddH_2_O, pH ∼ 6.8) and pelleted again. The supernatant was discarded and the pellet resuspended in 200 μl lysis buffer (20 mM Tris–HCl pH 8, 150 mM NaCl, 0.5 mM EDTA, 0.5% NP-40) supplemented with pepstatin A (1 μM; Sigma-Aldrich, St. Louis, MO, USA), PMSF (10 μM, Sigma-Aldrich, St. Louis, MO, USA) and AEBSF (1 mM, AppliChem, Darmstadt, Germany). Cells were homogenized with a syringe (21G needle, 20 strokes) and incubated on ice for 30 min with repeated vortexing in between. Lysates were then cleared by centrifugation for 15 min at 13 000 × g and 4°C. 15% of the lysate was used as input and the rest was incubated with GFP-binder beads produced as described before (45) on a rotator at 4°C for 90 min Afterward, the beads were washed three times with 500 μl washing buffer. Input and bound fractions were boiled at 95°C in 4× SDS loading buffer (200 mM Tris/HCl pH 6.8, 400 mM DTT, 8% SDS, 0.4% bromophenol blue and 40% glycerol), separated on 8% SDS-PA (sodium dodecyl sulfate–polyacrylamide) gels. For chromatin fractionation experiments, samples were prepared using the protocol described in (46). SDS-PAGE and Western blotting were performed as described in (47). Co-immunoprecipitation samples, cell lysates, or cellular fractions were transferred onto a nitrocellulose membrane (GE Healthcare, München, Germany). Blocking of membranes was performed for one hour in 3% low-fat milk in 1× PBS, followed by incubation with primary antibodies diluted in blocking buffer overnight at 4°C. The following antibodies were used: chicken monoclonal anti-macroH2A1 H032 (48), rabbit polyclonal anti-histone H3 (Upstate, Lake Placid, USA, diluted 1:10 000), rabbit polyclonal anti–macroH2A2 (Invitrogen, Thermo Fisher Scientific, Germany, diluted 1:500), mouse monoclonal anti-tubulin (alpha) (Sigma-Aldrich, USA, diluted 1:5000), rabbit monoclonal anti-Mcm2 (Abcam, diluted 1:8000), rabbit polyclonal anti-Mcm3 (1:1000), rabbit polyclonal anti-Mcm4 (1:1, 000), rabbit polyclonal anti-Mcm5 (1:1000), rat monoclonal anti-GFP (Chromotek, diluted 1:1000). For endogenous co-immunoprecipitation of Mcm4, C2C12 cells were transfected and synchronized in G1/S-phase stage as described before. 15% of the cell lysate was used as input and the rest was incubated for two hours with Pierce™ Protein G agarose beads (ThermoFisher Scientific, Cat. No.: 20 390) preincubated with antibodies against Mcm4 or MIN (attP synthetic peptide) as negative control for immunoprecipitation. The membrane was incubated with the respective secondary antibodies after washing using 1× PBS supplemented with 0.01% Tween-20. Horseradish peroxidase (HRP) conjugated sheep anti-mouse IgG (Amersham Pharmacia Biotech, United Kingdom), goat anti-rabbit IgG (Sigma-Aldrich, USA), and goat anti-rat IgG (The Jackson Laboratory, USA) were used (1:5000). Alternatively, incubation with fluorescently tagged secondary antibodies was performed in chromatin fractionation experiments: Cy5-conjugated donkey anti-mouse IgG (H + L), Cy3-conjugated donkey anti-mouse IgG (H + L), Cy5-conjugated donkey anti-rabbit IgG (H + L) and Cy3-conjugated donkey anti-rabbit IgG (H + L) (1:2000; The Jackson Laboratory, Bar Harbor, ME, USA). Characteristics of all primary and secondary antibodies, as well as dilutions used, are described in Supplementary Table 4. For imaging, the Amersham AI600 Imager was used (GE Healthcare, Chicago, IL, USA). Cutouts of the membranes were made in some cases for better composition of the figures. Uncropped and unprocessed scans for all the blots are available, including replicates in supplementary figures, and are provided with the data sets uploaded to https://doi.org/10.48328/tudatalib-1344.2.

### DNA replication and (immuno)fluorescent visualization

Immunostainings were performed as described in (49). For this purpose, cells were seeded on gelatin-coated glass coverslips. To label sites of DNA synthesis/replication, cells were grown in a medium supplemented with 10 μM 5-ethynyl-2′-deoxyuridine (EdU) (Invitrogen, Carlsbad, USA) for the indicated periods before fixation. For example, for counting of replication foci, incubation with EdU was performed for 20 min, followed by washing with cold 1× PBS before fixation. As a control for the aphidicolin treatment, cells were labeled with 100 μM 5-bromo-2-deoxyuridine (BrdU) (Invitrogen, Carlsbad, USA) for 10 min. Afterward, cells were washed with 1× PBS and fixed in 3.7% formaldehyde (Sigma-Aldrich Chemie GmbH, Steinheim, Germany, Cat.No.: F8775) in 1× PBS for 10 min. For Mcm2, Mcm2-phosphoS108, ORC1, Cdc6 and Cdt1 immunostainings, samples were extracted before fixation following the protocol from (50) to eliminate soluble proteins and ensure the detection of chromatin-loaded fractions. Briefly, cells were extracted with 0.1% Triton X-100 in CSK buffer (10 mM pipes-KOH, pH 7.0, 100 mM NaCl, 300 mM sucrose, 3 mM MgCl_2_) on ice for 5 min. Then, the extraction solution was carefully replaced by 2% formaldehyde in the CSK buffer for 30 min. After fixation and three washing steps with PBS-T (1× PBS, 0.01% Tween-20), cells were permeabilized with 0.5% Triton X-100 in 1× PBS for 20 min. Only for PCNA immunostaining, after permeabilization cells were incubated in ice-cold 88% methanol in ultra pure H_2_O (v/v) for 5 min for antigen retrieval and washed again. Before incubation with primary antibodies, blocking was performed for 40 min in 2% bovine serum albumin in 1× PBS at 37°C in a humid chamber. Primary antibody incubation was performed in 2% BSA in 1× PBS for 1.5 h at 37°C. BrdU was detected with a rat anti-BrdU antibody (1:100, Serotec) diluted in buffer consisting of a 1:1 mixture of blocking and 2 × DNase I reaction buffer (60 mM Tris/HCl pH 8.1, 0.66 mM MgCl_2_, 1 mM beta-mercaptoethanol) and 25 U/ml DNase I (Cat. No.: D5025, Sigma-Aldrich Chemie GmbH, Steinheim, Germany). DNase I digestion was stopped by washing with PBS–TE (PBS-T with 1 mM EDTA). After incubation with the primary antibodies, cells were washed three times with PBS-T, and for the detection of the primary antibodies, cells were incubated with fluorescently tagged secondary antibodies diluted in 2% BSA: donkey anti-rat IgG AlexaFluor 488, Alexa Fluor 488-conjugated goat anti-rabbit IgG (H + L) (1:500), Alexa Fluor 488-conjugated goat anti-mouse IgG (H + L) (1:500), Alexa Fluor 594-conjugated goat anti-rabbit IgG (H + L) (1:250; ThermoFisher Scientific, Invitrogen, Carlsbad CA, USA, Cat. No.: R37117), Cy5-conjugated donkey anti-mouse IgG (H + L) (1:250; The Jackson Laboratory, Bar Harbor, ME, USA, Cat. No.: 715–715-150). After 45 min of incubation with secondary antibodies solution at room temperature, cells were washed three times with PBS-T. After the incubation with the secondary antibodies, the detection of incorporated nucleotides was performed for samples incubated with EdU before fixation. This was performed using the Click-IT assay following the manufacturer instructions, preparing a dilution containing 1:200 3-azido-7-hydroxycoumarin, 1:1, 000 6-carboxyfluorescein (6-FAM azide) or 1:2000 5/6-sulforhodamine azide (Cat. No.: 7811, 7806 and 7776 respectively, Carl Roth, Karlsruhe, Germany). The samples were incubated with the Click-IT mix for 45 min at RT (room temperature), followed by three washing steps in PBS-T. Finally, DNA was counterstained with DAPI (4,6-diamidino-2-phenylindole, 10 g/ml, Cat. No.: D27802, Sigma-Aldrich Chemie GmbH, Steinheim, Germany) for 10 min and samples were mounted in Mowiol 4–88 (Cat. No.: 81 381, Sigma-Aldrich Chemie GmbH, Steinheim, Germany) containing 2.5% DABCO (1,4-diazabicyclo[2.2.2]octane, Cat. No.: D27802, Sigma-Aldrich Chemie GmbH, Steinheim, Germany). Samples for structured illumination microscopy (3D SIM) were mounted in Vectashield (Invitrogen, Carlsbad, CA, USA). All the information on modified nucleotides is shown in Supplementary Table 5, and primary and secondary antibodies are described in Supplementary Table 4.

### Probe generation and X (Repli-)/(Halo-) FISH (fluorescence *in situ* hybridization)

The probe against the X chromosome was prepared by microdissection combined with DOP-PCR (degenerated oligonucleotide-primed-PCR) labeling method. For template stock generation, mouse X chromosome-specific template, 2 μM 6MW primer (5′-CCGACTCGAGNNNNNNATGTGG-3′) (51), 0.25 mM dNTPs and 2.5 U Taq polymerase in 1× PCR buffer (10 mM Tris/HCl pH 8.3, 50 mM KCl and 1.5 mM MgCl_2_) and cycling conditions were set to (5 min at 94°C) × 1, (45 s at 94°C, 45 sec at 15°C, 12 min at 37°C) × 1, (40 s at 94°C, 45 s at 37°C, 4 min at 66°C) × 5 and (40 s at 94°C, 45 s at 54°C, 4 min at 66°C) × 24. X chromosome template DNA was labeled with biotinylated nucleotides (52): the template stock DNA was mixed with a nucleotide mixture containing unlabeled nucleotides (0.2 mM each dATP, dCTP, and dGTP with 0.1 mM dTTP), biotinylated dUTPs (0.1 mM biotin-16-dUTPs), 2 μM 6MW primer, 2.5 U Taq polymerase, and 1 × PCR buffer, and PCR reaction was set to (5 min at 94°C) × 1, (30 s at 94°C, 30 s at 54°C, 90 min at 72°C) × 35 and (5 min at 72°C) × 1. Fluorescence *in situ* hybridization, combined with immunofluorescence and DNA replication labeling, was made as described in (52). X-FISH visualization was performed using streptavidin Cy3 (1:500, Thermo Fisher Scientific, Waltham, MA, USA) in blocking buffer. Visualization of DNA replication (EdU) and immunostained proteins was performed as described before. Fluorescence *in situ* hybridization after DNA Halos preparation (see section below), was performed by adapting the protocol from (53). Briefly, after DNA Halo preparation cells were incubated with RNase A 10 μg/ml for 30 min at 37°C, followed by a washing step with ice-cold HCl 0.05 N, and incubation on ice for 5 min. Then, washing with PBS 1× and fixation with 2% paraformaldehyde for five min. After this fixation, cells were washed twice with SSC 2× (stock 20×: NaCl 3 M, sodium citrate tribasic dihydrate 300 mM, in ultra pure H_2_O), and dehydrated by immersion in 70% ethanol, 80% ethanol and 100% ethanol, 2 min each, letting the coverslips dry at the end. Coverslips were co-denatured with the FISH probe by adding five μl of the probes onto the cells, mounting the hybridization chamber, incubating at 37°C for 15 min, followed by incubation in a water bath at 80°C, and incubation in ice for 5 min. After 5 min on ice, cells were incubated with the probe overnight at 37°C, followed by three washing steps with SSC 2 × 0.05% Tween-20, blocking with 2% BSA in PBS 1× for 30 min, and incubation with the detection solution (streptavidin Cy3 1:500 in blocking buffer) for 1 h at room temperature. All primers, antibodies and nucleotide characteristics are summarized in Supplementary Tables 2, 4 and 5, respectively.

### Doubling time, S-phase duration and Xi replication pattern frequency

For growing curve analysis, 2 × 10^5^ knockdown and knockout cells were seeded as technical triplicates at day 0, and cell numbers were counted with a Neubauer hemocytometer for four consecutive days. Population doubling times were derived with log*2*(*n_x_*/*n*_0_)/*t* (h) (*n_x_*: cell number at day *x*, *n*_0_: cell number at day 0, *t*: hours after seeding). To determine the percentage of cells in every cell cycle and S-phase substage, asynchronously growing cell cultures were pulse-labeled with 10 μM EdU for 12 min, fixed and EdU was detected as described before. Cells were manually counted and grouped into S-phase substages (early, mid or late), non-S-phase or mitosis, and the fraction of each cell cycle stage was calculated as a percentage. Specifically for mid-S-phase cells, the fraction of the Xi replicating pattern was verified using H3K27me3 signal as a marker for Xi. The S-phase duration was derived by multiplying the doubling time with the percentage of cells in the respective phase.

### Hypotonically resolved replication foci

In this study, Xi replication foci were hypotonically resolved and counted using the protocol described in (54). Briefly, after EdU incubation (20 min) and trypsinization of the cells, the cell pellets were resuspended in 1–5 ml of pre-warmed hypotonic solution (75 mM of KCl in ddH_2_O freshly made) depending on the cell density. Then, cells were incubated in this solution for 20 min at 37°C and, then, cyto-spined at 1800 rpm for 6 min to attach the cells to the microscope slides. This process significantly increased their flatten morphology and chromatin decondensation. After cytospin, cells were fixed with 3.7% formaldehyde in 1× PBS for 10 min and stained as described before.

### Preparation of fluorescent DNA halos

DNA Halo preparations were largely performed according to (55). This method detects DNA loops by extracting histones with a high-salt incubation followed by loop extrusion out of the nuclear scaffold in the presence of a DNA dye (56,57). The extracted loops can be distinguished from a densely stained central chromatin core or nuclear scaffold, providing a measure of their size. Briefly, cells were trypsinized and cell pellets were washed in 1 × PBS. After that, cells were resuspended in nuclei buffer (10 mM Tris at pH 8, 3 mM MgCl_2_, 0.1 M NaCl, 0.3 M sucrose, protease inhibitors) plus 0.5% Nonidet P40 for 10 min on ice. Cells were attached to coverslips using cytospin (1800 rpm for 6 min); stained with 2 mg/ml DAPI for four min; and immersed in a buffer containing 25 mM Tris (pH 8), 0.5 M NaCl, 0.2 mM MgCl_2_, 1 mM PMSF, and protease inhibitors for 1 min, then in Halo buffer (10 mM Tris at pH 8, 2 M NaCl, 10 mM ethylene diamine tetraacetic acid [EDTA], 1 mM DTT, protease inhibitors) for 4 min. Next, cells were washed in a buffer containing 25 mM Tris (pH 8), 0.2 M NaCl and 0.2 mM MgCl_2_ for 1 min, and in the same buffer omitting the NaCl for 1 min. All these steps were performed on ice. Finally, cells were fixed in 2% formaldehyde for 10 min, washed twice in 1× PBS, and mounted on slides with Vectashield® antifade medium (Cat. No.: NC9265087, Thermo Fisher Scientific, Waltham, USA).

### Proximity ligation assay (PLA)

*In situ* interaction between macroH2A isoforms and Mcm2 was quantified by proximity ligation assay using the duolink® kit (Sigma-Aldrich, DUO92101), following the manufacturer's instructions and the protocol described in (58) and (59). In this technique, small oligonucleotide probes, (+) and (−), conjugated to secondary antibodies specifically recognize the primary antibodies against the proteins of interest. When the two probes are closer than 40 nm, ligation by ligase incubation can occur. This generates circular DNAs that will be amplified by a polymerase incorporating fluorescently labeled nucleotides. Afterward, fluorescent spots can be detected and quantified using microscopy and image analysis, considering each spot an interaction site between the two proteins. Briefly, immunostaining against macroH2A1 or macroH2A2 and Mcm2 was performed as described before, but after incubation with primary antibodies and subsequent washing steps, fluorescent-tagged secondary antibodies were replated with duolink® PLA reagents (*in situ* complementary oligonucleotide probe MINUS (−) and PLUS (+)). Incubation with only one of the primary antibodies (anti-Mcm2) was used as a negative control for the assay. The PLA probes were mixed and diluted (1:5) in antibody diluent (2% BSA in 1× PBS), incubated at room temperature for 20 min, and then incubated with the samples of interest for 1 h at 37°C in a humid chamber. Then, samples were washed two times with washing buffer A (0.01 M Tris, 0.15 M NaCl, 0.05% Tween-20), and the probes were ligated with two other circle-forming DNA oligonucleotides by ligation-ligase solution for 30 min at 37°C. After this incubation and washing steps, amplification of the oligonucleotides was performed via the rolling circle by incubation with amplification-polymerase solution (nucleotides and fluorescently labeled oligonucleotides together with polymerase) for 90 min at 37°C. During this incubation, the fluorescent oligonucleotides hybridize into the rolling-circle amplification product making the signal visible as a fluorescent spot by microscopy. Finally, samples were washed with washing buffer B (0.2 M Tris, 0.1 M NaCl) 2 times × 10 min, once in 1× PBS, and finally, DNA was counterstained with DAPI and mounted with Mowiol as described for immunostainings.

### Microscopy

Characteristics of the microscope systems, including laser, filters, and objectives used, are summarized in Supplementary Table 6.

*Live cell microscopy*. To perform the live-cell experiments stable knockdown C2C12 cells were transfected with mRFP-PCNA (60) and MaSat-GFP (61) as described before. Four-dimensional time-lapse experiments were carried out on an UltraVIEW VoX spinning disc confocal system (PerkinElmer, UK) in a closed live-cell microscopy chamber (ACU control, Olympus, Japan) set to 37°C, 5% CO_2_ and 50% air humidity, mounted on a Nikon Ti microscope (Nikon, Japan). Image acquisition was performed every 20 min using a 60×/1.45 NA Plan-Apochromat oil immersion objective lens (pixel size in XY = 111 nm). Images were obtained with a cooled CCD camera (Hamamatsu Photonics K.K., Hamamatsu City, Japan, Cat.No.: C9100-50), Z-step = 0.3–1 μm.

RPA accumulation as a proxy for DNA helicase activity was performed as described in (62). In brief, stable knockdown C2C12 cells were transfected with mRFP-PCNA (60) and GFP-RPA (63), and time-lapse microscopy was carried out as described above. Cells were imaged once before adding aphidicolin (A0781, Sigma Aldrich, St Louis, MO, USA) to a final concentration of 50 μg/ml (150 μM) or DMSO (41 639, Sigma Aldrich, St Louis, MO, USA) as a control, and then imaged every minute for 30 min.

*Structured illumination microscopy (3D SIM) and confocal imaging*. To analyze replication foci in 3D super-resolution microscopy (3D SIM) and confocal microscopy, samples were prepared as described (54,64). 3D SIM images were acquired with a DeltaVision OMX V3 system (65) as described in (66). Confocal images were collected in a Leica TCS SP5II confocal laser scanning microscope (Leica Microsystems, Wetzlar, Germany) equipped with an oil immersion Plan-Apochromat 100×/1.44 NA objective lens (pixel size in XY set to 50 nm, Z-step = 290 nm). The latter microscope was also used for the acquisition of images in EdU/PCNA ratio experiments, PLA assay, and several immunostaining experiments. Additionally, for Mcm2 loading curves we used an UltraVIEW VoX spinning disc system (PerkinElmer, UK) on a Nikon Ti microscope (Nikon, Japan) described before equipped with an objective 100×/1.49 NA CFI Apochromat TIRF oil immersion (voxel size, 0.071 × 0.071 × 0.5–1 μm; Nikon, Tokyo, Japan) and a cooled 14-bit CCD camera.

*High content and widefield microscopy of fixed samples*. DNA Halos were imaged using a widefield fluorescence microscope Zeiss Axiovert 200 (Zeiss Axioplan 2, 100×/1.30 NA Plan-Neofluar Oil Ph3 objective; Axiovision software (version 4.8.2.0 SP3); AxioCam mRM camera). Nuclear levels of macroH2A1, Mcm2, Mcm2-phosphoS108, nuclear roundness as morphological property of the nuclei, and the number of nuclear spots for PLA assay, were measured with the Operetta high-content screening system (Perkin Elmer, UK) in wide-field mode, equipped with a Xenon fiber optic light source and a 20×/0.45 NA long working distance or a 40×/0.95 NA objective. For excitation and emission, the following filter combinations were used, 360–400 nm and 410–480 nm for DAPI, 460–490 nm and 500–550 nm for Alexa-488 as well as 560–580 nm and 590–640 nm for Alexa-594. Fluorescence intensity levels were quantified with the Harmony software (Version 3.5.1, PerkinElmer, UK).

### Image analysis

*Quantification of histone modifications (H3K27me3, H3K9ac, H4K8ac, H4K20me1/2/3) and macroH2A1 levels*. The levels of macroH2A1 and the different histone modifications were quantified in confocal images from knockdown and/or knockout cells using FIJI. First, H3K27me3 levels were measured in the nucleus by segmTableon using the DAPI signal to create a binary mask (Auto-thresholding, Triangle method). Also, H3K27me3 enrichment in the Xi was observed in all cell lines, thus, H3K27me3 was used for Xi segmentation thereafter. The same process described before was applied for image analysis: in the multicolor Z-stack images DAPI channel was selected to create a mask containing the full nucleus by thresholding (Triangle method). Using H3K27me3 signal, a mask was created for the Xi using the same thresholding method. Based on these ROIs (region of interest) generated with the different masks, we quantified the fluorescence intensities in the channel corresponding to histone modification signals, and on the respective regions (Nuclear versus on Xi). For macroH2A1 and histone modification levels, fluorescence means or sum intensity values (RawIntDen) were normalized by the average of the control cells (‘Scramble’ for knockdowns and ‘WT’ for knockout cells).

*Quantification of Xi copy number*. Microscopy data sets with H3K27me3 immunostaining were analyzed to quantify the number of Xi chromosomes in control and macroH2A knockdown cell lines. Cells showing homogenous nuclear distribution of H3K27me3 were not considered in this analysis. From the percentage of cells showing a clear H3K27me3 accumulation in the Xi (72–80%), the number of Xi clusters (based on H3K27me3 signal) was counted for each cell, classifying them as 1-Xi, 2-Xi, >2-Xi. No cells were found with more than two Xi (>2-Xi). Therefore, the percentage of cells showing two Xi chromosomes from the total of cells analyzed was plotted for each cell line as a barplot.

*Replication fork speed (Edu/PCNA ratio) and proxy for helicase speed on Xi*. To assess the speed of DNA synthesis on the Xi and to compare it with global fork speed rates, we measured the incorporation of nucleotides (EdU signal) relative to the replication machinery (PCNA) on the Xi and on the full nucleus. To this end, knockdown and knockout cell lines were imaged using confocal microscopy, and multicolor Z-stacks were processed using FIJI. Briefly, we created masks comprising the synchronously replicating Xi. Then, we normalized the total nucleotide signal to the total PCNA signal (Sum intensities) using the image analysis platform Priithon (http://code.google.com/p/priithon/). Alternatively, for each cell, we generated the ROI containing the replicating Xi using FIJI, and the sum intensities were measured in both EdU and PCNA channels. Afterward, EdU/PCNA ratios were calculated for each cell line, normalizing by the average of control cells (Scramble or WT), and plotted. The same image analysis pipeline was performed for the full nucleus, generating nuclear ROIs and measuring fluorescence intensities to obtain global nuclear values of EdU/PCNA ratios.

Helicase speed on the Xi (analysis of the focal RPA accumulation upon aphidicolin/DMSO treatment was performed as described in (62). Only mid-S-phase cells showing a Xi replication pattern were chosen for analysis. Briefly, cell nuclei were segmented using the Volocity software (Version 6.3, Perkin Elmer), GFP-tagged RPA intensities were measured, and the coefficient of variation (*c*_V_ = σ/μ, with σ = standard deviation and μ = mean), as a proxy for helicase speed/activity was calculated for all time-points. All values were normalized to the pretreatment *c*_V_ = *c*_V_(*tp*_x_)/c_V_(tp_0_) with *tp*_x_: any given time point imaged, *tp*_0_: pretreatment time point) and plotted using RStudio (version 1.1.447). To ensure the complete inhibition of the DNA polymerase by the aphidicolin, cells were incubated with 10 μM BrdU for 10 min directly after imaging and fixed in 3.7% formaldehyde. BrdU detection and immunostaining were performed as described before, and confocal images were analyzed for present/absence or BrdU signal.

*Quantification of Xi replication foci numbers*. This quantification was mainly performed as described in (54,64). Structured-illumination microscopy images (3D-SIM) were used to count the replication foci on the Xi (nanoRFi). First, 3D-SIM images were reconstructed, and exported from the DeltaVision software (soft-WoRx 6.0 Beta 19, Applied Precision) and raw 3D-SIM images were converted to 16-bit images using a custom-written FIJI (67) macro. The images were segmented in FIJI (Triangle method) and an ROI comprising the Barr body, defined by DAPI, was selected manually. H3K27me3 and EdU signals were used to validate the accurate selection of the Xi. The resulting ROIs were transformed into binary images that were used to mask the original replication foci signals of interest and to discriminate them from the background (set to ‘0′). Secondly, these images and the corresponding DAPI images were imported to the image analysis software Volocity 6.3 (Improvision, PerkinElmer, UK) and replication foci were quantified for individual Xi. Specifically, 3D-SIM replication foci were detected by intensity excluding only black pixels (i.e. background with intensity ‘0’), touching foci were separated (object size guide = 0 μm^3^) and signals smaller than 0.0002 μm^3^ were excluded from the final counting as they represented unspecific background signal. Only foci within the Xi ROI were counted.

The analysis of hypotonic resolved RFi imaged by confocal microscopy was performed using FIJI. First, the validation of the hypotonic treatment was analyzed using DAPI profiles, measuring morphological properties of segmented 3D nuclei (diameter, surface area and z-axis length) in Volocity software (Version 6.3, Perkin Elmer). Additionally, chromatin decondensation/relaxation was analyzed using DAPI standard deviation values (68) and quantitative analysis of the 3D nuclear landscape (Nucim)(69). After using FIJI for image processing, RStudio was used for image analysis with Nucim, which assessed seven different chromatin compaction levels in individual cell nuclei using DAPI as a proxy for local differences in chromatin compaction. These tools are freely available in open-source R packages ‘nucim’ and ‘bioimagetools’.

Secondly, for RFi quantification, the Xi was segmented using H3K27me3 signal or X(i)-FISH and validated with the presence of the Xi replication pattern (EdU). In this quantification, the analysis relied on the identification of local maxima of intensity. Therefore, the influence of random noise (1-pixel spikes in intensities) on local maxima selection needs to be reduced. For this purpose, we applied a smoothing filter (Gaussian blur) using a kernel size of 1 pixel. Then, the contrast of the image was enhanced by choosing a linear stretching of the histogram. Finally, for the identification of local maxima in the 3D stack, we used the FIJI plugin ‘Foci_Picker3D’ (Version 1.0). Each focus in the Xi Z-stack is represented by one and only one local maximum center. A maximum center is one pixel or a group of contiguous pixels with the same intensity, which is bigger than all the surrounding pixels. The algorithm of this plugin has two input parameters: background intensity around the focus, and ‘Fraction f’, a threshold in percentage that defines the brightest fraction of a focus. Once parameters like ‘Tolerance’ (minimum intensity range of pixels inside the focus), ‘Minimum’ (Minimum volume/area one focus contains in pixels), and shape (minimum radius of the particle) are selected, the Foci_Picker3D algorithm looks for the maximum centers and expands from the center to the edge of the focus defined by the minima pixels. Characteristics like volume, area, intensity, and coordinates of the recognized objects (foci) are analyzed and the objects are shown with different colors in a separate image stack. The number of objects found within the Xi for each cell, using the same parameters for all samples, was taken as the number of hypotonically resolved RFi.

*Analysis of DNA Halos and X (Halo-)FISH*. Nuclear scaffolds and the DNA Halos were imaged using a wide-field microscope (Zeiss Axioplan 2, 100×/1.30 NA Plan-Neofluar Oil Ph3 objective; Axiovision software (version 4.8.2.0 SP3); AxioCam mRM camera). The image analysis and measurements of the DNA Halos size were performed as described in (70): The total area (At) and nuclear scaffold area (As) of each cell were thresholded and segmented using FIJI and the DNA Halo area (Ah) was calculated as a subtraction of the two (Ah = At – As). Finally, the DNA Halo radius was derived with the formula: *R* = √ (*Ah*/π). DNA Halo circularity was calculated by FIJI with the formula: circularity = 4π(area/perimeter^2^). The analysis of X (Halo-)FISH was performed with FIJI (ImageJ) using the line profile as follows: first, the border of the nuclear scaffold (DAPI channel) was settled as starting point ‘0’ to draw a line of 30 microns length and approximately 90° with the tangent of the nuclear scaffold border. Fluorescence intensities for X-FISH (Cy3 channel) were measured for each point (pixel) of the line, and later on normalized by the maximum intensity value for each cell. Average normalized intensities for all cells analyzed were calculated for each point of the line and plotted as a line plot from 0 to 20 microns since, after this length, only intensity background levels were measured. Background levels were calculated by the average of random line profile analysis in areas without cells/FISH signal performed in different images for all samples.

*Analysis of the Xi relative area*. Not replicating cells in G1 (without EdU signal) were selected for this analysis to avoid side effects of replication in chromatin structure. G1 cells were selected based on their DNA amount (DAPI sum intensity as a proxy). Cells were imaged using confocal microscopy. Using FIJI for image analysis, the Xi was segmented using H3K27me3 signal, and maximum intensity projections were generated from the multicolor z-stacks. Analysis was performed by measuring the DAPI sum intensity for the segmented Xi and the nucleus. Then, we divided the sum intensity of the DAPI-Xi area by the total DAPI sum intensity of the nuclear area. Values of the relative Xi area for each cell line were plotted using RStudio.

*MacroH2A-Mcm2 interaction (PLA) and Mcm2/ORC1/Cdc6/Cdt1 loading curves*. Proximity ligation assay between macroH2A1 or macroH2A2 and Mcm2 was analyzed in confocal images using FIJI. Replicating cells in any S-phase substage (EdU positive) were selected. First, maximum intensity projections were generated from multicolor Z-stacks, and nuclei were segmented using the DAPI signal. In this step, we obtained nuclear ROIs which were applied to the channel for PLA signal as selection. This removed the background outside the nuclei and the spots corresponding to additional cells in the image. Once the nuclear selection is applied to the ‘PLA channel’, we found the local maxima that correspond with the fluorescent PLA spots, maintaining the same value of ‘Prominence’ for all samples. In all our data sets, this value was set to >40, and for accurate counting, the function ‘Exclude edge maxima’ was used. The final output of this quantification was the number of maxima/spots per nucleus. Secondly, the density of spots per nuclear DNA/area and within the Xi was calculated. For this purpose, the number of nuclear spots was quantified using the method just described and divided by the total sum intensity in the area of the ROI (first, nuclear area). The same was repeated in each cell using a second ROI corresponding to the replicating Xi, giving us the density of local maxima or spots per DNA amount within the area of the inactive X chromosome. Finally, values of spot density (in the nucleus or the Xi) were normalized with the average value of nuclear spot density for control (Scramble) cells. With this normalization, the relative spot density and the fold increase within the Xi are calculated. Additionally, to quantify macroH2A2-Mcm2 interaction and to compare it with macroH2A1-Mcm2, high-content screening microscopy using Operetta was performed. Fluorescence PLA spots within the nucleus were quantified for the full cell population with the Harmony software (Version 3.5.1, PerkinElmer, UK) for control C2C12 and MEF cells and plotted with RStudio.

Mcm2/ORC1/Cdc6/Cdt1 loading coefficients and G1 loading curves were calculated in confocal images acquired using the UltraVIEW VoX spinning disc system and the confocal Leica TCS SP5II laser scanning microscope described before. FIJI was used for image analysis. First, maximum-intensity Z-projections were generated for the multicolor Z-stacks. For nuclear Mcm2/ORC1/Cdc6/Cdt1 loading coefficients, an ROI was generated for each cell using DAPI signal and including the full nucleus. Mcm2/ORC1/Cdc6/Cdt1 sum intensity and DAPI sum intensity were measured within the ROI, and Mcm2/ORC1/Cdc6/Cdt1 sum intensity was normalized and divided by DAPI sum intensity. For Xi Mcm2/ORC1/Cdc6/Cdt1 loading coefficients, two different ROIs were generated: Xi ROI1, within the Xi chromosome recognized by H3K27me3 accumulation, and control ROI2, an area of the same size as ROI1 but localized in a nuclear region outside the Xi and excluding chromocenters (highly condensed constitutive heterochromatic regions). Mcm2/ORC1/Cdc6/Cdt1 sum intensity in ROI1 was normalized by its corresponding DAPI sum intensity, and the same normalization was performed for sum intensity values in ROI2. Finally, the normalized Mcm2/ORC1/Cdc6/Cdt1 sum intensity in the Xi (ROI1) was divided by the normalized Mcm2/ORC1/Cdc6/Cdt1 sum intensity outside the Xi (ROI2), obtaining the final Xi loading coefficient for each cell. All the different loading coefficients were normalized by the average value of the control (Scramble cells) in the first time point. Afterward, normalized loading coefficients were plotted for each time point (from t1 to t8) obtaining Mcm2/ORC1/Cdc6/Cdt1 loading curve (Xi or full nucleus).

### Genome-wide and X chromosome macroH2A1/2 enrichment

The ChIP-seq datasets (as indicated in Supplementary Table 7) were downloaded from the GEO-database (Gene Expression Omnibus, https://www.ncbi.nlm.nih.gov/geo/) using sratoolkit (version 2.11.0). The quality of samples (dataset GSE142082) was evaluated using the FastQC program (version 0.11.9). When necessary, the reads of poor quality were trimmed using Trimmomatic (version 0.36), and the quality of the reads was checked again using FastQC. Subsequently, the trimmed reads were aligned to the mouse genome (mm10 genome assembly, https://hgdownload.cse.ucsc.edu/goldenpath/mm10/bigZips/) using bowtie2 (version 1.3.1), (parameters: –very-sensitive, –end-to-end). Samtools (version 1.10) was used to generate bam files. The regions enriched with macroH2A1/2 were identified using macs2 (version 2.1.2) (as broad peaks, option –broad –nomodel –extsize 150 -p 1.00e-5). Bedtools (version 2.29.2) were used to identify regions reproducibly found enriched with macro histone variants. For datasets GSE215884 and GSE40813 the genomic intervals corresponding to sites enriched with macro histone variants were downloaded from GEO repository. The genomic coordinates were converted from the genome assembly mm9 to mm10 (GSE40813) using the UCSC LiftOver tool for Linux (https://hgdownload.cse.ucsc.edu/admin/exe/linux.x86_64/liftOver). The density of macroH2A sites over autosomes was calculated as a number of ChIP-seq peaks of macroH2A per Mb for each chromosome. The density was normalized to the total number of peaks identified in all datasets. Plots were visualized using RStudio (Version 2023.03.1 + 446).

### Structural modeling with AlphaFold-Multimer

A local installation of AlphaFold-Multimer 2.3.2 was used to perform all structural modeling with AMBER relaxation (71). One prediction was generated per model and the prediction with the highest model confidence (0.8 × ipTM + 0.2 × pTM) was used for further analysis. Protein fragments were designed using available monomeric structural models from AlphaFold as provided by the AlphaFold database (72). Protein sequences were extracted from UniProt (73). Data analysis and plotting was done with Python and the pandas, numpy and matplotlib packages. Protein structure images were generated with PyMOL (74).

### Statistics and reproducibility

Data visualization and statistical analysis were performed using RStudio ((versions V1.2.5033 and V2023.03.1-446), https://rstudio.com/) and Microsoft® Excel® for Mac 2011 (Version 14.7.7) unless stated otherwise. Barplots show the average value of the distribution and the whiskers represent the standard deviation with a 95% confidence interval. Bar and line plots show normalized averaged values, and error bars show the respective standard deviation. Line intensity profile plots represent the fluorescence intensities along the distance of the selected arrow segment, normalized with the maximum value. In all figures showing boxplots, the box represents 50% of the data, starting in the first quartile (25%) and ending in the third (75%). The line inside represents the median. The whiskers represent the upper and lower quartiles. In most of the plots, outliers are excluded and defined as 1.5 times the interquartile range. The violin plots depict the density curves of the numeric data. The width of each curve corresponds with the approximate frequency of data points in each region. In the middle of each density curve is a small box plot, with the rectangle showing the ends of the first and third quartiles and the central dot the median. For the statistics, an independent two-group comparison was made for all conditions with Wilcoxon-Mann-Whitney or One-Way ANOVA tests. Related to this, n.s., not significant, is given for *P-*values ≥0.05; one star (*) is given for *P-*values <0.05 and ≥0.005; two stars (**) is given for values <0.005 and ≥0.0005; three stars (***) is given for values <0.0005; between the top of two boxes subjected to comparison. All statistical values (number (#) of cells (N), mean, median, standard deviation (SD), standard error of the mean (SEM), 95% confidence interval (CI), and *P-*values are summarized in Supplementary Table 8. All the software used for visualization and analysis is shown in Supplementary Table 9. The cells analyzed showed the reported behavior of the representative images selected.

## Results

### MacroH2A2 depletion reduces the duration of Xi replication without affecting S-phase length or overall cell cycle progression

To study the role of macroH2A in the synchronous replication dynamics of the Xi, we used primary female dermal fibroblasts from WT, macroH2A1- and macroH2A2-deficient newborn mice (Gaspar-Maia *et al.*, 2013) and created stable knockdown cell lines of macroH2A1 and macroH2A2 from mouse C2C12 myoblasts (75) (Supplementary Figure S1A). We confirmed the efficient knockdown (KD) of macroH2A1 and macroH2A2 by reverse transcription qPCR, yielding strongly decreased levels of the respective mRNA when compared to the scramble shRNA expressing C2C12 (Supplementary Figure S1B). The decrease of macroH2A1 or macroH2A2 was also confirmed at the protein level by Western blot analysis (Supplementary Figure S1C). Additionally, *in situ* immunofluorescence analysis was performed for macroH2A1. The quantification of macroH2A1 levels using high-content microscopy validated macroH2A1 knockdown specifically and had no effects on macroH2A2 levels (Supplementary Figure S1D). Confocal microscopy images of these immunofluorescence images illustrate the normal macroH2A1 pattern in control cells (Scramble shRNA) and its enrichment in the Xi (Supplementary Figure S1E). Quantification of macroH2A1 levels in knockdown cells was also performed using a different antibody against macroH2A1 and confocal microscopy imaging, obtaining similar results. This analysis and representative images of the immunofluorescence are shown in Supplementary Figure S1F.

To test for possible effects of macroH2A depletion on chromatin composition, we quantified the percentage of cells with H3K27me3 accumulation on the Barr body (Supplementary Figure S2A-B). Next, we quantified the nuclear and Xi-specific levels of H3K27me3 (Supplementary Figure S2C). H3K27me3 could be easily observed on the Xi by immunofluorescence as a bright nuclear cluster often localized in the nuclear periphery (76). After quantification, we did not find significant differences in the levels of this histone modification as a consequence of macroH2A depletion, showing that macroH2A depletion does not reverse H3K27me3 enrichment on the Xi. This is in accordance with previous studies showing that macroH2A1/2 KO cells maintain proper genomic localization of H3K27me3 (13,44). To further test whether depletion of macroH2A led to genomic instability and loss of Xi, we quantified the number of Xi clusters, using H3K27me3 as Xi marker. In the absence of macroH2A1.2, macroH2A1.1 plays a role in alternative end joining, which relates to Xi anaphase defects, genomic aberrations, and Xi loss (77). However, the inactivation of both splicing variants (which is the condition in our study) did not cause genomic instability in the Xi. Accordingly, there were no significant differences in Xi copy number between control and macroH2A knockdown cell lines. This corresponds to two Xi copies in 94.9–95.3% of the cells showing H3K27me3 accumulation in the Xi (Supplementary Figure S2D). The presence of two inactive X is due to the karyotype of the C2C12 cell lines, with quasi tetraploid karyotype (29). We also measured the DNA amount (using the total DAPI intensity) in the Xi, using H3K27me3 signal for segmentation, and in the full nucleus. This quantification showed no differences between control (Scramble) and macroH2A knockdown cell lines (Supplementary Figure S2E) and, therefore, we used H3K27me3 signal for an accurate segmentation of the Xi in subsequent experiments. In addition to H3K27me3, we quantified histone acetylation levels, which are normally depleted from the Xi territory (78,79). H3K9 and H4K8 acetylation showed no significant differences between control and macroH2A depleted cells, neither in the whole nucleus nor in the Xi (Supplementary Figure S2F). In our earlier work (29), we found that histone acetylation level on the Xi played a role in the regulation of its DNA replication dynamics, and depletion of histone H3K27me3 impacted the acetylation level. Hence, it was important to establish that macroH2A depletion did not affect either of these histone marks.

Next, we studied the cell cycle and the replication profiles in these cell lines in more detail. For this purpose, proliferating cell cultures of macroH2A knockdowns and knockouts were incubated with a cell-permeable thymidine analog, 5-ethynyl-2′-deoxyuridine (EdU), to label replicating DNA. Neither macroH2A1 nor macroH2A2 depletion affected the population doubling time or total S-phase duration (Figure 1B). Then, the frequency of the Xi replication pattern was calculated from microscopy images by counting the number of cells replicating the Xi from the total of EdU-positive S-phase cells. Interestingly, in macroH2A2-deficient cells the frequency of the Xi replication pattern was significantly reduced from the total fraction of S-phase cells (Figure 1C). A scheme of the different S-phase substages (early, mid, and late) (80–84) is shown in Figure 1D. Specifically, the Xi replication pattern occurs during mid-S-phase, and can be distinguished from early and late-S-phase using EdU distribution together with H3K27me3 signal (Figure 1E). The significant reduction in the number of cells replicating the inactive X chromosome strongly suggests that macroH2A2 plays a role in defining the Xi replication dynamics. And, in addition, these effects on Xi replication are independent of H3K27m3 and histone acetylation levels (Supplementary Figure S2).

To assess whether the lower frequency of the Xi replication pattern resulted from the loss of replication synchrony or rather from an even faster replication of the Xi, we followed DNA replication of the Xi over time in live cells. To this end, we transiently transfected the shRNA stably expressing cell lines with mRFP-PCNA, as a marker for active sites of DNA replication (60). Additionally to mRFP-PCNA, cells were co-transfected with MaSat-GFP, a polydactyl zinc finger protein that specifically binds to mouse chromocenters (61,85) to exclude any confusion between the Xi pattern and the late replicating chromocenters (85). Double transfected cells were live-imaged over time, and the appearance and persistence of the Xi synchronous pattern were quantified (Figure 1F and Supplementary Movies S1–Supplementary Movies S3). This analysis demonstrated that in all three cell lines, 100% of the cells undergoing mid-S-phase exhibited a synchronous Xi pattern (Figure 1F-left). However, while in control and macroH2A1 knockdown cells, the pattern persisted for an average of approximately 80 min (4 frames), in macroH2A2 knockdown cells the pattern disappeared after an average of 52 min (2–3 frames). These results clearly showed that in macroH2A2 deficient cells, the replication of the Xi still takes place synchronously, yet it happens faster than in wild-type cells or macroH2A1 deficient cells. Importantly, the live cell analysis confirmed that both knockdown cell lines exhibited no changes in the total S-phase duration, pointing to a specific effect of macroH2A on the Xi replication dynamics.

### MacroH2A depletion increases Xi replication progression rate

**Figure 2.**
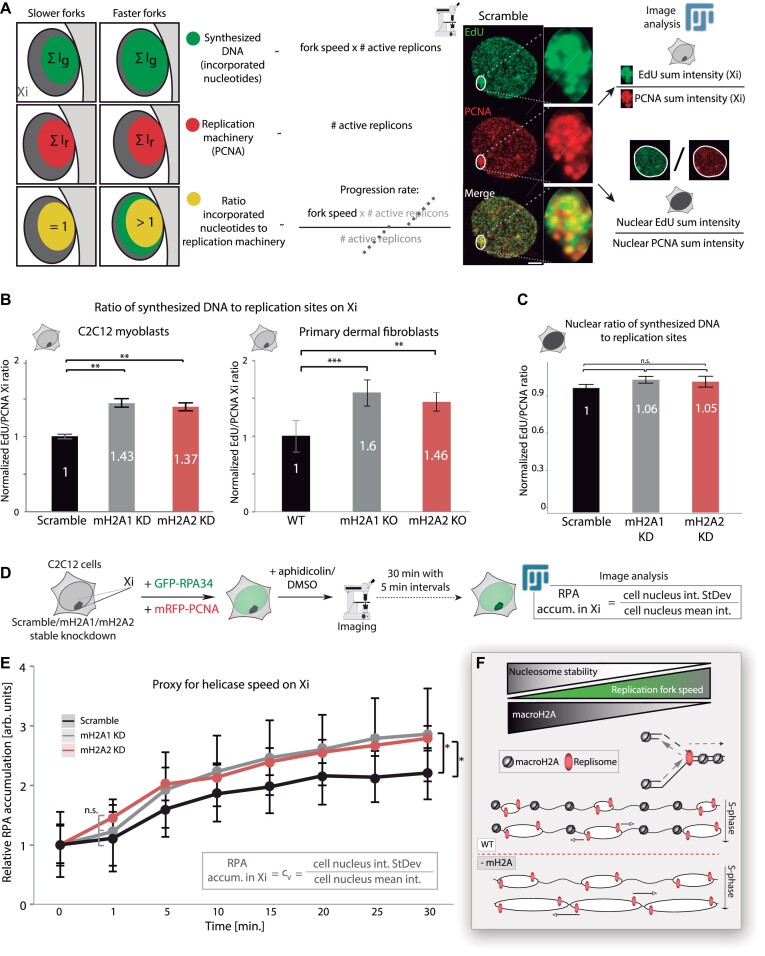
MacroH2A1 and macroH2A2 depletion increases replication fork speed. (**A**) Scheme showing the fundamentals and the pipeline of the analysis. DNA synthesis speed was measured by calculating the ratio of the total nucleotide signal incorporated on the Xi during a 20-min labeling pulse (as a proxy for the amount of DNA synthesized during the indicated period) to the total Xi PCNA signal (as a marker for the number of replisomes). Representative images and image analysis procedures are shown. (**B**) Barplots showing the average values of the ratio EdU/PCNA within the Xi for stable knockdowns and knockout cells. N-numbers (cells): Scramble 174, mH2A1 KD 150, mH2A2 KD 174, eight independent replicates; WT 22, mH2A1 KO 32, mH2A2 KO 26, two independent replicates. (**C**) Barplots showing the average values of EdU/PCNA ratios for the full nucleus. In both (B) and (C), the values of EdU/PCNA ratios were normalized to the average of control cells (Scramble or WT). N-numbers (cells): Scramble 28, mH2A1 KD 23, mH2A2 KD 26, two independent replicates. (**D**) Scheme and pipeline of the experiment: as a proxy for helicase activity was analyzed by measuring single-stranded binding protein (GFP-RPA) accumulation on the replicating Xi. Cells were double-transfected as indicated 24 h before imaging. RPA accumulation at replicating Xi was calculated as depicted in the formula and normalized to the average of the respective pretreatment control. (**E**) Line Plots corresponding to the analysis of (D), showing the mean values for RPA accumulation (Cv ± standard deviation in the whiskers). Cells were imaged as depicted. N-numbers (live-cells): Scramble 18, mH2A1 KD 11, mH2A2 KD 12, two independent replicates. (**F**) A diagram represents the main output of these experiments: macroH2A depletion is directly associated with an increase in replication fork speed, which relates to the higher stability of macroH2A-containing nucleosomes. Barplots show the average value of the distribution and the whiskers represent the standard error with a 95% confidence interval. Statistical significance was tested with a paired two-sample Wilcoxon test (n.s., not significant, is given for *P-*values ≥ 0.05; one star (*) for *P-*values <0.05 and ≥0.005; two stars (**) is given for values <0.005 and ≥0.0005; three stars (***) is given for values <0.0005). N-numbers and *P-*values are shown in Supplementary Table 8 (Statistics). Scale bars = 5 μm.

Next, to investigate whether the faster Xi replication observed in macroH2A2 stable knockdown cells resulted from a faster replication fork progression, we co-stained the replisome marker PCNA and modified nucleotides (EdU) incorporated into the replicating Xi. This allows us to quantify potential changes in the replication progression rate on the Xi and to compare with global nuclear values: while PCNA is part of the DNA replication machinery and is therefore proportional to the number of active replisomes but independent of the replication fork speed, the amount of incorporated nucleotides is proportional to both the number of active replisomes and the replication fork speed. By calculating the ratio of the total nucleotide signal to the total PCNA signal on the Xi and comparing the knockdown/knockout cell lines to their respective controls, we were able to assess changes in replication progression rate (Figure 2A). Unexpectedly, all macroH2A-depleted cell lines showed a prominent increase of the ratio of nucleotides incorporated on the Xi to the PCNA signal, indicating that in both cases, replication fork progression in the Xi is enhanced (Figure 2B). However, there were no significant changes in global EdU/PCNA ratios for the full nucleus, thus, no increase in global replication fork speed (Figure 2C). The later observation matched our previous results showing no changes in total S-phase duration (Figure 1B and 1F). Therefore, we can conclude that both macroH2A1 and macroH2A2 depletion increase DNA replication progression rate specifically in the inactive X chromosome. But, strikingly, only macroH2A2 depletion reduces the duration of Xi replication.

To further validate these findings, we used a system based on the decoupling of DNA synthesis from unwinding via a drug treatment (62,86). We treated the cells with aphidicolin which reversibly inhibits DNA polymerization without interfering with DNA helicase unwinding. Hence, the DNA polymerase gets uncoupled from the DNA helicase, leading to continuous DNA duplex unwinding, and the resulting single-stranded DNA (ssDNA) is continually covered with ssDNA binding protein (RPA). Additionally, the uncoupling of the replisome leads to the disassembly of proteins involved in DNA replication elongation (e.g. PCNA) from DNA replication sites. We used this system to study the helicase unwinding speed of the inactive X chromosome in stable macroH2A1 or macroH2A2 knockdown cells, by measuring the accumulation of RPA within the Xi. For this purpose, we transfected cells with mRFP-PCNA to identify S-phase cells and with GFP-RPA to determine the helicase unwinding speed via RPA accumulation over time (Figure 2D). In the stable knockdown cells, detection and analysis of the helicase activity and speed in replicating Xi showed a time-dependent accumulation of RPA at replication sites in cells treated with aphidicolin. However, macroH2A-depleted cells showed a significantly higher amount of RPA accumulation on the Xi than control cells (Figure 2E). Representative images of these time-lapse experiments are shown in Supplementary Figure S3A, B. Complete inhibition of the polymerase by aphidicolin was verified by PCNA dissociation from replication foci and by incubating the cells with BrdU after 30 min of aphidicolin treatment (Supplementary Figure S4). In this case, no BrdU incorporation was detected. Control cells treated with DMSO, neither showed RPA accumulation nor PCNA dissociation from the replisome, and incubation with BrdU showed replication foci pattern colocalizing with the RPA signal (Supplementary Figures S3B and S4).

We can, hence, conclude that both macroH2A isoforms slow down DNA helicase unwinding and DNA synthesis, likely due to the higher stability of macroH2A-containing nucleosomes. Therefore, their depletion increases Xi replication progression rate (Figure 2), showing the prominent role of macroH2A in Xi replication dynamics (Figure 2F). Nonetheless, this outcome seems paradoxical taking into consideration our previous result of no significant changes in Xi replication timing for macroH2A1 knockdown (Figure 1C–F). But, far from being contradictory, our findings point to a distinct and unique effect for macroH2A1 in Xi replication dynamics, likely in replication origin firing synchrony.

### MacroH2A1 promotes synchrony of replication origin firing in the Xi

**Figure 3.**
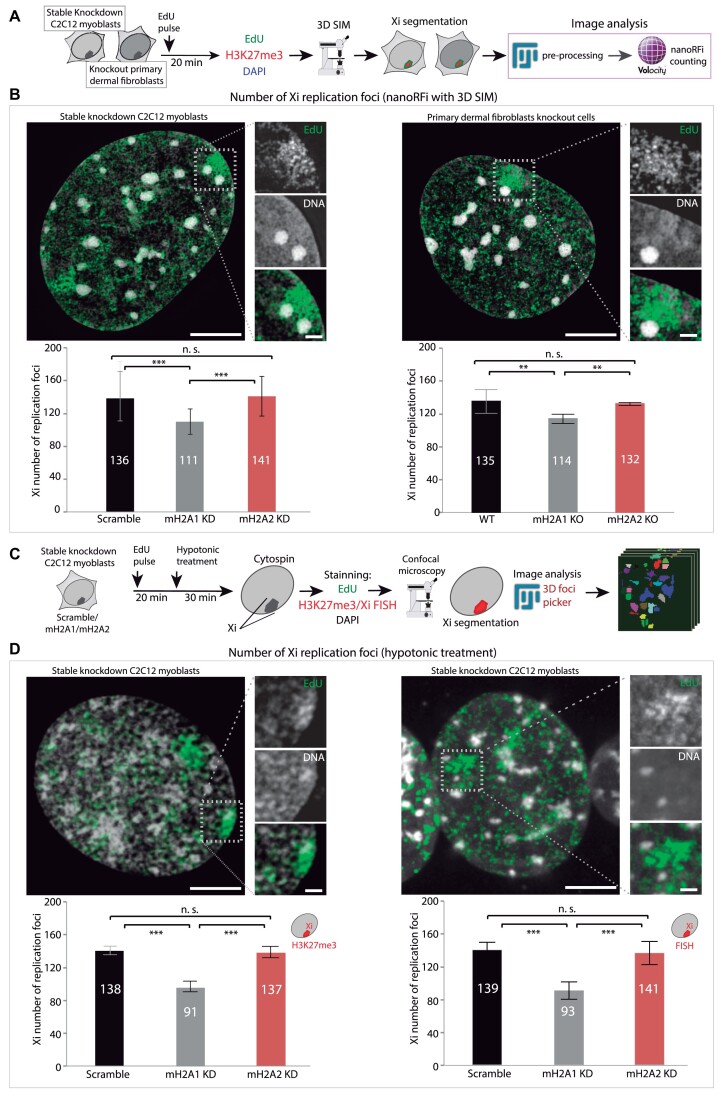
MacroH2A1 depletion reduces the number of active Xi replication origins. (**A**) Pipeline of the experiment and image analysis for nanoRFi using 3D-SIM. Briefly, cells were incubated with EdU for 20 min to label replicating DNA, fixed and stained for H3K27me3, EdU and DAPI for DNA counterstaining. Then, C2C12 stable knockdowns and primary dermal fibroblast knockouts were imaged using super-resolution microscopy (3D-SIM). Images were preprocessed using FIJI, H3K27me3 signal was used for Xi segmentation, and nanoRFi were counted using Volocity software. (**B**) Results of the image analysis described in (A) for C2C12 stable knockdowns (left) and primary dermal fibroblast knockouts (right). Representative 3D-SIM images of control cells are shown in each case (top), EdU (green), and DAPI (gray). Amplified regions (white boxes) show the nanoRFi on the Barr body (Xi). Below the images, barplots show the mean number of nanoRFi within the Xi quantified in super-resolution microscopy images in stable knockdowns (left) and knockouts (right) cell lines. *N*-numbers (cells)/replicates: Scramble 18/3, mH2A1 KD 25/3, mH2A2 KD 22/3, WT 4, mH2A1 KO 6, mH2A2 KO 10. (**C**) Pipeline of the experiment and image analysis for hypotonically resolved nanoRFi. Briefly, after EdU incubation as above, cells were hypotonically treated in KCl solution (75 mM) for 30 min and then cytospined. H3K27me3 or X-FISH was performed for Xi segmentation in C2C12 stable knockdown cells, followed by EdU detection and DNA counterstaining with DAPI. Confocal images were acquired, and FIJI was used for image preprocessing and counting of nanoRFi using the plugin 3D foci picker. (**D**) Results of the image analysis described in (C) using H3K27me3 for Xi segmentation (left) or X-FISH (right). Representative confocal images of control (Scramble) cells are shown in each case (top), EdU (green), and DAPI (gray). Amplified regions (white boxes) show the nanoRFi on the Barr body (Xi). Below the images, barplots show the mean number of hypotonically resolved nanoRFi within the Xi quantified in confocal microscopy images. *N*-number (cells)/replicates: Scramble 34/2, mH2A1 KD 30/2, mH2A2 KD 23/2 (H3K27me3); Scramble 14, mH2A1 KD 7, mH2A2 KD 8 (X-FISH). Barplots show the average value of the distribution and the whiskers represent the standard error with a 95% confidence interval. Statistical significance was tested with a paired two-sample Wilcoxon test (n.s., not significant, is given for *P-*values ≥0.05; one star (*) for *P-*values <0.05 and ≥0.005; two stars (**) is given for values <0.005 and ≥ 0.0005; three stars (***) is given for values <0.0005). *N*-numbers and *P-*values are shown in Supplementary Table 8 (statistics). Scale bars: 5 μm and 1 μm in the amplified region.

To gain more insight into the role of macroH2A1 in replication dynamics, we quantified the number of replication foci (RFi) within the Xi. Origin firing has an important function in replication synchrony. As it has been shown, Xi replication is highly synchronous (29). Therefore, we aimed to clarify whether the differences in Xi replication dynamics, or Xi replication rate, observed between macroH2A knockdown cells resulted from different numbers of replication origins being activated in parallel, from the faster replication fork progression, or a combination of both. For this purpose, we labeled replicating DNA by incubating the cells with EdU and imaged the replicating Xi after cell fixation using 3D structured illumination microscopy (3D-SIM) (66). A 3D rendering of C2C12 nuclei imaged with super-resolution microscopy is shown in Supplementary Movie S4. We have shown before that these correspond to individual replicon units originating from single-origin firing (64,87). We then quantified the number of nanoRFi on the Xi using EdU and H3K27me3 signal for Xi segmentation (Figure 3A). Surprisingly, macroH2A2-depleted cells showed no increase in the number of super-resolved nanoRFi in the Xi, while macroH2A1 knockdown and knockout cells exhibited a significant decrease in these numbers (Figure 3B), meaning a reduction in origin initiation frequency.

We validated our results from 3D-SIM using confocal microscopy of hypotonically resolved replication foci (54,64,87) (Figure 3C). The effects of the hypotonic treatment in nuclei volume (diameter, surface area, and flattening (z-axis)) are shown in Supplementary Figure S5A-B), with a significant increase in nuclear diameter and surface area, and the accompanying flattening by the reduction in z-axis for treated cells. Representative images of mid-S-phase cells with (w/) and without (w/o) hypotonic treatment are shown in Supplementary Figure S5C. Interestingly, average values of the standard deviation of the DNA dye DAPI also decreased after this treatment indicating chromatin decondensation in C2C12 control cells (Supplementary Figure S5D). To verify this effect, we performed a 3D quantitative analysis of chromatin structure. Using DAPI as a proxy, this analysis assesses different chromatin compaction levels from 1, less compacted, to 7, highly compacted cores of heterochromatin (Supplementary Figure S5E) (69,88). Indeed, we found that hypotonic treatment increases the fraction of the lowest compaction class indicating chromatin decondensation (Supplementary Figure S5F). Therefore, hypotonic treatment and cytospin are suitable tools to increase the resolution of nanoRFi and their accurate counting by confocal microscopy. Reproducing the trend of the previous results (Figure 3B) macroH2A1 knockdown cells showed a reduction in the number of Xi nanoRFi (Figure 3D), independently of the method used for Xi segmentation: H3K27me3 or X-FISH. Representative images for X-FISH are shown in Supplementary Figure S5G.

Importantly, these Xi nanoRFi measurements were reproduced using different microscopy techniques, segmentation methods, and image analysis approaches. Image analysis pipelines for nanoRFi (3D-SIM) versus hypotonic resolved nanoRFi are shown in Supplementary Figure S6. In summary, we obtained a mean value of 138/140 RFi in control (Scramble) and macroH2A2 knockdown versus only 95 RFi for macroH2A1 knockdown cells. As reported in the literature (89), in a scenario where modulation of nucleotide levels increase replication fork speed and this is accompanied by lower origin initiation frequency, we would have expected a lower number of nanoRFi. This is the case for macroH2A1 depletion but not for macroH2A2. Our results demonstrate that macroH2A1 depletion decreases the synchrony of Xi replication by reducing the number of simultaneously firing replication sites at any given time, and, on the other hand, macroH2A1 knockdown also speeds up replication fork progression (Figure 2). Consequently, the increased replication progression rate compensated for the reduced numbers of replication sites, explaining the unchanged Xi replication timing of 80 min relative to control (Figure 1F). For macroH2A2 depletion, we revealed a different scenario in which Xi replication is more than 35% shorter, but replication synchrony (number of Xi nanoRFi) is not affected. Although both macroH2A isoforms affect the replication progression rate, only macroH2A1 regulates the highly synchronic origin firing in the inactive X chromosome. Hence, macroH2A1-containing nucleosomes are not just mere obstacles for replication.

### MacroH2A1 regulates Xi replication schedule

**Figure 4.**
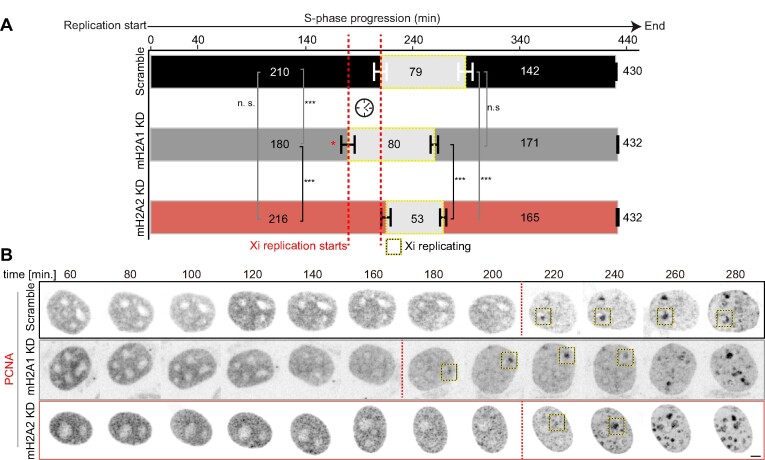
MacroH2A1 depletion switches the replication timing of the Xi to an earlier time in S-phase. (**A**) Living C2C12 cells expressing mRFP-PCNA (red) as a marker for sites of active DNA replication and MaSat-GFP (green) were imaged at 20-min intervals for several hours using a spinning disk confocal microscope. The start of replication, start, and duration of the Xi replication (light grey boxes highlighted with yellow square), and total replication times were quantified in minutes (number of frames*20 min) and plotted as a barplot. *N*-numbers (Live-cells): Scramble 33, mH2A1 KD 32, mH2A2 KD 27 (Xi starts); Scramble 16, mH2A1 KD 23, mH2A2 KD 25 (Xi replication time). Three independent replicates. Barplots show the average value of the distribution and the whiskers represent the standard error with a 95% confidence interval. Statistical significance was tested with a paired two-sample Wilcoxon test (n.s., not significant, is given for *P-*values ≥0.05; one star (*) for *P-*values <0.05 and ≥0.005; two stars (**) is given for values <0.005 and ≥0.0005; three stars (***) is given for values <0.0005). *P-*values are shown in Supplementary Table 8 (Statistics). The timing and appearance of the Xi synchronous replication pattern (yellow squares) can be visualized in the representative images in (**B**), showing the premature Xi replication in macroH2A1 knockdown cells. Dash red lines indicate the start of Xi replication for each condition. See also full Supplementary Movies S1–S3. Scale bars = 5 μm.

Next, we examined whether the reduction in the number of active origins and replication synchrony is paired with changes in the Xi replication schedule during S-phase. The highly coordinated and delayed replication of the inactive X chromosome is one of the features of transcriptionally silent chromatin, while its transcriptionally active counterpart starts replicating earlier and asynchronously throughout S-phase (29). Similarly, in *Xenopus* embryos the untranscribed genome is replicated extremely fast, which requires the synchronous firing of all licensed origins (90–92). To assess whether the reduction of Xi active origins in macroH2A1 depleted cells corresponds to changes in Xi replication schedule, we followed DNA replication in the Xi over time. Specifically, we measured the time in minutes from the start of the S-phase to the start of Xi replication. This analysis revealed that, for macroH2A1 knockdown cells, the onset of Xi replication is earlier than in control or macroH2A2 knockdown cells, on average one or two frames before (1 frame = 20 min) (Figure 4A), as is shown in the representative images in Figure 4B. Hence, macroH2A1 depletion affects both replication timing and synchrony of the inactive X chromosome.

The depletion of macroH2A isoforms showed a specific effect on the replication dynamic of the Xi, without affecting total S-phase duration (Figures 1B, F, and 4A) or global replication progression rate (Figure 2C). To account for this, we investigated the distribution of macroH2A isoforms genome-wide. We used publicly available ChIP-seq datasets for macroH2A isoforms: both female and male mouse embryonic fibroblast (MEF) and dermal fibroblast (DF) (Supplementary Table 7). In these data sets, we analyzed the density of macroH2A1/2 sites over the autosomes and chromosomes X and Y (Supplementary Figure S7A). Although the density of macroH2A2 distribution in the X appeared to be more variable and depending on the cell type analyzed, the density of macroH2A1/2 sites in the autosomes is comparable in all the cell lines analyzed independently whether they are male or female. In contrast, a higher density of macroH2A1/2 was observed on chromosome X in female cell lines in comparison to male cell lines. As in the female datasets signals from active and inactive chromosomes cannot be distinguished, the cumulative enrichment on both chromosomes was determined as an average of both (Supplementary Figure S7B). Overall, from the ChIP-seq analysis comparing male and female data sets, we infer a higher abundance of macroH2A isoforms in the inactive X chromosome. This is compatible with immunofluorescence staining of macroH2A1 (Supplementary Figure S1E), showing its enrichment in the inactive X chromosome and explaining the predominantly local effects of macroH2A depletion on Xi replication dynamics.

In summary, the enrichment of macroH2A isoforms in the Xi regulates its synchronous replication dynamics. Hence, we next addressed how macroH2A1 regulates the synchrony of origin firing.

### MacroH2A1 regulates chromatin loop size and corresponding numbers of replicon units

**Figure 5.**
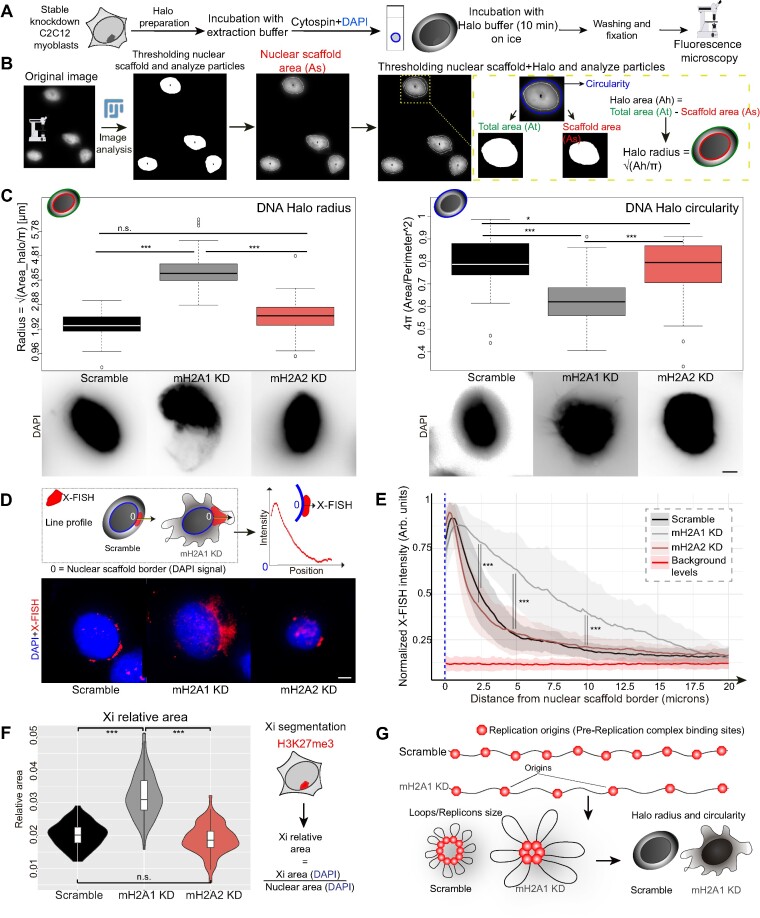
MacroH2A1-depleted cells show wider and irregular DNA Halos, corresponding to an increase in the size of chromatin loops and replicon units. (**A**) Pipeline of the experimental procedure. Cells were incubated with high-salt extraction buffers and cytospined onto coverslips. Afterward, nuclear DNA (Scaffold) and DNA in the Halos were stained with DAPI, followed by incubation with Halo buffer, washing buffers, and fixation. After imaging the cells using fluorescence microscopy, the image analysis was performed as depicted in (**B**) Nuclear scaffold and total area (Nuclear scaffold + Halo) were both thresholded, and their areas and circularity were measured. Areas were subtracted (At-As) and then the Halo radius was calculated. Results of these analyses are shown in (**C**) as boxplots: increase in the Halo radius area for macroH2A1 depleted cells (left), and decrease in circularity compared with control (right). Representative images for each condition are shown below the boxplots. N-numbers (cells): Scramble 56, mH2A1 KD 60, mH2A2 KD 61, three independent replicates. (**D**) X-FISH image analysis scheme performed by applying line-profile analysis in FIJI. Briefly, X-FISH fluorescence intensities were measured in a line drawn from the nuclear scaffold border (0, blue oval), up to 20 microns over the X chromosome cloud (red). Representative images are shown below the scheme. (**E**) Line plots showing the results of the quantification explained in (D). Line plots show the average normalized X-FISH intensities at each point/pixel of the line, and their position (in microns) relative to the nuclear scaffold. The error bands show the respective standard deviation. 95% confidence intervals are indicated in the plot as a band. N-numbers (cells): Scramble 81, mH2A1 KD 82, mH2A2 KD 75, two independent replicates. (**F**) Violin plots showing values of Xi relative area, with a significant increase for macroH2A1 knockdown cells prompting Xi decondensation (**G**) Scheme representing the relationship between DNA Halos radius and chromatin loops and the role of macroH2A1: its involvement in chromatin loops formation determines the space between replication origins (interorigin distances) affecting the number of replication origins within the Xi. For all boxplots, the box represents 50% of the data, starting in the first quartile (25%) and ending in the third (75%). The line inside represents the median. The whiskers represent the upper and lower quartiles. The violin plot depicts the density curves of the numeric data. Statistical significance was tested with a paired two-sample Wilcoxon test (n.s., not significant, is given for *P-*values ≥0.05; one star (*) for *P-*values < 0.05 and ≥ 0.005; two stars (**) is given for values <0.005 and ≥0.0005; three stars (***) is given for values <0.0005). *N*-numbers and *P-*values are shown in Supplementary Table 8 (statistics). Scale bars: 5 μm.

Previous studies have established a direct relationship between chromatin loops, one level of higher-order organization of chromatin fibers, and the organization of DNA replication (55,89). Beyond that, replicon size is determined by the spacing between active origins, which is correlated with the length of chromatin loops (93). Therefore, we next investigated the effect of macroH2A depletion on chromatin loop structure using the DNA Halo technique. This technique detects changes in chromatin organization at the level of DNA loops by loop extrusion after histone extraction by high-salt incubation (56,57). The extracted loops can be distinguished from a densely stained central chromatin core or nuclear scaffold, providing a measure of their size. Longer loops would correspond with longer interorigin distances or longer replicon sizes. Using C2C12 stable knockdown cells, we performed the DNA Halo approach using the protocol from (70), followed by fluorescence microscopy (Figure 5A). Then, we performed image analysis to measure DNA Halo radius and circularity (Figure 5B). The DNA Halo radius of macroH2A1 depleted cells was significantly larger (more than 2-fold increase) than control or macroH2A2 knockdown cells (Figure 5C-left). In addition, DNA Halos of macroH2A1 knockdown cells were not only larger, indicating larger interorigin distances, but also visibly more irregular, which was measured by a decrease in their circularity (Figure 5C-right). A gallery of images illustrating these phenotypes is shown in Supplementary Figure S8A.

Then, we performed X-FISH after DNA Halo preparation to investigate whether larger DNA Halos in macroH2A1 depleted cells were directly related to bigger loops in the X(i) chromosome (Figure 5D, E). We determined the spatial distribution of the X chromosome within the Halos, measuring the intensity of the FISH signal from the border of the nuclear scaffold (0) up to 20 micrometers outside the scaffold border (Figure 5D). Representative images are shown in Figure 5D and Supplementary Figure S8B. In small Halos seen in control and macroH2A2 depleted cells, X signals were similarly distributed in a short region from 0 to 2.5 microns closer to the nuclear scaffold border. On the other hand, in large Halos obtained for macroH2A1 depleted cells, X signals were distributed mostly in the DNA Halos, significantly further from the nuclear scaffold (from 1 to 17.5 microns) (Figure 5E). Thus, macroH2A1 depletion specifically affects DNA loops size in the X chromosome.

We also investigated Xi chromatin structure changes in non-extracted cells. Strikingly, the relative area of the Xi was significantly increased for macroH2A1 knockdown cells (Figure 5F), prompting Xi decondensation and showing the effect of this histone variant on Xi chromatin structure. We also found that non-extracted cells showed a reduction of nuclear roundness (Supplementary Figure S8C), in line with previous findings reporting the specific association of macroH2A1 with the nuclear lamina and its role in chromatin architecture (7,94). In light of these results, we propose a model in which macroH2A1 depletion limits the number of active origins by increasing the length of chromatin loops that correspond with replicon units. Thus, interorigin DNA regions are looping out in rosette-like structures (Figure 5G).

**Figure 6.**
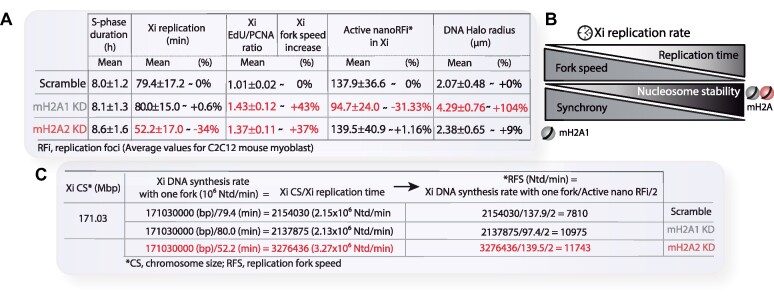
Counterbalance between active origins and replication progression rate. (**A**) Summary of the mean values obtained during the analysis of Xi replication dynamics on C2C12 stable knockdown cells (S-phase duration, Xi replication duration, Xi replication fork speed, number of nanoRFi, and DNA Halo radius). Relevant changes compared with control (Scramble cells) are highlighted in red. (**B**) A diagram illustrating the trend of these results is shown: nucleosome stability is anti-correlated with replication fork speed, while macroH2A1 is correlated with replication synchrony. Synchrony and replication fork speed affect Xi′s replication rate. (**C**) Table summarizing the estimated replication fork speed in control versus macroH2A depleted cells taking into consideration: the size of the X chromosome, the duration of Xi replication, and in a subsequent step the number of active origins and their bi-directionality.

The limitation in the number of active origins could slow down Xi replication duration but is counterbalanced by the increase in replication progression rate, also one consequence of macroH2A1 depletion (see Figure 2). The counterbalance between active origins and replication progression rate is dissected in Figure 6, which summarizes the different S-phase measurements, including the total average nanoRFi numbers for macroH2A knockdown C2C12 cells, with significant changes highlighted in red. Briefly, Xi replication time is approximately 34% shorter only for macroH2A2 depleted cells, however, Xi progression rate is 43% and 37% faster for macroH2A1 and macroH2A2 depleted cells, respectively. Regarding the number of active origins, only macroH2A1 depletion showed a decrease of 31%, which is paired with a two-fold increase in DNA Halo/loop radius. On average, 138/140 origins are active in the Xi at any given time for control and macroH2A2 depletion, respectively, versus 95 active origins for macroH2A1 knockdown cells. These numbers of origins are active at any given time during Xi replication and are likely activated in two or three consecutive waves. Knowing the size of the X chromosome in mouse cells (171.03 Mb) (95,96), and Xi replication duration (Figure 6A, see Figure 1), we can estimate the average DNA synthesis rate in nucleotides per min in the Xi. The latter is higher for macroH2A2 depletion in agreement with our experimental data on replication progression rate measured as the ratio of incorporated nucleotides to replisomes (see Figure 2), and shorter Xi replication duration. For macroH2A1 depletion, the reduction in the number of active origins compensates for the increase in replication progression rate yielding the same total Xi replication duration as control cells (Figure 6B). From the number of active origins at any given time during Xi replication, we can estimate the replication fork speed (RFS, Ntd/min) in the inactive X chromosome (Figure 6C). Interestingly, Xi estimated RFS (7.8 × 10^3^ nucleotides/min) is three-fold higher than the average genomic RFS (2.46 × 10^3^ nucleotides/min) for C2C12 mouse cells reported previously (64). This is consistent with previous studies showing that the bulk of Xi replication is completed approximately twice as fast as any other chromosome, and almost three times faster than the bulk of Xa replication (31). An alternative explanation would be that it takes two to three waves of origin activation to replicate the Xi, which means that instead of 138 active origins in total there would be two to three times that number with consequently two to three times lower replication fork speed. In such a situation, the replication fork speed in the Xi would be similar to the rest of the genome. Altogether, our data indicate the prominent role of both macroH2A isoforms in the Xi replication dynamics, with macroH2A1 modulating the number of active origins and opening the question of how this is brought about.

### MacroH2A1 interacts with Mcm mostly within the Xi

We have described the novel role of macroH2A1 in Xi replication synchrony determining the size of chromatin loops, which, in turn, has been shown to control the choice of replication initiation sites (89). In mammalian cells, there is a window of time in the G1 phase during which the assembly of pre-replication complexes (pre-RCs) into origins occurs, and the replication timing program is established (97,98). To assess whether macroH2A1 affects origin licensing, we focused on the loading of the DNA helicase complex (minichromosome maintenance (MCM) proteins) onto chromatin after the exit from mitosis (99). During the G1 phase, the origins of replication are licensed by the recruitment and loading of proteins that form the pre-replicative complex, such as ORC (Origin recognition complex) and Mcm (100). Their activation will form replication clusters during S-phase (101), with neighboring origins clustering together and interorigin regions looping out (102). Previous studies demonstrated the role of cohesin stabilizing chromatin loops and interacting with Mcm proteins at replication origins, which affects S-phase progression (55). In this context, we addressed whether nucleosome composition can regulate the access to replication origins.

**Figure 7.**
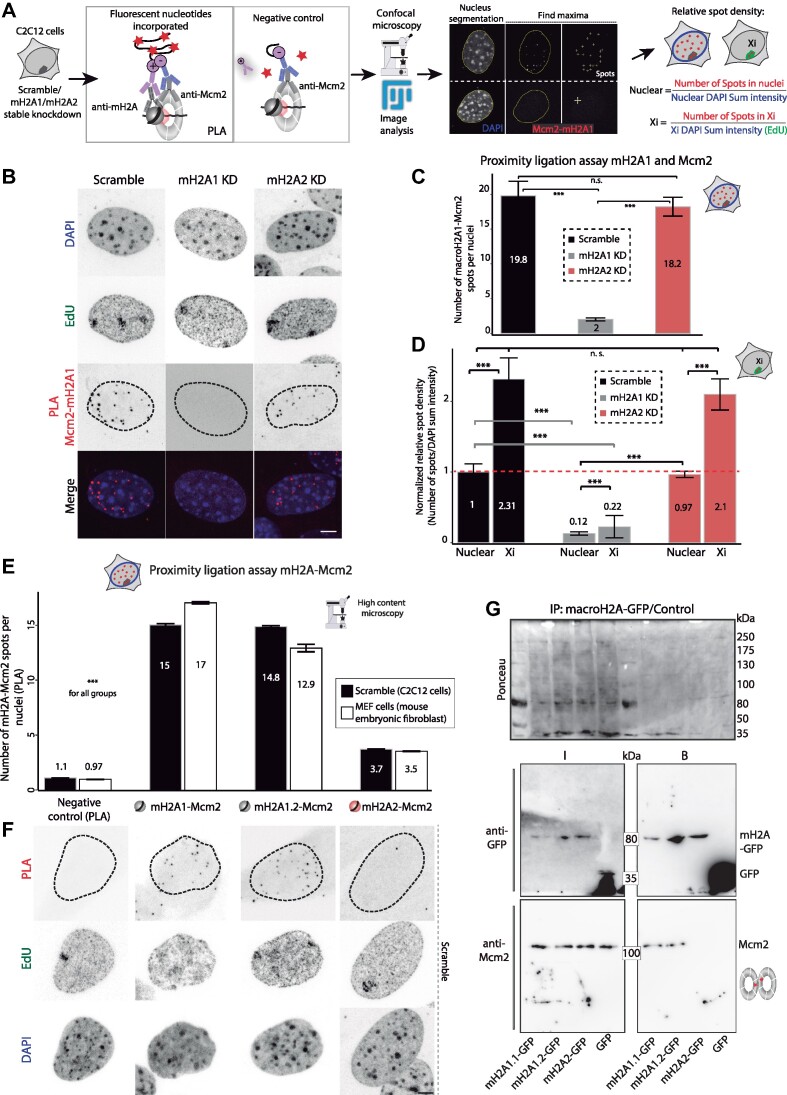
MacroH2A1.1 and macroH2A1.2, but not macroH2A2, interact with the DNA helicase. (**A**) Diagram illustrating the fundamentals of the proximity ligation assay (PLA) to analyze the *in situ* interaction between macroH2A1 and Mcm2, including a pipeline of image analysis. In this technique, small oligonucleotide probes, (+) and (–), conjugated to secondary antibodies specifically recognize the primary antibodies against the proteins of interest. When the two probes are closer than 40 nm, ligation by ligase incubation can occur. This generates circular DNAs that will be amplified by a polymerase incorporating fluorescently labeled nucleotides. Afterward, fluorescent spots can be detected and quantified using microscopy and image analysis, considering each spot an interaction site between the two proteins. The number of spots and their location, the Xi in this case, can be quantified by image analysis using FIJI. Negative control was performed using only one of the primary antibodies. (**B**) Representative confocal images of the PLA assay between macroH2A1 and Mcm2, showing spots corresponding to protein-protein interaction for control and macroH2A2 knockdown cell lines. (**C**) Barplots showing mean values for the number of PLA spots per nuclei in knockdown cell lines after performing the image analysis described in (A). N-numbers (cells): Scramble 27, mH2A1 KD 34, mH2A2 KD 33, two independent replicates. (**D**) In addition to the previous analysis, the density of spots was calculated for the full nuclei and the Xi, dividing the number of spots counted in their respective areas by values of the sum intensity of DAPI (as a proxy for DNA content). In this case, all mean values were normalized by the average value of nuclear density for control (Scramble) cells. Barplots show these quantifications, with a significantly higher density of spots for the Xi. N-numbers (cells): Scramble 21, mH2A1 KD 23, mH2A2 KD 23, two independent replicates. (**E**) The same experimental pipeline of (C) was used to analyze the interaction between macroH2A1.2, macroH2A2, and Mcm2. In this case, high-content screening microscopy was used for imaging, and quantification of spots was performed using the Harmony software. The results of these analyses are shown in the barplot, where macroH2A1.2/macroH2A2-Mcm2 interaction is compared with the interaction macroH2A1-Mcm2 analyzed before. In addition, macroH2A-Mcm2 interaction is compared with the negative control of the assay and with a different cell line, MEFs. *N*-numbers (cells)/replicates: 7356/2, 3980/1, 11 707/2, 2816/1, 4265/2, 4762/2, 11 617/2, 6710/1. (**F**) Representative confocal images of the PLA assay on (**E**), showing spots corresponding to protein-protein interaction for macroH2A1/macroH2A1.2-Mcm2. (**G**) Co-immunoprecipitation: C2C12 cells were transfected with EGFP or EGFP-tagged macroH2A1.1, macroH2A1.2, or macroH2A2. Cell extracts were analyzed by immunoprecipitation with immobilized GFP-binding nanobody, followed by detection with antibodies against GFP, Mcm2, Mcm4 and Mcm5 (Supplementary Figure S9A). The cut-outs show input/bound GFP and input/bound Mcm fractions. To the right, the scheme of the DNA helicase shows the two hexamers. The position of Mcm2 subunits on the Mcm hexamers is indicated with a red star. Two additional replicates for these co-immunoprecipitations are shown in Supplementary Figure S9B-C. Barplots show the average value of the distribution and the whiskers represent the standard error with a 95% confidence interval. Statistical significance was tested with a paired two-sample Wilcoxon test (n.s., not significant, is given for *P-*values ≥0.05; one star (*) for *P-*values <0.05 and ≥0.005; two stars (**) is given for values <0.005 and ≥0.0005; three stars (***) is given for values < 0.0005). *N*-numbers and *P-*values are shown in Supplementary Table 8 (statistics). Scale bars: 5 μm.

First, we investigated the potential interaction of macroH2A1 with the Mcm helicase complex, specifically with the subunit Mcm2. For this aim, we performed proximity ligation assay (PLA) experiments (58,103) to test *in situ* protein-protein interaction between macroH2A1 and Mcm2 using the knockdown cell lines. See the explanation of this technique and subsequent image analysis in Figure 7A. All the samples were extracted before fixation and staining to remove soluble Mcm2 (50). The PLA assay showed many nuclear spots for control (Scramble) and macroH2A2 depleted cells, but almost no spots for macroH2A1 knockdown (Figure 7B). We quantified the total number of spots globally, within the nucleus, finding an average of 19.8 and 18.2 spots for control and macroH2A2 depleted cells respectively, while an average of only 2 spots per nucleus was found for macroH2A1 knockdown cells (Figure 7C), similar to the negative control. In addition, we also calculated the density of spots (number of spots/DNA) for the full nucleus and specifically for the Xi, using DAPI sum intensity values (within the full nucleus and the segmented Xi respectively) as a measure of DNA amount. Normalizing by the average nuclear spot density in control cells, we found a higher spot density within the inactive X chromosome area, namely, a 2.5-fold-increase for control and 2.3 for macroH2A2 depleted cells (Figure 7D). On the other hand, we observed a clear reduction in the number of nuclear spots between macroH2A2 and Mcm2. For this reason, we quantified the number of spots in control cells using high-content microscopy, which allows us to image the full population of cells in the sample. For macroH2A1-Mcm2, we obtained similar numbers to those quantified using confocal microscopy. Confirming our previous observation, this analysis showed a significantly lower average number of macroH2A2-Mcm2 spots compared with macroH2A1-Mcm2 (3.72 spots versus 15.05 respectively), and macroH2A1.2 (14.8). The same trend was found in mouse embryonic fibroblasts (MEF cells) for macroH2A1, macroH2A1.2 and macroH2A2 (Figure 7E). Representative confocal images of these PLA assays are shown in Figure 7F.

To validate the interaction between macroH2A1 isoforms and the DNA helicase Mcm, we performed co-immunoprecipitation experiments. To this end, GFP-tagged macroH2A1.1, macroH2A1.2 and macroH2A2 constructs were overexpressed in C2C12 cells synchronized in G1/early S-phase. Immunoprecipitation was performed (48 h after transfection) with a GFP-binding nanobody (GBP) (45) and analyzed by western blotting with antibodies against GFP (to detect the macroH2A isoforms), Mcm2, Mcm4 and Mcm5. In doing so, we found that macroH2A1.1 and macroH2A1.2, but not macroH2A2, were able to pull down endogenous Mcm2 (Figure 7G), Mcm4 and Mcm5 (Supplementary Figure S9A–C). Furthermore, immunoprecipitated endogenous Mcm4 was able to pull-down macroH2A1.1 and macroH2A1.2, but not macroH2A2 (Supplementary Figure S10A, B), agreeing with the PLA experimental results.

### A conserved phenylalanine residue in both macroH2A1 isoforms is essential for Mcm interaction

**Figure 8.**
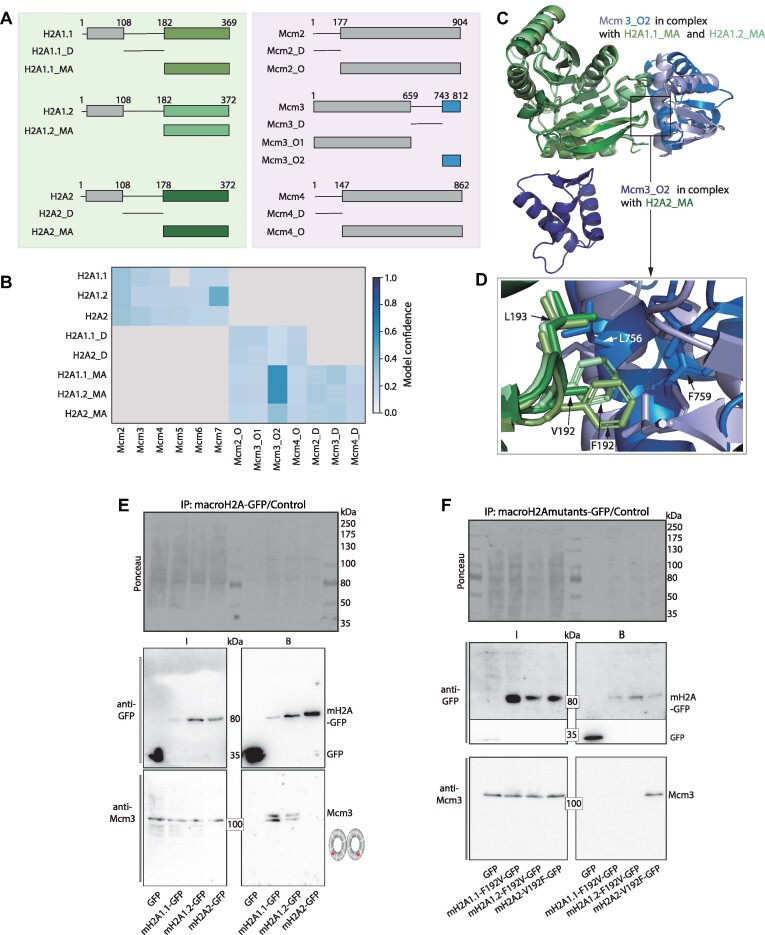
MacroH2A1 isoforms interact with Mcm3 through a conserved Phe residue in their macro domains. (**A**) Schematic illustrating the protein fragments used for AF modeling (full-length Mcm5, 6 and 7 are not shown). macroH2A1.2_D is identical in sequence to macroH2A1.1_D why it was omitted. (**B**) Heatmap showing the model confidences obtained for AF structural models. Labels on the x and y axis indicate the paired protein fragments for structural modeling. Gray fields indicate fragment pairs that were not subjected to structural modeling apart from macroH2A1.1 paired with Mcm5 for which structural modeling failed. (**C**) Superimposition of structural models obtained for the macroH2A domains paired with Mcm3_O2. macroH2A domains are shown in green colors as in A, Mcm3_O2 is shown in different shades of blue. Individual structural models are shown in Supplementary Figure S11. (**D**) Zoom into the interface between the macro domains of macroH2A1.1 and 1.2 and Mcm3_O2 with key residues shown as sticks. (**E**) Co-immunoprecipitation experiments: C2C12 cells were transfected with EGFP or EGFP-tagged macroH2A1.1, macroH2A1.2, or macroH2A2. Cell extracts were analyzed by immunoprecipitation with immobilized GFP-binding nanobody, followed by detection with antibodies against GFP and Mcm3. (**F**) Co-immunoprecipitation experiments were performed as described for (E) but replacing EGFP-tagged macroH2A1.1, macroH2A1.2, or macroH2A2 for their respective mutants: macroH2A1.1-F192V, macroH2A1.2-F192V or macroH2A2-V192F. For (E) and (F), the cut-outs show input/bound GFP and input/bound Mcm fractions. To the right, the scheme of the DNA helicase shows the two hexamers. The position of Mcm3 subunits on the hexamers is indicated with a red star. Two additional replicates for these co-immunoprecipitations are shown in Supplementary Figure S12.

To further elucidate the binding mode between the macroH2A proteoforms and the Mcm complex we employed structural modeling with AlphaFold-Multimer (AF). Due to protein complex size restrictions when running AF, we formed pairs of full length macroH2A proteoforms with individual full-length Mcm subunits. Based on our previous work, where we reported significant decreases in sensitivity of AF for complex structure prediction when using full length sequences, we also designed and paired protein fragments consisting either of individual folded or disordered regions in the macroH2A proteoforms and Mcm subunits (104) (Figure 8A). Of note, folded regions were paired with other folded regions or disordered regions enabling prediction of common modes of protein binding. In total, we conducted 48 AF predictions (Figure 8B, Supplementary Data 1). Ranking the resulting models by model confidence, we observed overall poor confidences with the exception of the macro domains of macroH2A1.1 and macroH2A1.2 when paired with a small C-terminal folded domain in Mcm3 (Mcm3_O2) where structural models yielded modest confidences (0.59 and 0.6, Figure 8B, Supplementary Data 1). Superimposition of the structural models comprising the three macro domains in complex with Mcm3_O2 showed that macroH2A1.1_MA and macroH2A1.2_MA were predicted to bind in very similar ways to Mcm3_O2 while Mcm3_O2 was predicted to bind to a different surface on macroH2A2_MA (Figure 8C), albeit with much reduced confidence (0.35). Closer inspection of the structural models (Supplementary Figure S11) revealed a conserved phenylalanine residue (Phe192) in macroH2A1.1_MA and macroH2A1.2_MA that was predicted to dock into a hydrophobic pocket on Mcm3_O2 (Figure 8D). Interestingly, macroH2A2_MA carries a valine (Val) at position 192 likely explaining why macroH2A2 was predicted (with very low confidence) to bind differently to Mcm3_O2. These predictions are in line with the co-IP results where macroH2A2 was not observed to pull down Mcm subunits.

Next, we validated the predictions by co-immunoprecipitation experiments as in Figure 7G. Incubation with antibodies against Mcm3 confirmed that both macroH2A1 isoforms, but not macroH2A2, were able to pull-down endogenous Mcm3 (Figure 8E and Supplementary Figure S12A, B), indicating that macroH2A1 interaction with Mcm3 immunoprecipitated the full Mcm complex. To confirm the importance of the conserved Phenylalanine residue for macroH2A1-Mcm3 interaction, we mutated Phe (F) 192 in macroH2A1.1 and macroH2A1.2 to valine (V). In addition, we mutated valine (V) 192 in macroH2A2 to phenylalanine (F). We generated these point mutations in the macroH2A-GFP vectors previously used for co-immunoprecipitation. Then, we performed co-immunoprecipitation experiments as described before. Western blot analysis revealed that macroH2A1.1-F192V and macroH2A1.2-F192V mutants were not able to pull-down Mcm3. Therefore, macroH2A1-Mcm3 interaction was disrupted almost completely in F192V mutants. And interestingly, the replacement of valine 192 for phenylalanine in macroH2A2 was enough to restore the interaction with Mcm3 (Figure 8F and Supplementary Figure S12C, D), thus, validating the Alphafold predictions. In summary, we probed the isoform-specific interaction between macroH2A1 and Mcm *in situ* (PLA), which was significantly enriched in the Xi. Furthermore, we showed that this interaction occurs for both macroH2A1 isoforms in a specific manner, docking into a hydrophobic pocket on Mcm3. All this evidence suggests the hypothesis that macroH2A1 interaction with the replicative DNA helicase plays a role in Xi replication synchrony.

### Chromatin loading of the DNA helicase in the Xi is negatively affected by macroH2A1 depletion

**Figure 9.**
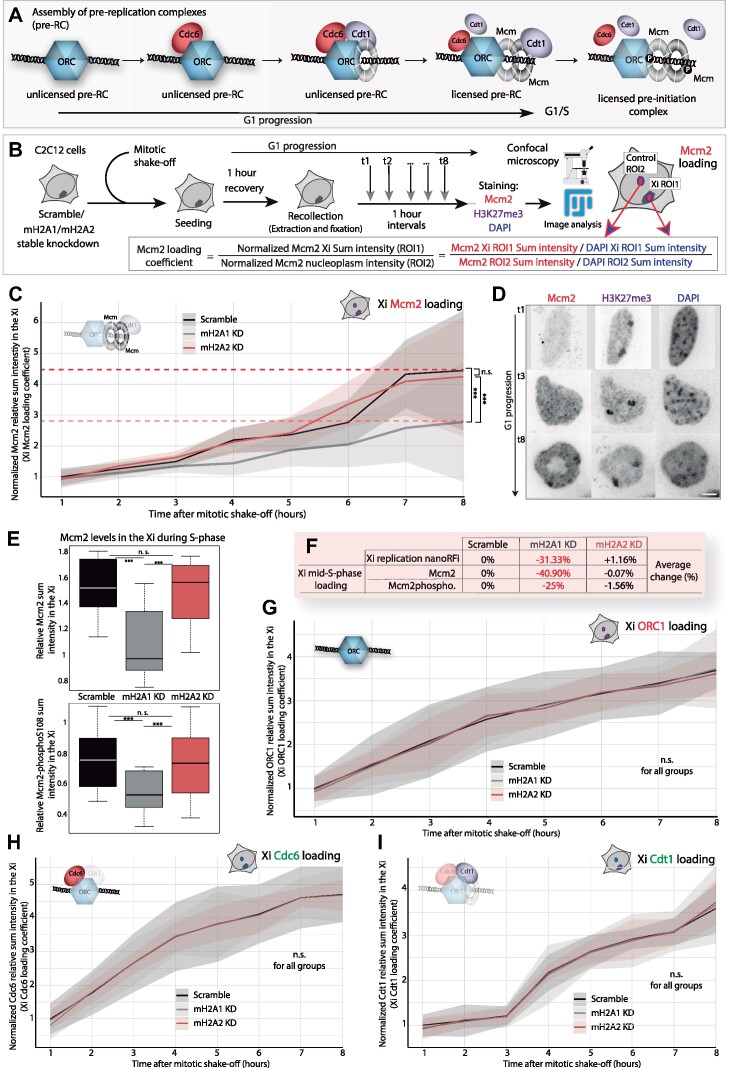
MacroH2A1 depletion, but not macroH2A2, affects the loading of Mcm2 to pre-RCs in the Xi. (**A**) Scheme showing a simplified summary of the different steps of origins activation during G1, from the assembly of unlicensed pre-RC (starting with ORC, Cdc6 and Cdt1-Mcm), to the licensing of pre-RC by the loading of the second hexamer of the DNA helicase Mcm, and their activation to pre-IC at the end of G1 and during S-phase. (**B**) Experimental pipeline and image analysis. C2C12 stable knockdowns were synchronized by mitotic shake-off and seeded onto coverslips. One hour after seeding, eight different time points were collected in one-hour intervals to track G1 progression over time. Cells were extracted and fixed, and afterward immunostained against Mcm2 and H3K27me3. Cells were imaged using confocal microscopy and image analysis was performed with FIJI. H3K27me3 signal was used for Xi ROI selection and calculating Mcm2 loading coefficients (as depicted in the formula). (**C**) Mcm2 loading coefficients for each G1 time point were normalized by the average of control cells at time 1 (t1) and plotted, obtaining Mcm2 G1 loading curves. N-numbers (cells): Scramble 24–28, mH2A1 KD 15–19, mH2A2 KD 15–25, two independent replicates. Representative images for selected time points are shown in for Scramble cells (**D**). The full gallery of images is shown in Supplementary Figure S14. (**E**) Boxplots showing Xi levels of Mcm2 (top) and Mcm2-phosphoS108 (bottom) in extracted and non-synchronized cells imaged using confocal microscopy. Cells replicating the Xi were selected for image analysis as described in (B). Boxplots show relative Mcm2 sum intensity in the replicating Xi, normalized by the average of control cells. N-numbers (cells): Scramble 13, mH2A1 KD 16, mH2A2 KD 15 (Mcm2); 20, 20, 29 (Mcm2-phosphoS108). (**F**) Table summarizing the changes (in percentage) for Mcm2 and Mcm2phosphoS108 levels in the Xi, and the number of nanoRFi (relative to control cells). A reduction of 25%/40.9% on final Mcm2phosphoS108/Mcm2 loading in the Xi was in line with a reduction of 31.3% in the number of replication nanofoci (aka, active origins) for macroH2A1 knockdown cells. Significant changes are highlighted in red. (**G**) ORC1 loading coefficients for each G1 time point were normalized by the average of control cells at time 1 (t1) and plotted, obtaining ORC1 G1 loading curves. N-numbers (cells): Scramble 42–49, mH2A1 KD 40–44, mH2A2 KD 42–43, four independent replicates. The same experimental approach was followed for Cdc6 (N-numbers (cells): Scramble 22–29, mH2A1 KD 22–24, mH2A2 KD 22–24) (**H**), and Cdt1 (*N*-numbers (cells): Scramble 20–22, mH2A1 KD 19–23, mH2A2 KD 19–20) (**I**), two independent replicates. All line plots show normalized average fluorescence values, and error bands show the respective standard deviation. 95% confidence intervals are indicated in the plot as a band. For all boxplots, the box represents 50% of the data, starting in the first quartile (25%) and ending in the third (75%). The line inside represents the median. The whiskers represent the upper and lower quartiles. Statistical significance was tested with a paired two-sample Wilcoxon test (n.s., not significant, is given for *P-*values ≥0.05; one star (*) for *P-*values <0.05 and ≥0.005; two stars (**) is given for values <0.005 and ≥0.0005; three stars (***) is given for values <0.0005). *N*-numbers and *P-*values are shown in Supplementary Table 8 (statistics). Scale bars: 5 μm.

Given these findings, we took a closer look into the assembly of pre-replication complexes (pre-RCs) into origins. This process entails a series of molecular events starting during late mitosis with ORC (origin recognition complex) binding to potential origins, followed by the recruitment of Cdc6, which recruits a first Mcm-Cdt1 complex (105–107). Throughout G1, origins are subsequently licensed by the loading of a second Mcm hexamer (Figure 9A) (108). To determine whether macroH2A1-Mcm2 interplay could affect Mcm loading at origins, we next measured Mcm2 chromatin loading throughout the G1 phase in stable knockdown cells synchronized using mitotic shake-off. Cells were processed during G1 progression in one-hour intervals for eight-time points to cover the whole G1 phase. At each processing time, cells were extracted following the protocol of (50). After fixation, immunostaining was performed to detect Mcm2 and H3K27me3 (used for Xi segmentation) (Figure 9B). All samples were imaged using confocal microscopy and analyzed using FIJI for quantification of Mcm2 loading coefficient in the Xi (Figure 9B-C) and in the nucleus (Supplementary Figure S13A). For the inactive X chromosome, Mcm2 loading curve during G1 showed a progressive increase, with a higher loading coefficient over time compared with global Mcm2 loading (Supplementary Figure S13A). However, the loading coefficient was significantly lower as early as 4 h after mitotic shake-off in macroH2A1 depleted cells, reaching a much lower final loading coefficient at eight h: 2.77 for mH2A1 KD versus 4.44 and 4.24 for control and mH2A2 KD respectively (Figure 9C). On the other hand, quantification of the nuclear loading coefficient for Mcm2 showed a slower loading over the first 5–6 h of G1. The loading became faster from 6 to 8 h and did not show significant differences between control and macroH2A knockdowns, indicating that global Mcm2 loading is not affected by macroH2A depletion (Supplementary Figure S13A). Representative images from selected time points are shown in Figure 9D, and a full gallery for all time points can be found in Supplementary Figure S14.

In addition, we verified these observations by quantifying chromatin-bound Mcm2 global levels in non-synchronized cells using immunostaining and high-content screening microscopy. We also measured the levels of phosphorylated Mcm2 (phosphoS108), the active form of Mcm2 during DNA replication (109,110). The levels of both proteins were measured and normalized by the average of control cells. No significant differences were found in the nuclear levels of Mcm2 and Mcm2-phosphoS108 for all cell lines (Supplementary Figure S13B). Data from Supplementary Figure S13B were further subdivided into G1, S-phase (early, mid and late), and G2 (Supplementary Figure S13C) using DAPI sum intensities as a proxy for DNA content (Supplementary Figure S13D) and EdU profiles (82). This gave us a more accurate distribution of Mcm2 and Mcm2-phosphoS108 levels over the cell cycle and their fluctuations. We observed higher levels of Mcm2 in G1 cells, with higher standard deviation due to increasing Mcm2 accumulation during G1. These levels progressively decrease during S-phase, since Mcm2 dissociation occurs after replication, reaching the lowest values in the G2 phase (111–113). According to previous studies, Mcm2-phosphoS108 showed different cell cycle dynamics, with shallow levels in G1 and G2, and higher levels during S-phase progression due to its activation (Supplementary Figure S13C). Nevertheless, when we measured Mcm2 and Mcm2-phosphoS108 levels in the Xi of mid-S-phase cells (Figure 9E), both were significantly reduced in macroH2A1 depleted cells, 25% less compared with control for Mcm2 and 41% less for Mcm2-phosphoS108 (summarized in Figure 9F). These results are in line with the lower Mcm2 loading at the end of G1 found in macroH2A1 knockdown cells. Representative confocal images of this immunofluorescence, including G1 and the different S-phase substages, are shown for Scramble and knockdown cell lines in Supplementary Figure S15A–C. On the other hand, we obtained similar results to Supplementary Figure S13A-B for global Mcm2 and Mcm2-phosphoS108 levels performing chromatin fractionation of proteins followed by western blotting (Supplementary Figure S13E, F). The chromatin fraction showed no differences in Mcm2 levels between control and macroH2A knockdown cells, confirming that nuclear Mcm2 and Mcm2-phosphoS108 loading levels are not affected by macroH2A1 depletion.

Following the same experimental approach used to calculate Mcm2 G1 loading coefficients (Figure 9B), we investigated earlier steps of pre-RCs assembly. We measured the levels of ORC1 (Figure 9G), Cdc6 (Figure 9H), and Cdt1 (Figure 9I) in the Xi throughout G1. Interestingly, neither Xi nor nuclear loading was affected in macroH2A knockdown cells compared to control cells (Figure 9G–I and Supplementary Figure S16A–C, representative galleries are shown in Supplementary Figure S17 and Supplementary Figure S18), pointing to the specific effect of macroH2A1-Mcm2 interaction on licensing of pre-RC without affecting unlicensed pre-RCs (Figure 9A). Next, we investigated whether depletion of macroH2A affected other chromatin features involved in the licensing and activation of replication origins. Previous studies reported that H4K20me2 is recognized by ORC1 (114), and the histone variant H2A.Z promotes H4K20me2 deposition at replication origins (115). Due to the existing connection between ORC1 and H4K20me2, we performed immunofluorescence detection of H4K20m2, and also H4K20me1 and H4K20me3, which partially overlap with H4K20me2 genome-wide. H4K20me3 has been shown to ensure timely heterochromatin replication at late-firing origins (116) and, in addition, chromatin loading of Mcm hexamers has been associated with di-/tri-methylation of histone H4K20 toward S phase entry (117). After quantification of H4K20 methylation levels in the nucleus and in the Xi, we did not find significant changes for H4K20me1 (Supplementary Figure S19A), and just minor changes for nuclear levels of H4K20me2 between control and macroH2A1 knockdown cells (*P-*value = 0.04562) (Supplementary Figure S19B). This aligns with the data obtained for ORC1, showing no difference in ORC loading to the Xi. Interestingly, for H4K20me3, slight changes were found in the nuclear levels between control and macroH2A1 knockdown cells (*P-*value = 0.02513), and within the Xi (*P-*value = 0.01844) (Supplementary Figure S19C). Representative images of H4K20me1/2/3 are shown in Supplementary Figure S19D. Changes in H4K20me3 levels in the Xi, even though modest, could be related to the reduced loading of Mmc2 in macroH2A1 knockdown cells, and with the Xi decondensation phenotype observed (Figure 5F). H4K20me3 is produced from H4K20me1 and, on its own, can compact chromatin fibers and regulate chromatin structure (118,119).

**Figure 10.**
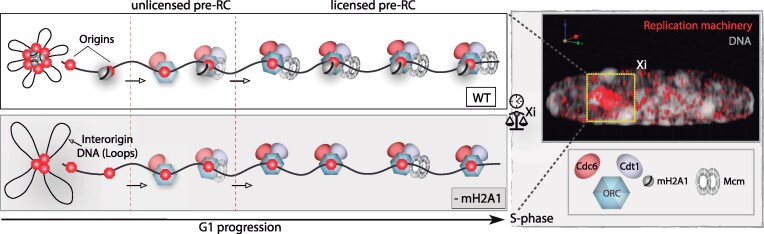
Model representing the role of macroH2A1 in Xi replication dynamics. Replication dynamics depend on two different factors that together affect replication rate: synchrony or number of active origins at a certain time, and replication fork speed progression. The first one rests on the assembly of different components of the pre-replication complexes (pre-RCs) during G1, part of which is the replicative DNA helicase Mcm. We propose that the isoform-specific association of macroH2A1 with some of the Xi replication origins is a factor regulating the formation of chromatin loops and the accessibility of these origins to pre-RC assembly. Hence, Mcm chromatin loading is reduced after macroH2A1 depletion. The reduced Mcm G1 loading turns into less Mcm2 in the inactive X chromosome during S-phase, and consequently less Mcm2-phosphoS108, the active form of this helicase during replication. Finally, this causes a decrease in the number of active origins, which negatively affects the replication rate. However, the effects of macroH2A1 depletion are in part counterbalanced by the impact of both macroH2A isoforms in replication fork speed, by hampering the progression of the replication fork machinery, as shown in Figure 2.

In conclusion, lower Mcm2 levels within the replicating Xi can be interpreted as the final output of less loading during G1 or more removal of Mcm2 from chromatin during the G1 phase. Interestingly, these levels are quasi-proportional to the reduction in the number of replication origins, around 31% less nanoRFi for macroH2A1 depletion. Hence, fewer replication origins firing upon macroH2A1 knockdown can be explained by reduced Mcm2 loaded to pre-RCs and reduced Mcm2-phosphoS108 into pre-ICs, negatively impacting the Xi replication rate. Therefore, macroH2A1 depletion affects both replication timing and synchrony with a decreased number of licensed replication origins, accompanied by longer DNA loop sizes. To sum up our findings, we propose a model in which a fraction of the origins of replication in the Xi is regulated by macroH2A1 enrichment and its interaction with Mcm2 (Figure 10, full model in Supplementary Figure S20). Loading of pre-RCs at origins follows a temporal order throughout G1, with macroH2A1 playing a role in enhancing the licensing of pre-RC by Mcm loading. The macroH2A1-Mcm2 interaction may act to stabilize the loading of the DNA helicase. By this mechanism, macroH2A1 may regulate the synchronous firing of associated origins in the inactive X chromosome. Thus, these observations strengthen the link between nucleosome composition, chromatin structure (chromatin loops), and the regulation of replication origins. This regulation comprises the formation and/or stabilization of DNA loops in interorigin DNA regions, which affects the clustering of origins. Fewer origins are properly clustered and are synchronously activated when macroH2A1 is depleted, as shown by decreased Mcm2 loading to pre-RCs at Xi during G1, and consequently, less Mcm2 activation (Mcm2-phosphoS108) during S-phase.

## Discussion

In this study, we addressed the role of the histone variant macroH2A, a hallmark of the inactive X chromosome, in its replication dynamics. In brief, we showed that macroH2A-containing nucleosomes slow down DNA replication fork progression. These nucleosomes are more stable than their canonical counterparts (18), are worse substrates for nucleosome remodeling (2,120), and stop RNAPII activation of transcription (3). Moreover, macroH2A has been proposed to stabilize higher-order chromatin structure (16) and coordinate chromatin looping (121), possibly via the ability of the non-histone domain to form dimers (122). The linker domain of macroH2A resembles the C-terminal half of the linker histone H1 responsible for the binding and stabilization of internucleosomal DNA (1,123). Eukaryotic DNA replication, just like transcription, happens in the context of chromatin and nucleosomes. Our results clearly show that macroH2A depletion speeds up the replication progression and helicase unwinding rate (Figure 2). Therefore, we propose that being harder to evict, macroH2A nucleosomes hamper the progression of the replication fork machinery, which needs to open up the double helix before DNA can be copied.

But nucleosomes are not just stumbling blocks, their fine-tuned regulated positioning rules DNA access and ensures proper transcription start site selection at promoters (124–127). Until now, it was unclear whether nucleosome composition and organization have a similar role in replication, but our findings, together with recent studies (115,128–130), provide new insights in this direction. Beyond Xi fork progression, we have demonstrated that macroH2A1 stabilizes chromatin loops, ultimately increasing the synchrony of replication origin firing in the inactive X chromosome. DNA replication origins in mammalian cells have been shown to spatially associate forming a cluster of origins and chromatin loops (131–133). In this regard, it is still debatable whether chromatin loops determine origin spacing and, thus, replicon size or whether replication characteristics, such as origin spacing, can rather determine chromatin loop size. Yet, previous studies showed that origin activation is influenced by the number of contacts established between chromatin fragments containing origins (134). MacroH2A1 downregulation led to an increase in the length of the loops, which, combined with the decrease in nanoRFi in the Xi, suggests that macroH2A1 participates in the formation or stabilization of loops at replication origins. An earlier report (89) showed that faster/slower replication fork speed leads to less/more active origins and longer/shorter origin spacing. Although this could also contribute to fewer origins and longer loops/origin spacing in the case of macroH2A1 depletion, it cannot explain what we found with macroH2A2 depletion, where slower fork rate is not compensated by more origins firing with shorter origin spacing/loops. We propose that the enrichment of macroH2A1 on the Xi contributes to its origin firing synchrony. The finding that macroH2A1 interacts with Mcm helicase complexes and affects their loading to pre-RCs throughout the G1 phase, provides a mechanistic explanation of the effect of this histone variant on Xi replication synchrony. Consequently, in the absence of this histone variant, the loading of Mcm complexes might be slowed down and ultimately reduced, which leads to a decrease in the number of active origins that fire simultaneously in the Xi (Figure 10 and Supplementary Figure S20).

Although macroH2A1 depletion does not cause global Xi reactivation (14), it changes the chromatin environment in such a way that directly affects replication origin activation. The latter would negatively regulate synchronous origin firing, reducing pre-RC loaded Mcm and affecting the Xi replication schedule. We found that macroH2A1 plays a more prominent role in maintaining Xi synchronous replication program than macroH2A2. Fittingly, Barrero *et al.* (12) have shown that macroH2A1 is a much more prominent barrier to cellular reprogramming than macroH2A2. Still, it also has been proposed that both macroH2A isoforms provide a redundant silencing layer at pluripotency genes, challenging reprogramming (13). On the other hand, studies in macroH2A knockout mice indicate that macroH2As affect gene expression, with macroH2A1 and macroH2A2 acting synergistically on the expression of some genes and having opposing effects on others (15). Other studies have also reported a more ambiguous role of macroH2A in transcriptional regulation: its ablation deregulates transcription in both directions, often in an isoform-specific manner (8,135). Nonetheless, macroH2A contributes to the robustness of gene expression programs (136). Downregulation of macroH2A1 could increase chromatin accessibility in the Xi, resulting in improved accessibility for the transcription machinery (2,137) and affecting Xi replication dynamics.

Interestingly, the role of other histone variants in origin activation has been recently reported. For example, the regulatory function of the histone variant H2A.Z facilitates the licensing and activation of early replication origins by promoting H4K20me2 deposition, which is required for ORC1 (origin recognition complex subunit 1) binding (115). In our study, we have shown that histone acetylation levels (H3K9ac and H4K8ac) are undisturbed by macroH2A depletion, together with no changes in ORC1 loading and H4K20me2 levels. Moreover, a recent publication has shown the role of the ORC in establishing nucleosome organization at replication origins, suggesting that proper nucleosome positioning around origins may be critical for replication (130). In addition to the standard role of ORC as the Mcm loader, ORC orchestrates the organization of origin-adjacent nucleosomes, and this organization is functionally important for replication. In our study, we found a direct interaction between nucleosome composition and Mcm loading to be important for replication synchrony, as a mechanism independent of ORC. Fittingly, the depletion of macroH2A1 seems to be correlated with small changes in H4K20me3 levels, previously associated with Mcm chromatin loading (117). It has been shown that specific nucleosome positioning at gene bodies and promoters affects both transcription and replication origins (124–126). It also has been shown that the ORC-associated protein (ORCA) promotes origin licensing in heterochromatin, maintaining adequate Mcm2-7 loading rates by its ability to bind histone repressive marks (H3K9me3, H3K27me3, and H4K20me3) (138). Depletion of ORCA results in loss of ORC association to chromatin and reduction of Mcm binding (139). We showed that macroH2A1 depletion does not affect unlicensed pre-RCs assembly of ORC1, Cdc6, and Cdt1 into the origins. Yet, we propose that macroH2A1-containing nucleosomes have a novel role in setting the interorigin distances and origin activation: macroH2A1-Mcm2 interaction specifically facilitates the licensing of pre-RCs, stabilizing loaded Mcm complexes by protein-protein interaction and maintaining the replication program in the Xi.

*Per se*, macroH2A is a barrier to replication, but in an isoform-specific mode contributes positively to origin firing. In our study, we found that both macroH2A1 isoforms interact with the DNA helicase Mcm, in clear contrast with macroH2A2. We could also show that the NAD+-derived metabolites binding pocket of macroH2A1.1 does not play a role in this context (26,27). Rather, a conserved Phe residue in both macroH2A1.1 and macroH2A1.2 is essential for interaction with the subunit Mcm3. This subunit of the Mcm complex is unique in terms of structure, harboring a hydrophobic pocket in its C-terminal domain where macroH2A1 docks. Uniquely, Mcm3 C-terminal domain is not involved in the formation of the Mcm complex and, furthermore, is not resolved in cryoEM structures (likely due to its regulatory functions) (140). Adding on, macroH2A isoforms do not undergo the same post-translational modifications (PTMs). For example, the linker domain can be phosphorylated on serine 137 (141,142), and specifically the linker of macroH2A1 can be ADP-ribosylated on serine 146 (143). Therefore, it is known that macroH2A proteins can be poly-ADP-ribosylated, ubiquitinated, methylated, and phosphorylated in an isoform-specific manner (144), but the functional consequences of these are not yet well-known. After discarding the role of ORC1, and showing the effect of macroH2A1 in loop formation and Xi decondensation, we contemplate the prospective interplay of macroH2A1 with chromatin remodelers as the key feature of this isoform in Xi replication synchrony, likely involving isoform-specific PTMs. Moreover, we cannot rule out the prospective role of Mcm3 post-translational modifications. MacroH2A-containing nucleosomes can limit chromatin remodeling and transcription events through SWI/SNF (2). Within this context, we propose that the proper activation of origins during S-phase relies on the interaction between the Mcm helicase and macroH2A1-containing nucleosomes, thus, conditioned by nucleosome organization and (lack of) transcription events in the inactive X chromosome. This may be directly mediated by the recruitment of regulatory factors or indirectly through changes in chromatin organization, and probably regulated by changes in macroH2A isoforms expression during development (145). Ultimately, switches in replication timing, like the one reported for the Xi in macroH2A1 depleted cells, can be initiated by changes in chromatin features.

Back to the roots, highly synchronous replication is explained by the stochastic firing of a large number of closely located origins (146–148), which can proceed in the absence of transcription. Thus, cells transition from slow and asynchronous, to fast and synchronic replication as S-phase progresses. It has been shown that changes in transcription quantitatively affect all three parameters of the replication program: origin activation, fork progression, and replication timing (149). This tradeoff results in the need to coordinate replication timing with transcription during early S-phase, accomplished by dynamic changes in replication-associated epigenetic marks like histone variants and histone modifications (150). For the Xi, rapid and synchronic replication co-exists with transcriptional quiescence. At present, we found that the removal of one of the later epigenetic marks of the Xi, macroH2A1, leads to a loss in the number of active origins. In this context, macroH2A1 may provide molecular memory to Xi synchronous replication pattern. This study reveals a new dimension to macroH2A context- and isoform-specific roles, where macroH2A1 emerges as a new player preserving Xi replication dynamics. Hence, the role of macroH2A1 in X chromosome inactivation appears to be a remarkable system to study the molecular events that initiate or maintain replication-timing switches. Of particular interest for this goal, will be to inquire when such switches occur during development and which hierarchy rules them.

## Supplementary Material

gkae734_Supplemental_Files

## Data Availability

Renewable biological materials will be made available upon request from the corresponding author M. Cristina Cardoso (cardoso@bio.tu-darmstadt.de). ChIP-seq data in WT MEFs were retrieved from GEO-database (GSM835828) (151). The code used in this study for the assessment of DAPI intensity classes (Supplementary Figure S5F) is available at https://bioimaginggroup.github.io/nucim/ and was published in (69) and (88). All our data sets have been deposited and are available at https://doi.org/10.48328/tudatalib-1344.2

## Notes

Present address: Corella S. Casas-Delucchi, The Institute of Cancer Research, Chester Beatty Laboratories, London SW3 6JB, UK.

Present address: Cathia Rausch, Luxembourg Centre for Systems Biomedicine, University of Luxembourg, 6, avenue du Swing, L-4367, Belvaux, Luxembourg.
