# Supplementary material for: Histone variant macroH2A1 regulates synchronous firing of replication origins in the inactive X chromosome
Source: Nucleic Acids Res. 2024 Aug 27;52(19):11659–88. doi: 10.1093/nar/gkae734 (PMC11514477; doi:10.1093/nar/gkae734)
Supplement: gkae734_Supplemental_Files [file gkae734_supplemental_files.zip › Arroyo et al._Supplementary Information.pdf]

SUPPLEMENTARY MATERIAL

Histone variant macroH2A1 regulates synchronous firing of replication origins in the inactive X chromosome

Arroyo, Maria[1*]; Casas-Delucchi, Corella S.[1,7]; Pabba, Maruthi K.[1]; Prorok, Paulina[1]; Pradhan, Sunil. K.[1]; Rausch, Cathia[1,8]; Lehmkuhl, Anne[1]; Maiser, Andreas[2]; Buschbeck, Marcus[3]; Pasque, Vincent[4]; Bernstein, Emily[5]; Luck, Katja[6]; Cardoso, M. Cristina[1,*]

*Corresponding author

1. Cell Biology and Epigenetics, Department of Biology, Technical University of Darmstadt, 64287 Darmstadt, Germany
2. Faculty of Biology and Center for Molecular Biosystems (BioSysM), Human Biology and BioImaging, LMU Munich, Munich 81377, Germany.
3. Program of Myeloid Neoplasms, Program of Applied Epigenetics, Josep Carreras Leukaemia Research Institute (IJC), Germans Trias i Pujol Research Institute (IGTP), Campus Can Ruti, Camí de les Escoles, 08916 Badalona, Barcelona, Spain.
4. Department of Development and Regeneration, Leuven Stem Cell Institute, Leuven Institute for Single-cell Omics (LISCO), KU Leuven-University of Leuven, 3000 Leuven, Belgium.
5. Department of Oncological Sciences, Icahn School of Medicine at Mount Sinai, Tisch Cancer Institute, New York, New York 10029, USA.
6. Institute of Molecular Biology (IMB) gGmbH, 55128 Mainz, Germany.
7. Present address: Corella S. Casas-Delucchi, The Institute of Cancer Research | Chester Beatty Laboratories, London SW3 6JB, UK.
8. Present address: Luxembourg Centre for Systems Biomedicine, University of Luxembourg, 6, avenue du Swing, L-4367 Belvaux, Luxembourg.

Further information and requests for resources and reagents should be directed to and will be fulfilled by the lead contact M. Cristina Cardoso cardoso@bio.tu-darmstadt.de.

Supplementary figures and legends

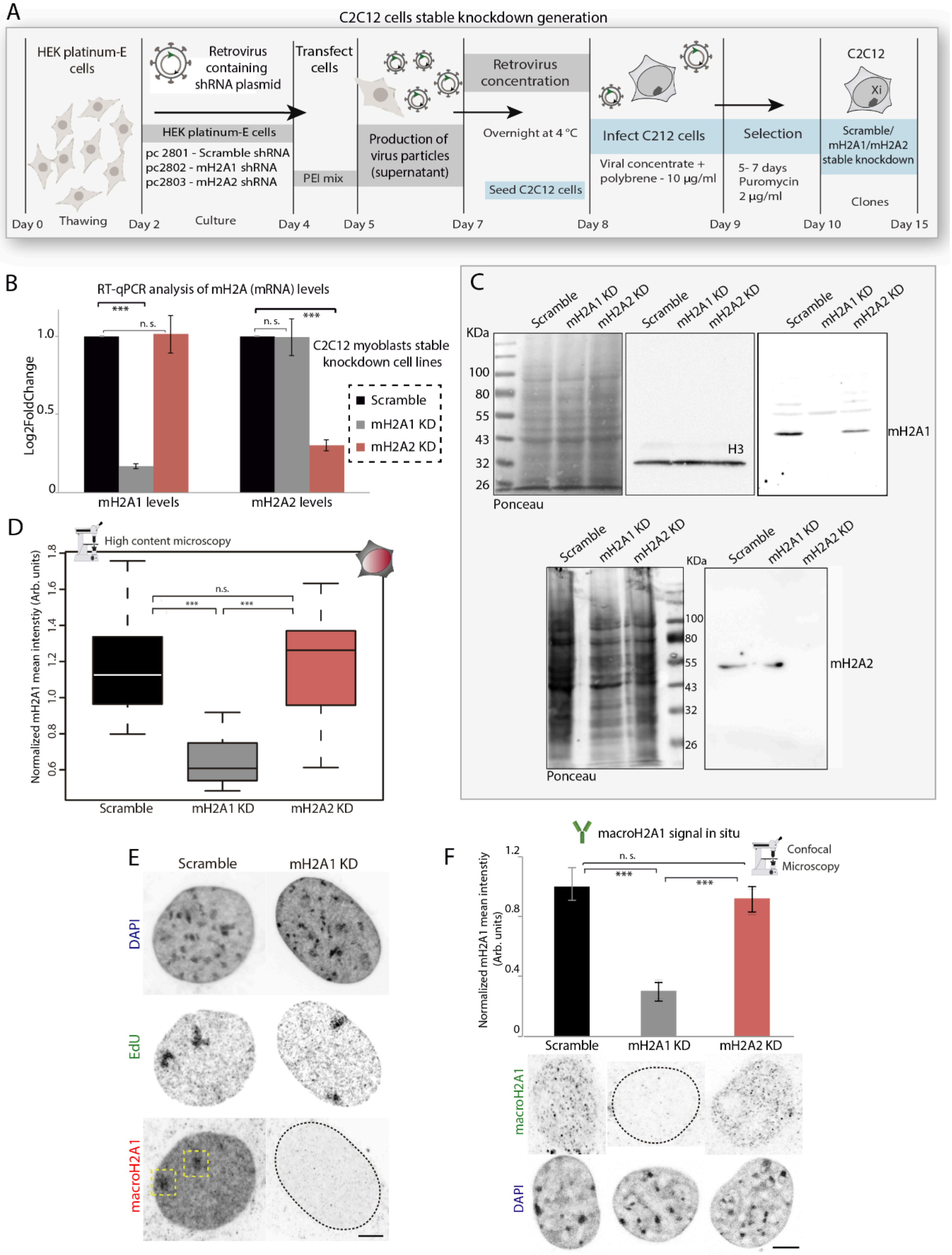

Figure S1. Establishment and characterization of macroH2A1 and macroH2A2 stable knockdown cell lines. (A) Scheme of the experimental pipeline for the knockdown generation. HEK platinum-E cells were infected with a retrovirus containing the shRNA plasmids (Scramble, mH2A1, and mH2A2 shRNAs). After the production of viral particles, the supernatant was collected and the retrovirus was concentrated to infect C2C12 mouse myoblasts. Infected cells were selected with puromycin. PEI = Polyethylenimine. **(B)** Knockdown confirmation was performed by reverse transcriptase qPCR. The mRNA levels of macroH2A1 and macroH2A2 were measured after total RNA extraction and cDNA synthesis. MacroH2A1 and macroH2A2 knockdown C2C12 cells showed a clear and specific decrease of macroH2A1 and macroH2A2 mRNA respectively (Independent replicates: 7, 7, 5 (macroH2A1 levels); 6, 7, 6 (macroH2A2 levels)). Western blot analysis further confirmed macroH2A1 and macroH2A2. **(C)** depletion at the protein level. Ponceau staining was used to control the total amount of protein loaded. **(D)** Immunofluorescence followed by high-content microscopy also shows a drastic reduction in macroH2A1 levels without affecting macroH2A2 (primary antibody from Upstate, 07-219). N-numbers (Cells): Scramble 32, mH2A1 KD 43, mH2A2 KD 47. **(E)** Confocal images showing macroH2A1 distribution for control cells and its enrichment in the inactive X chromosome during mid-S-phase. Replicating Xi chromosomes (yellow squares) can be visualized as a bright nuclear macrodomain by the EdU signal. Almost no macroH2A1 signal is visible for knockdown cells. **(F)** Quantification of macroH2A1 immunofluorescence performed with another antibody (1). Representative images from confocal microscopy are shown at the bottom, with lower macroH2A1 levels for the knockdown cells. N-numbers (Cells): Scramble 10, mH2A1 KD 11, mH2A2 KD 15. Two independent replicates.

Barplots show the average value of the distribution and the whiskers represent the standard error with a 95% confidence interval. For the boxplot, the box represents 50% of the data, starting in the first quartile (25%) and ending in the third (75%). The line inside represents the median. The statistical significance was tested with a paired two-sample Wilcoxon test (n.s., not significant, is given for p-values ≥ 0.05; one star (*) for p-values < 0.05 and ≥ 0.005; two stars (**) is provided for values < 0.005 and ≥ 0.0005; three stars (***) is provided for values < 0.0005). N-numbers and p-values are shown in Supplementary Table 8 (Statistics). Scale bars = 5 μm.

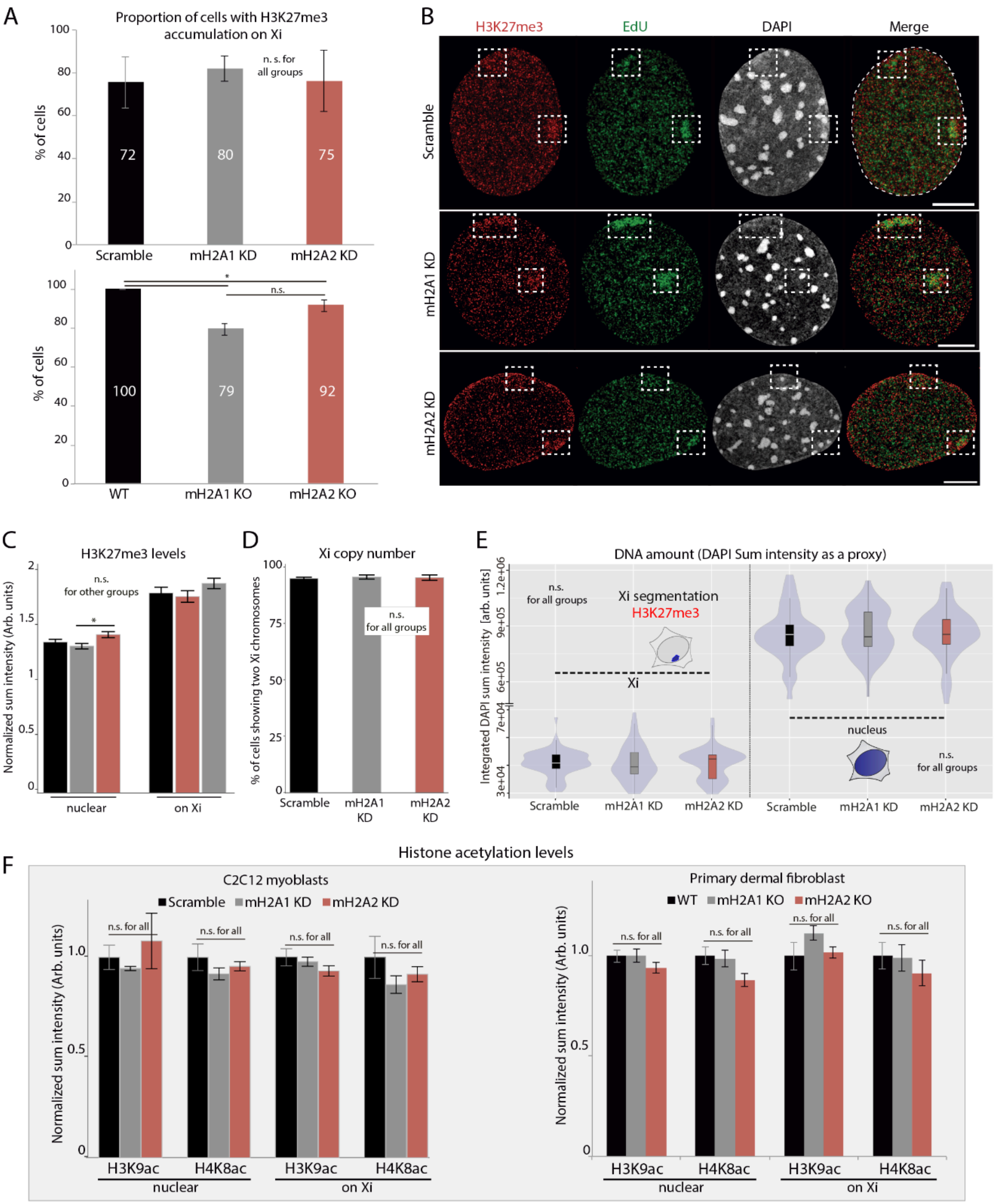

Figure S2. Levels of histone modifications in macroH2A1 and macroH2A2 knockdown and knockout cells. (A) Barplots represent the percentage of cells showing H3K27m3 accumulation on the Xi, which is constant in both knockdown and knockout cells compared to the control cells. N-number (cells): Scramble 86,

mH2A1 KD 161, mH2A2 KD 126; WT 35, mH2A2 KO 49, mH2A2 KO 51. Two independent replicates. Representative confocal images from this immunofluorescence are shown in **(B)**. H3K27me3 accumulation in the Xi is highlighted with white squares. **(C)** Barplots showing the nuclear and the local levels of H3K27me3 in the Xi. N-numbers (cells): Scramble 222, mH2A1 KD 200, mH2A2 KD 232, five independent replicates. **(D)** Barplot showing the quantification of Xi copy number (number of Xi clusters visualized by H3K27me3 accumulation) in control and macroH2A KD cell lines. N-numbers (cells): Scramble 429, mH2A1 KD 351, mH2A2 KD 451. Four independent replicates. **(E)** Violin plots show the DNA amount (DAPI sum intensity) in the Xi after segmentation using the H3K27me3 signal and in the full nucleus. N-numbers (cells): Scramble 65, mH2A1 KD 59, mH2A2 KD 63. **(F)** Barplots showing the nuclear and the local levels of histone acetylation in the Xi (H3K9ac and H4K8ac). Both H3K9 and H4K8 acetylation remained unaffected in macroH2A1 and macroH2A2 deficient cells, as measured on confocal images of immunostainings in situ. N-numbers (cells): Scramble 295-304, mH2A1 KD 363-414, mH2A2 KD 314-463. Five independent replicates for H3K9ac and six for H4K8ac; WT 17-13, mH2A1 KO 25-12, mH2A2 KO 30-10 (nuclear); WT 15-17, mH2A1 KO 12-12, mH2A2 KO 13-18 (on Xi). Two independent replicates.
Barplots show the average value of the distribution and the whiskers represent the standard error with a 95% confidence interval. The violin plot depicts the density curves of the numeric data. Statistical significance was tested with a paired two-sample Wilcoxon or One-Way ANOVA test for histone acetylation (n.s., not significant, is given for p-values ≥ 0.05; one star (*) for p-values < 0.05 and ≥ 0.005; two stars (**) is provided for values < 0.005 and ≥ 0.0005; three stars (***) is given for values < 0.0005). P-values are shown in Supplementary Table 8 (Statistics). Scale bars = 5 µm.

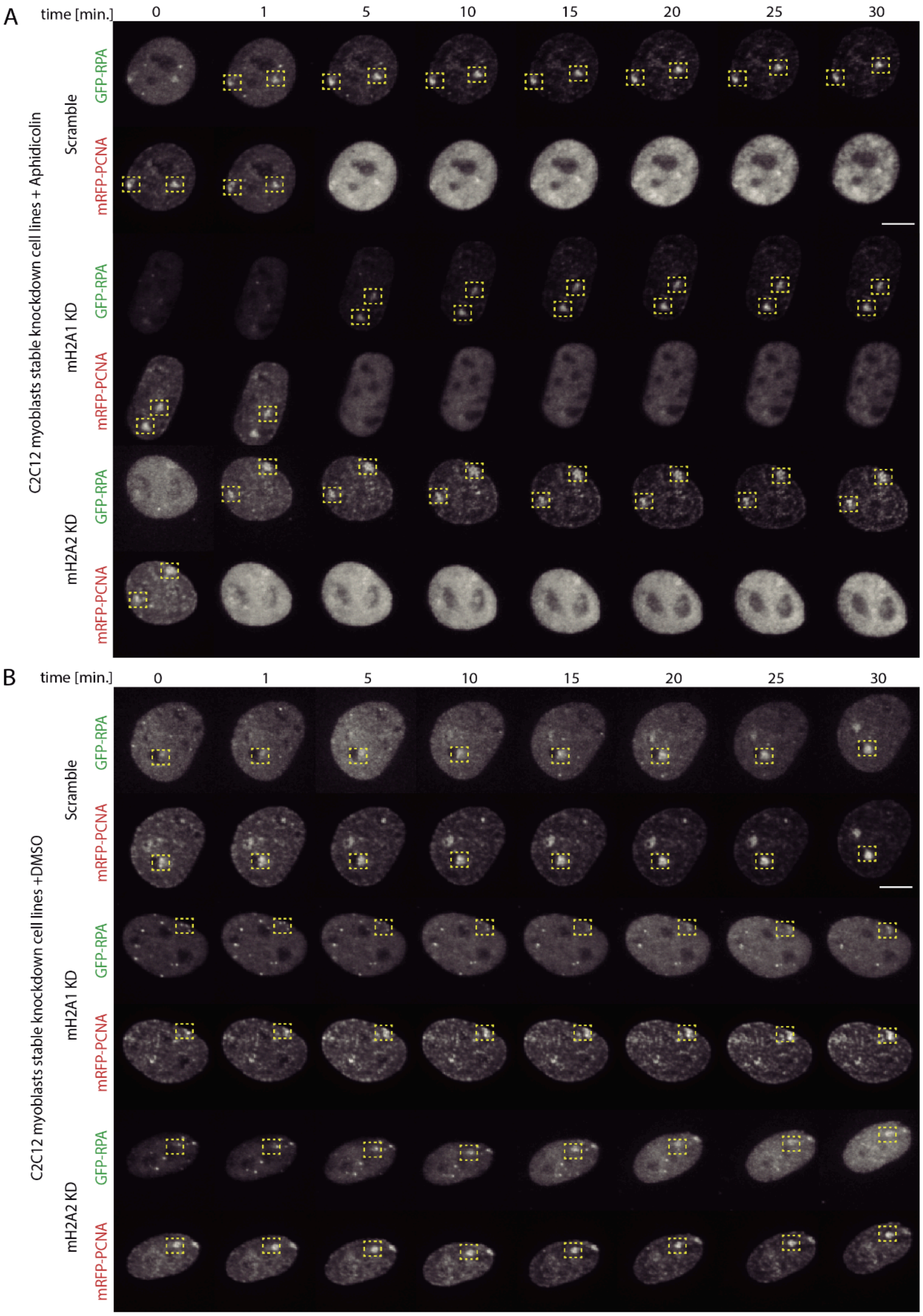

Figure S3. MacroH2A stable knockdown cell lines show increased levels of DNA unwinding and helicase activity on the Xi after aphidicolin treatment. Representative confocal time-lapse microscopy images of aphidicolin **(A)** and DMSO-treated **(B)** cells at different time points over 30 minutes. Aphidicolin-treated cells show RPA accumulation and PCNA dissociation from replication foci. Cells treated with DMSO depict the normal progression of S-phase. Dashed yellow boxes highlight the replicating Xi chromosomes. Scale bar: 10 μm.

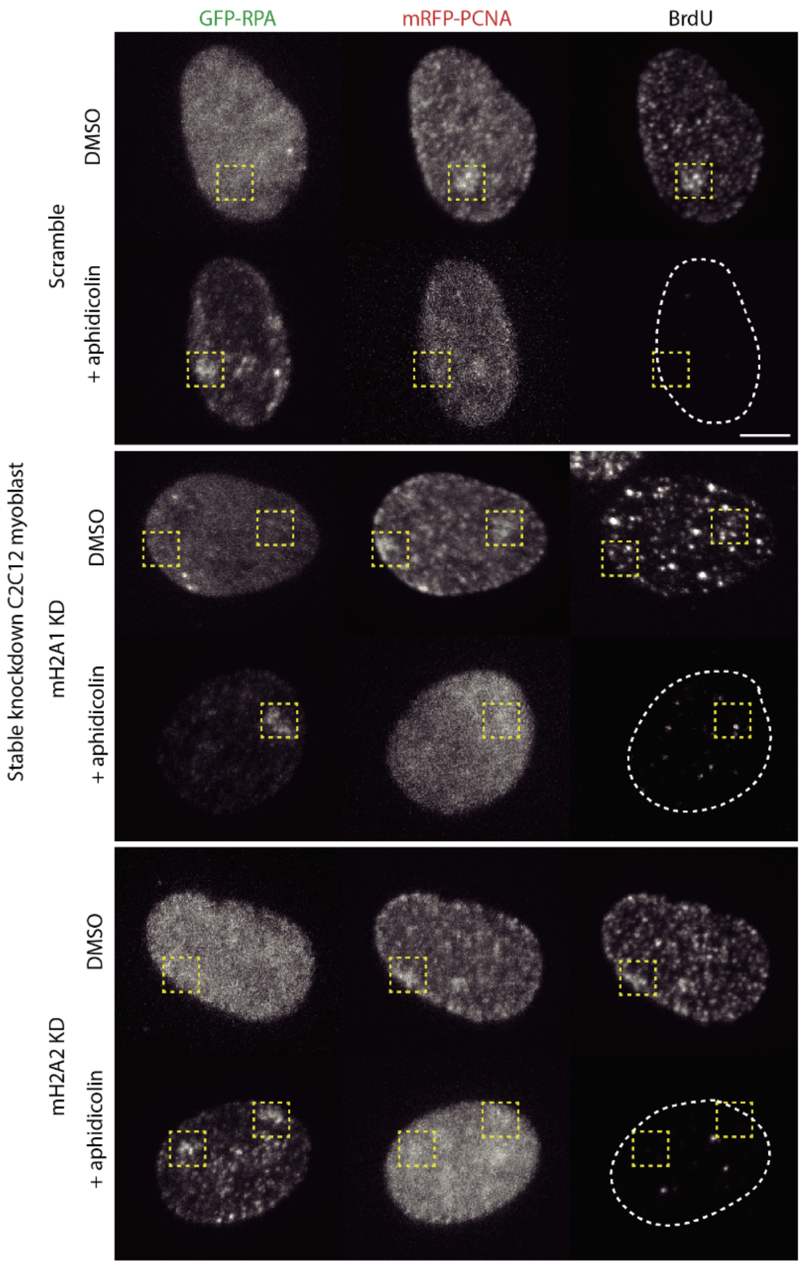

Figure S4. Aphidicolin treatment efficiently halts DNA polymerization, leading to helicase unwinding. Representative confocal images show the localization of GFP-RPA, mRFP-PCNA, and BrdU signals in C2C12 mid-S-phase cells following treatment with DMSO or aphidicolin. Aphidicolin-treated cells were confirmed to have a lack of BrdU incorporation. Dashed yellow boxes highlight replicating Xi chromosomes. Scale bar: 10 μm.

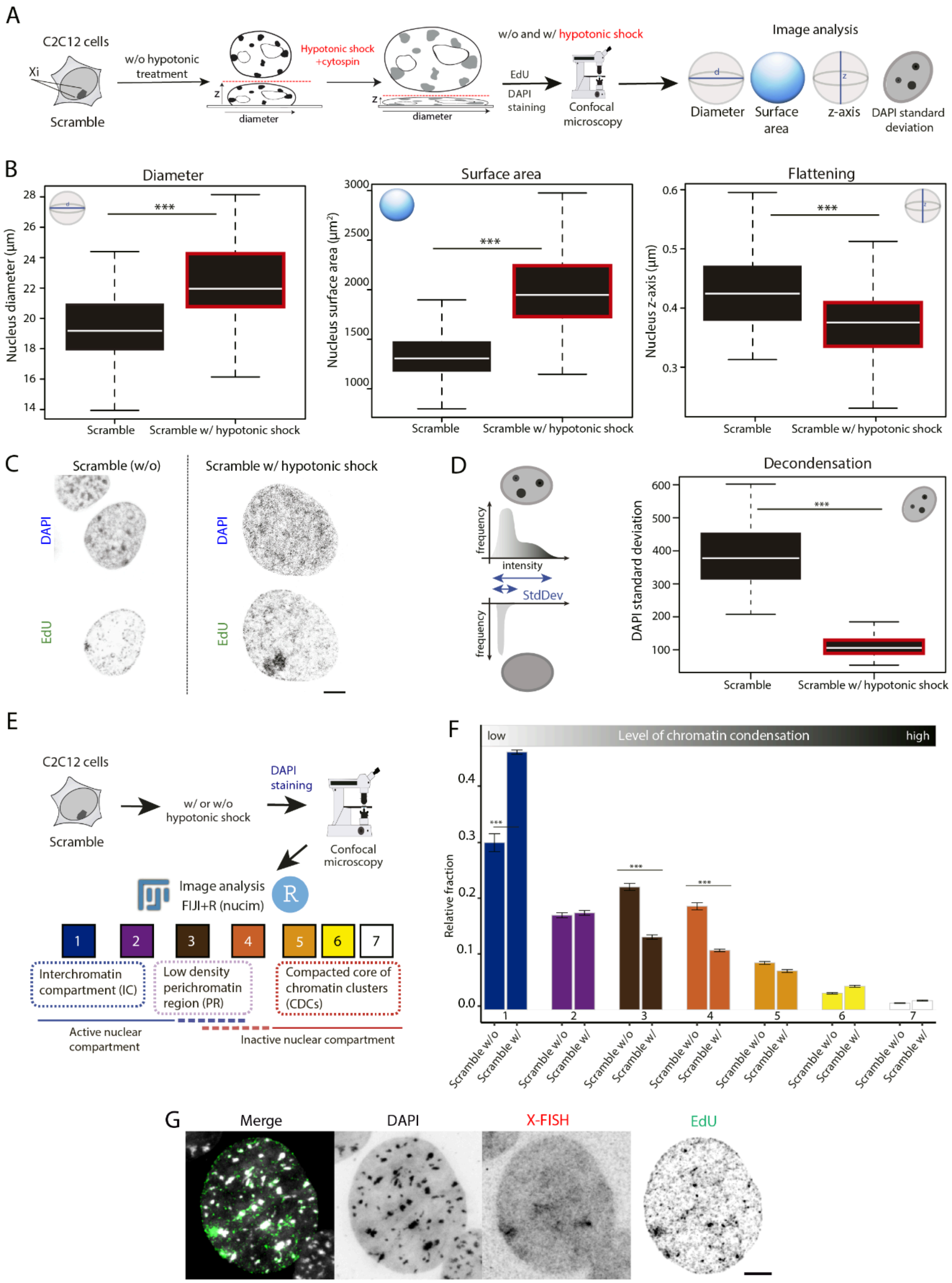

Figure S5. Effects of the hypotonic treatment in replication foci resolution, nucleus flattening, and chromatin decondensation. (A) A diagram representing the morphology of non-treated cells versus

hypotonically resolved nuclei. For image analysis, DAPI stained cells without (w/o) and with (w/) hypotonic treatments were imaged using confocal microscopy, and, then, the diameter, surface area, z-axis, and DAPI standard deviation were measured with Volocity software. Flattening was calculated by dividing the nuclei volume by its surface area. **(B)** Boxplots showing the analysis results described in (A) comparing w/o and w/ hypotonic treatment. N-numbers (cells): Scramble 126, Scramble w/ 132. Two independent replicates. Representative images for replicating cells are shown in **(C)**. Boxplots for DAPI standard deviation values are shown in **(D)**, including an explanatory diagram: chromatin decondensation events reduce DAPI standard deviation values since they homogenize DNA intensity signals. N-numbers (cells): Scramble 157, Scramble w/ 135. Two independent replicates. **(E)** Pipeline of the 3D analysis of chromatin structure after hypotonic treatment. Nucim was used to analyze confocal images, using the DAPI signal to calculate the relative fraction of seven different DNA compaction categories. The results of this analysis are shown in **(F)** as a barplot. N-number (cells): Scramble 17, Scramble w/ 12. Two replicates. **(G)** Representative confocal images of X-FISH: C2C12 mouse myoblasts were replication labeled, then hypotonically treated, and subjected to X-FISH.

For boxplots, the box represents 50% of the data, starting in the first quartile (25%) and ending in the third (75%). The line inside represents the median. Barplots show the average value of the distribution and the whiskers represent the standard error with a 95% confidence interval. The violin plot depicts the density curves of the numeric data. The statistical significance was tested with a paired two-sample Wilcoxon test (n.s., not significant, is given for p-values ≥ 0.05; one star (*) for p-values < 0.05 and ≥ 0.005; two stars (**) is provided for values < 0.005 and ≥ 0.0005; three stars (***) is provided for values < 0.0005). N-numbers and p-values are shown in Supplementary Table 8 (Statistics). Scale bars = 5 μm.

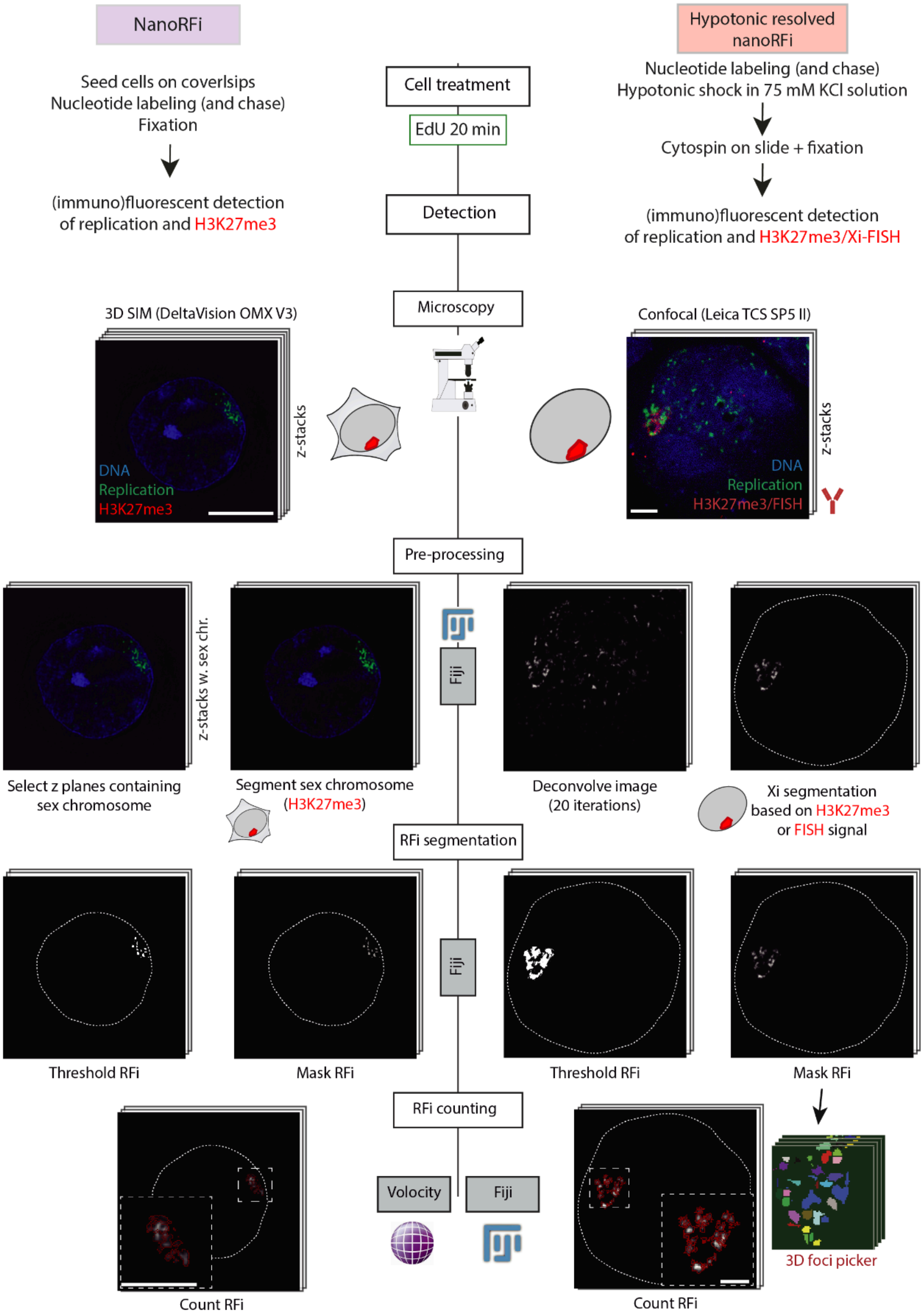

Figure S6. Image analysis pipelines of the different procedures to quantify the number of Xi replication foci. On the left, is the analysis of nanoRFi using 3D SIM, preprocessing with FIJI and Volocity for foci counting. On the right, the pipeline for the analysis of hypotonically resolved replication foci, imaged using confocal microscopy, image preprocessing using FIJI, and foci counting using the FIJI plugging 3D_foci picker.

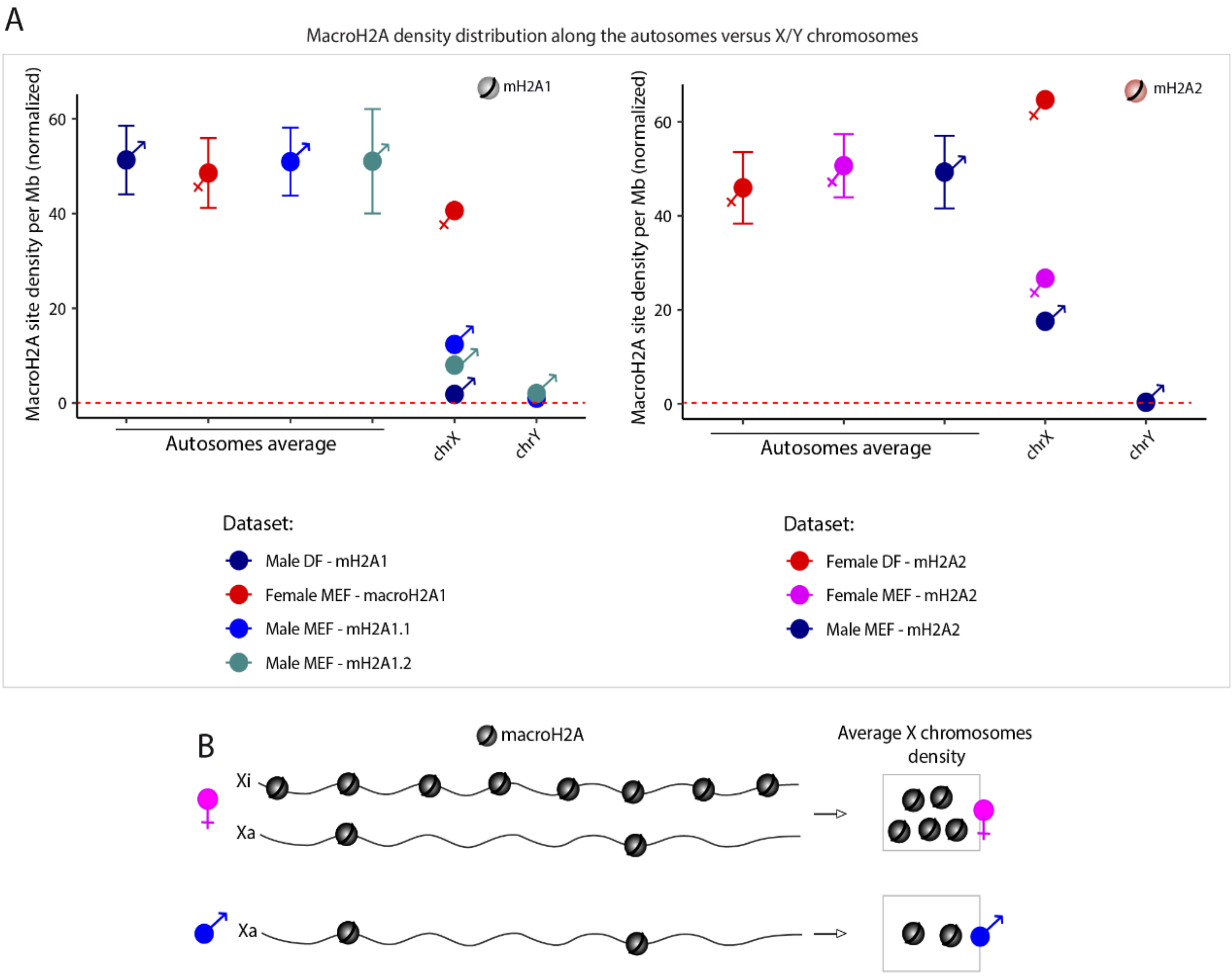

Figure S7. ChIP-seq comparative analysis of macroH2A1 and macroH2A2 density in the autosomes versus chromosomes X and Y. (A) MacroH2A1/macroH2A2 density in mouse male/female cell lines. The density was evaluated separately on autosomes and sex chromosomes, by measuring the number of histone variant enriched sites per Mb on each chromosome. The density obtained was normalized to the total number of macroH2A1/macroH2A2 sites found across the cells compared. **(B)** Explanatory scheme showing the higher density of macroH2A isoforms in the Xi compared with the active X (Xa) in a simplified manner. The average density per megabase in the X chromosome of female cell lines is an average of active and inactive X chromosomes. However, in male cell lines, the density of macroH2A isoforms per megabase corresponds only to the X(a), allowing us to infer a much higher density of macroH2A in the inactive X of female cell lines compared with the Xa and with the autosomes.

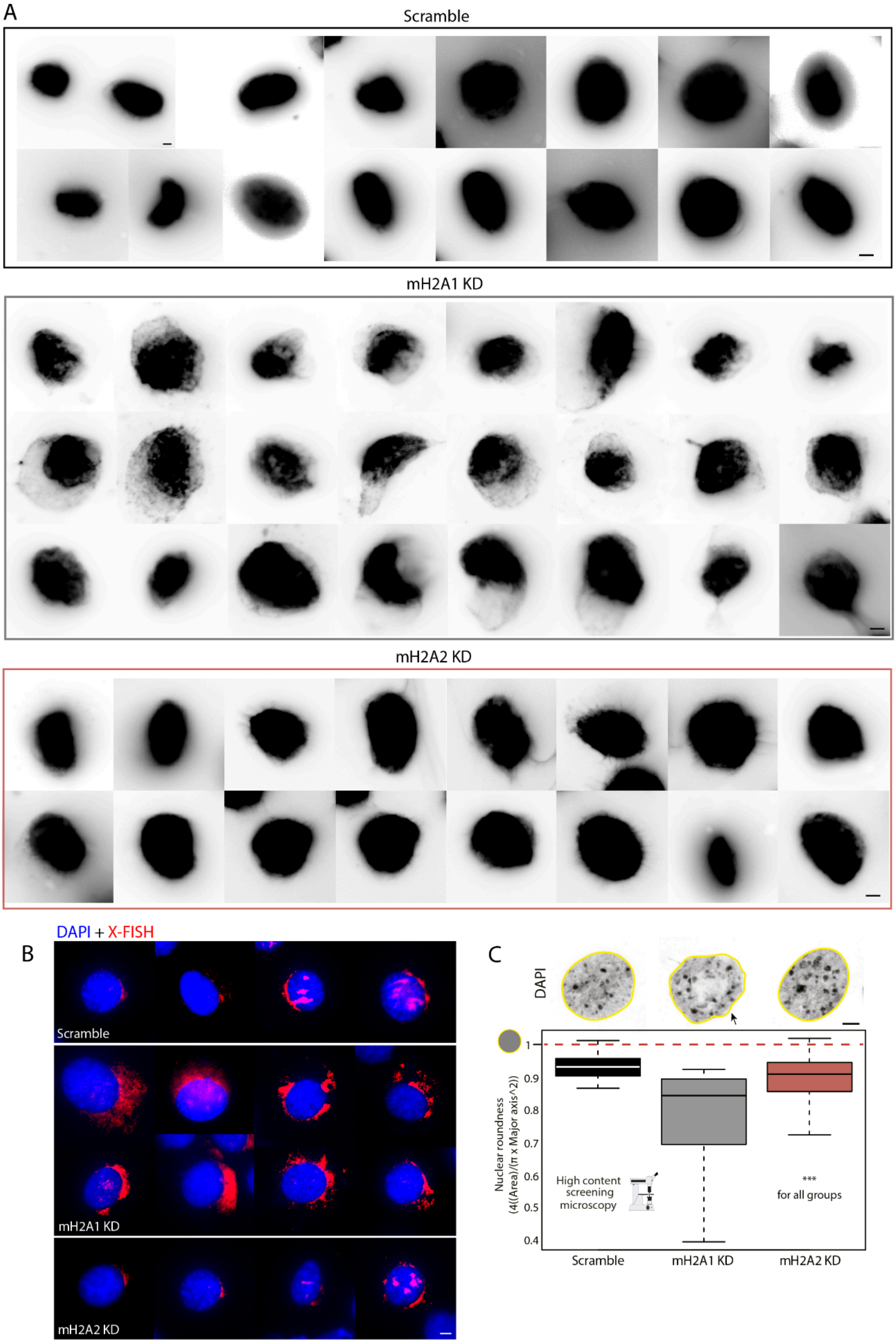
A
Scramble
mH2A1 KD
mH2A2 KD
B
DAPI + X-FISH
Scramble
mH2A1 KD
mH2A2 KD
C
DAPI
1
0.9
0.8
0.7
0.6
0.5
0.4
Nuclear roundness
(4((Area)/(π x Major axis^2))
High content screening microscopy
***
for all groups
Scramble
mH2A1 KD
mH2A2 KD

Figure S8. MacroH2A1 knockdown increases the radius of DNA halos and decreases nuclear roundness. (A) Gallery of representative images of DNA halos in C2C12 stable knockdown cell lines. An increase in the halo radius and higher irregularity were found for macroH2A1 depletion compared with control and macroH2A2 knockdown cell lines. **(B)** Gallery of representative images of X-FISH performed in DNA Halo preparations in C2C12 stable knockdown cell lines. **(C)** Nucleus roundness was analyzed by high-content microscopy using the DAPI signal for nucleus segmentation. Morphology properties such as roundness were measured using Harmony software and plotted as a boxplot. Representative images are shown on the top of the boxplot, with the nuclear periphery highlighted in yellow. N-numbers (cells): Scramble 2702, mH2A1 KD 2804, mH2A2 KD 15202. For all boxplots, the box represents 50% of the data, starting in the first quartile (25%) and ending in the third (75%). The line inside represents the median. The whiskers represent the upper and lower quartiles. Statistical significance was tested with a paired two-sample Wilcoxon test (n.s., not significant, is given for p-values ≥ 0.05; one star (*) for p-values < 0.05 and ≥ 0.005; two stars (**) is given for values < 0.005 and ≥ 0.0005; three stars (***) is given for values < 0.0005). N-numbers and p-values are shown in Supplementary Table 8 (Statistics). Scale bars: 5 µm.

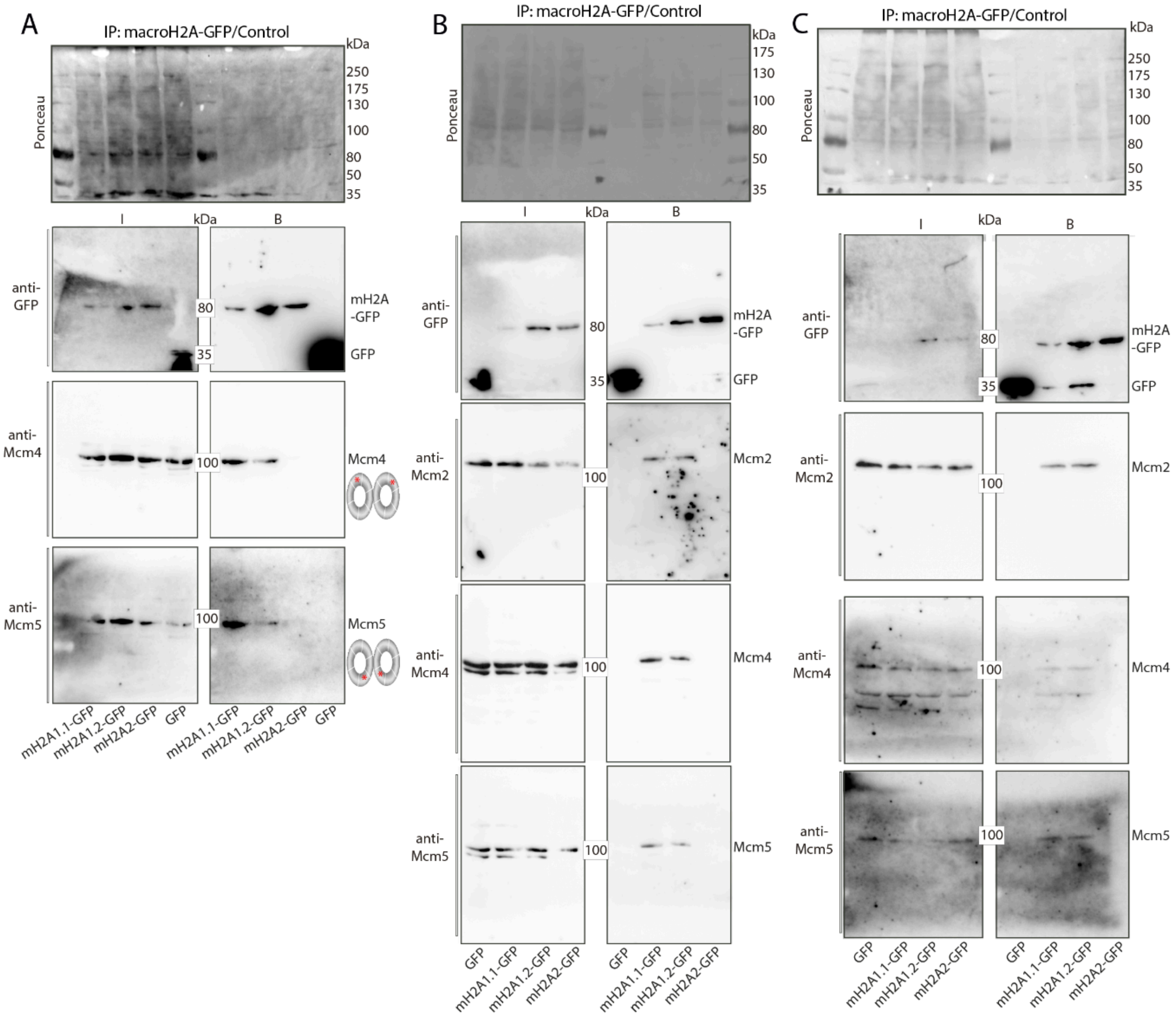

Figure S9. MacroH2A1.1 and macroH2A1.2 isoforms interact with Mcm subunits. (A) Co-immunoprecipitation experiments: C2C12 cells were transfected with EGFP or EGFP-tagged macroH2A1.1, macroH2A1.2, or macroH2A2. Cell extracts were analyzed by immunoprecipitation with

immobilized GFP-binding nanobody, followed by detection with antibodies against GFP, Mcm2 (Fig. 7G), Mcm4, and Mcm5. The cut-outs show input/bound GFP and input/bound Mcm fractions. To the right, the scheme of the DNA helicase shows the two hexamers. The position of the different Mcm subunits on the Mcm hexamers is indicated with a red star. **(B)** and **(C)** Two additional replicates of the co-immunoprecipitation of macroH2A-GFP and Mcm2/4/5: The cut-outs show input/bound GFP and input/bound Mcm2/Mcm4/Mcm5 fractions.

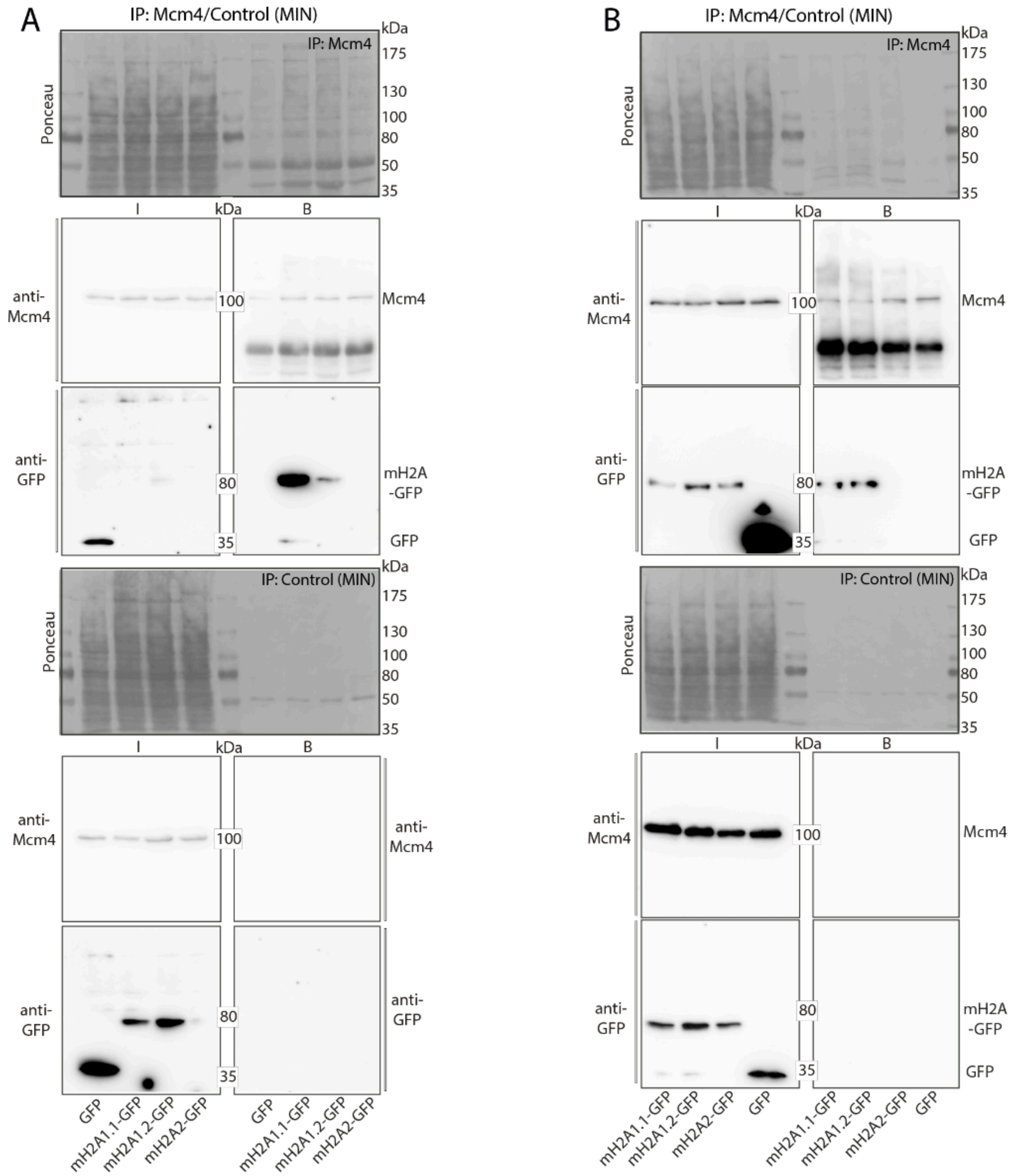

Figure S10. Immunoprecipitated Mcm can pull-down macroH2A1.1 and macroH2A1.2 isoforms, but not macroH2A2. (A) and **(B)** Mcm4-macroH2A co-immunoprecipitation in synchronized C2C12 cells: endogenous Mcm4 was immobilized using Pierce™ Protein G agarose beads preincubated with antibodies against Mcm4 or MIN (attP synthetic peptide) as negative control for immunoprecipitation (IgG control). Briefly, cells were transfected with EGFP or EGFP-tagged macroH2A1.1, macroH2A1.2, or macroH2A2, and cell extracts were analyzed by immunoprecipitation with immobilized endogenous Mcm4, followed by detection with antibodies against GFP and Mcm4. The cut-outs show input/bound GFP, input/bound Mcm4 fractions, and input/bound MIN fractions.

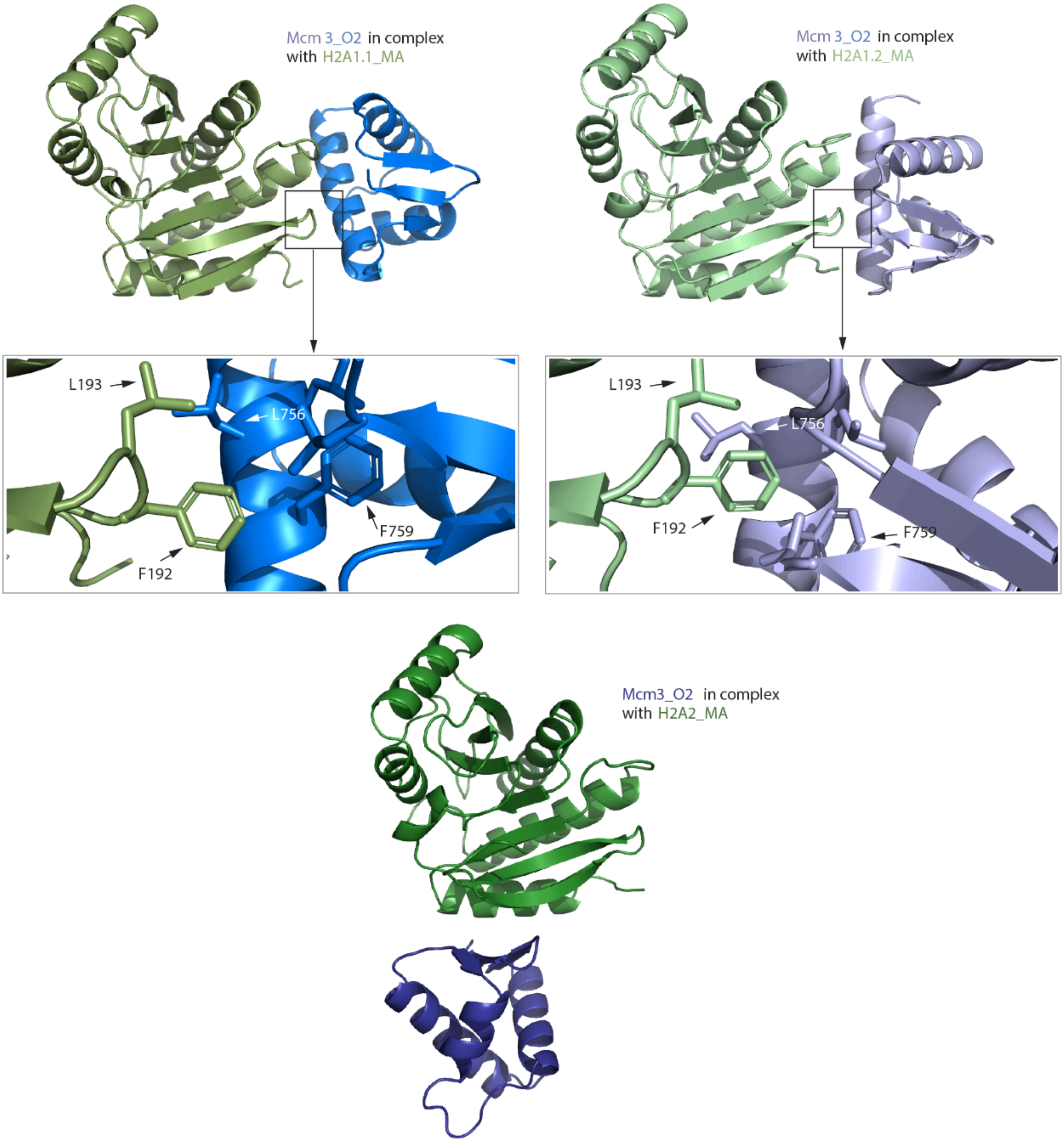

Figure S11. Structural models obtained with Alphafold for macroH2A domains paired with Mcm3_O2. MacroH2A domains are shown in green colors as in Fig. 8A-D, Mcm3_O2 is shown in different shades of blue. Zoom into the interface between the macro domains of macroH2A1.1 and 1.2 and Mcm3_O2 with key residues shown as sticks.

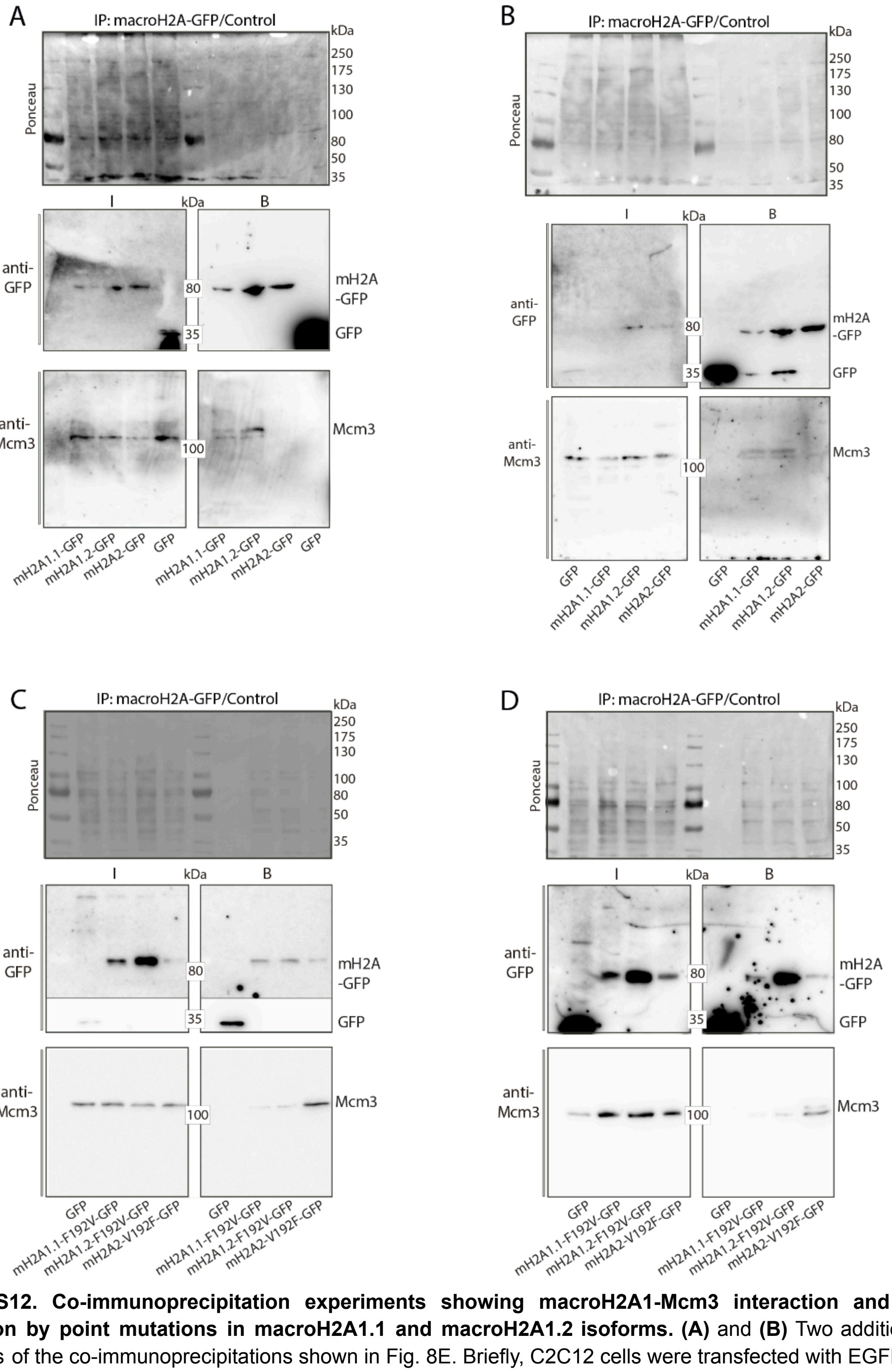

Figure S12. Co-immunoprecipitation experiments showing macroH2A1-Mcm3 interaction and its disruption by point mutations in macroH2A1.1 and macroH2A1.2 isoforms. (A) and **(B)** Two additional replicates of the co-immunoprecipitations shown in Fig. 8E. Briefly, C2C12 cells were transfected with EGFP or EGFP-tagged macroH2A1.1, macroH2A1.2, or macroH2A2. Cell extracts were analyzed by immunoprecipitation with immobilized GFP-binding nanobody, followed by detection with antibodies against GFP and Mcm3. The cut-outs show input/bound GFP and input/bound Mcm3 fractions. **(C)** and **(D)** Two additional replicates of the co-immunoprecipitations shown in Fig. 8F for macroH2A1. Co-immunoprecipitation experiments were performed as described for (A) and (B) but replacing EGFP-tagged macroH2A1.1, macroH2A1.2, or macroH2A2 for their respective mutants: macroH2A1.1-F192V, macroH2A1.2-F192V, or macroH2A2-V192F. For all co-immunoprecipitations, the cut-outs show input/bound GFP and input/bound Mcm fractions.

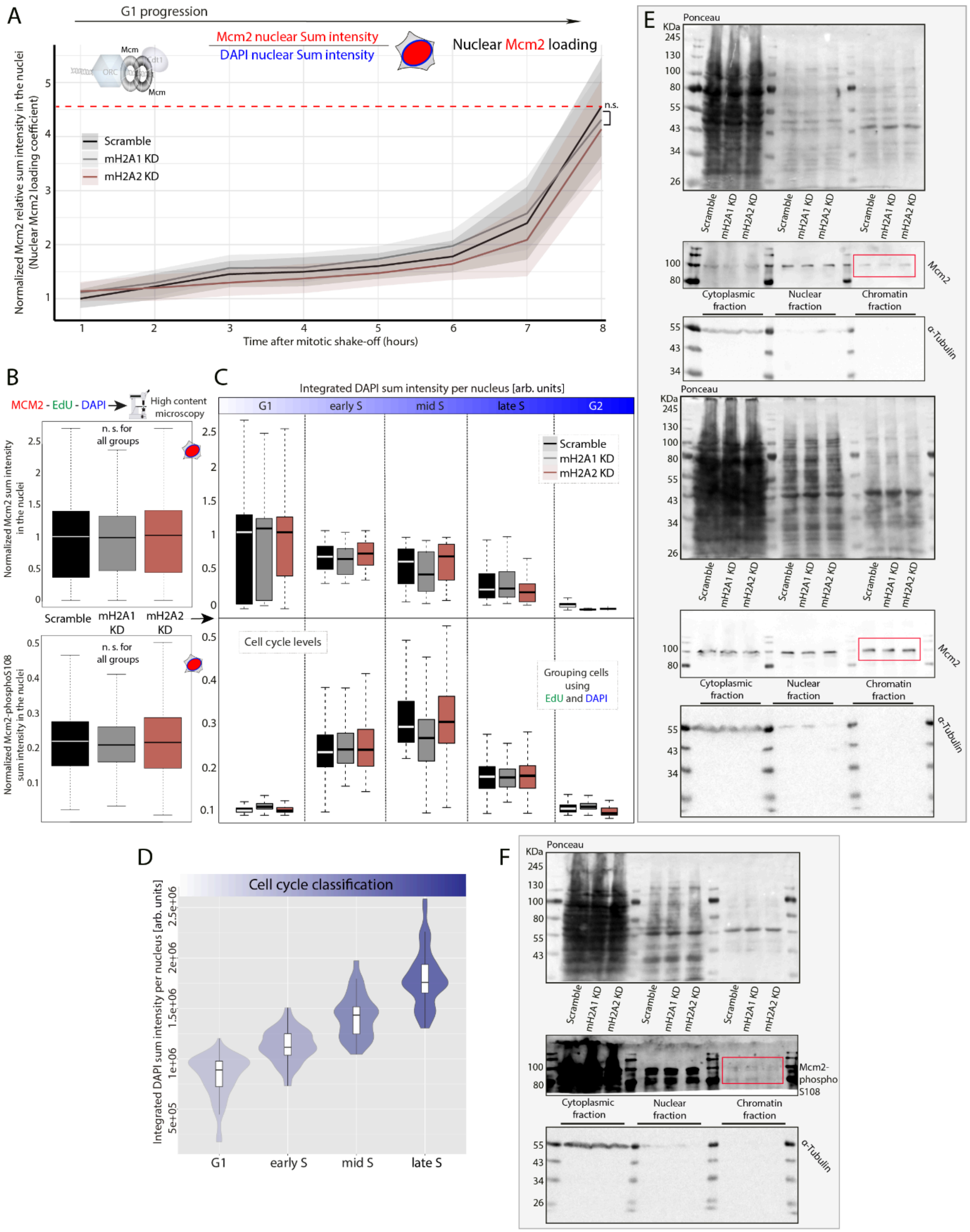
A
G1 progression
Mcm2 nuclear Sum intensity
DAPI nuclear Sum intensity
Nuclear Mcm2 loading
Mcm
ORC
Cdt1
Scramble
mH2A1 KD
mH2A2 KD
n.s.
Normalized Mcm2 relative sum intensity in the nuclei
(Nuclear Mcm2 loading coefficient)
Time after mitotic shake-off (hours)
B
MCM2 - EdU - DAPI
High content microscopy
n. s. for all groups
Normalized Mcm2 sum intensity in the nuclei
Scramble
mH2A1 KD
mH2A2 KD
Normalized Mcm2-phosphoS108 sum intensity in the nuclei
C
Integrated DAPI sum intensity per nucleus [arb. units]
G1
early S
mid S
late S
G2
Cell cycle levels
Grouping cells using EdU and DAPI
D
Cell cycle classification
Integrated DAPI sum intensity per nucleus [arb. units]
G1
early S
mid S
late S
E
Ponceau
KDa
Cytoplasmic fraction
Nuclear fraction
Chromatin fraction
Mcm2
α-Tubulin
F
Ponceau
Mcm2-phospho S108
α-Tubulin

Figure S13. Global (nuclear) levels of Mcm2 chromatin loading do not change upon macroH2A1 knockdown. (A) Mcm2 nuclear loading coefficients for each time point over G1 were calculated by dividing Mcm2 sum intensity by DAPI sum intensity. Then, nuclear loading coefficients were normalized by the average of control cells at time 1 and plotted for the three cell lines, obtaining Mcm2 G1 loading curves. N-numbers (Cells): Scramble 24-28, mH2A1 KD 15-19, mH2A2 KD 15-20. Two independent replicates. Representative images for all time points are shown in Fig. S14. **(B)** Boxplots showing nuclear levels of Mcm2 (top) and Mcm2-phosphoS108 (bottom) analyzed by high-content microscopy. Asynchronous populations of cells were incubated with EdU to label replicating DNA, then extracted and fixed as described in (2). These data were classified in different cell cycle stages and S-phase substages using DAPI and EdU sum intensities values. This classification is shown in the boxplots of **(C)**, depicting Mcm2 and Mcm2-phosphoS108 levels over the cell cycle. N-numbers (cells): Scramble 7796, mH2A1 KD 6121, mH2A2 KD 7919 (Mmc2); Scramble 5870, mH2A1 KD 6910, mH2A2 KD 6466 (Mcm2phosphoS108). Two independent replicates. **(D)** Violin plots showing DAPI sum intensity values used to classify the cells in different cell cycle stages. N-numbers (cells): 146 (G1), 76 (early), 62 (mid), 45 (late). Chromatin fractionation experiments showing Mcm2 **(E)** and Mcm2-phosphoS108 **(F)** protein levels by Western blotting in different cellular fractions (cytoplasm, nucleus, and chromatin binding fractions). Ponceau was used as a loading control, together with α-tubulin for cytoplasmic fraction. The full membrane shown for Ponceau was cut and the different parts were incubated with the different antibodies shown.

Line plots show normalized average fluorescence values, and error bands show the respective standard deviation. 95% confidence intervals are indicated in the plot. For all boxplots, the box represents 50% of the data, starting in the first quartile (25%) and ending in the third (75%). The line inside represents the median. The whiskers represent the upper and lower quartiles. The violin plot depicts the density curves of the numeric data. Statistical significance was tested with a paired two-sample Wilcoxon test (n.s., not significant, is given for p-values ≥ 0.05; one star (*) for p-values < 0.05 and ≥ 0.005; two stars (**) is given for values < 0.005 and ≥ 0.0005; three stars (***) is given for values < 0.0005). N-numbers and p-values are shown in Supplementary Table 8 (Statistics). Scale bars: 5 µm.

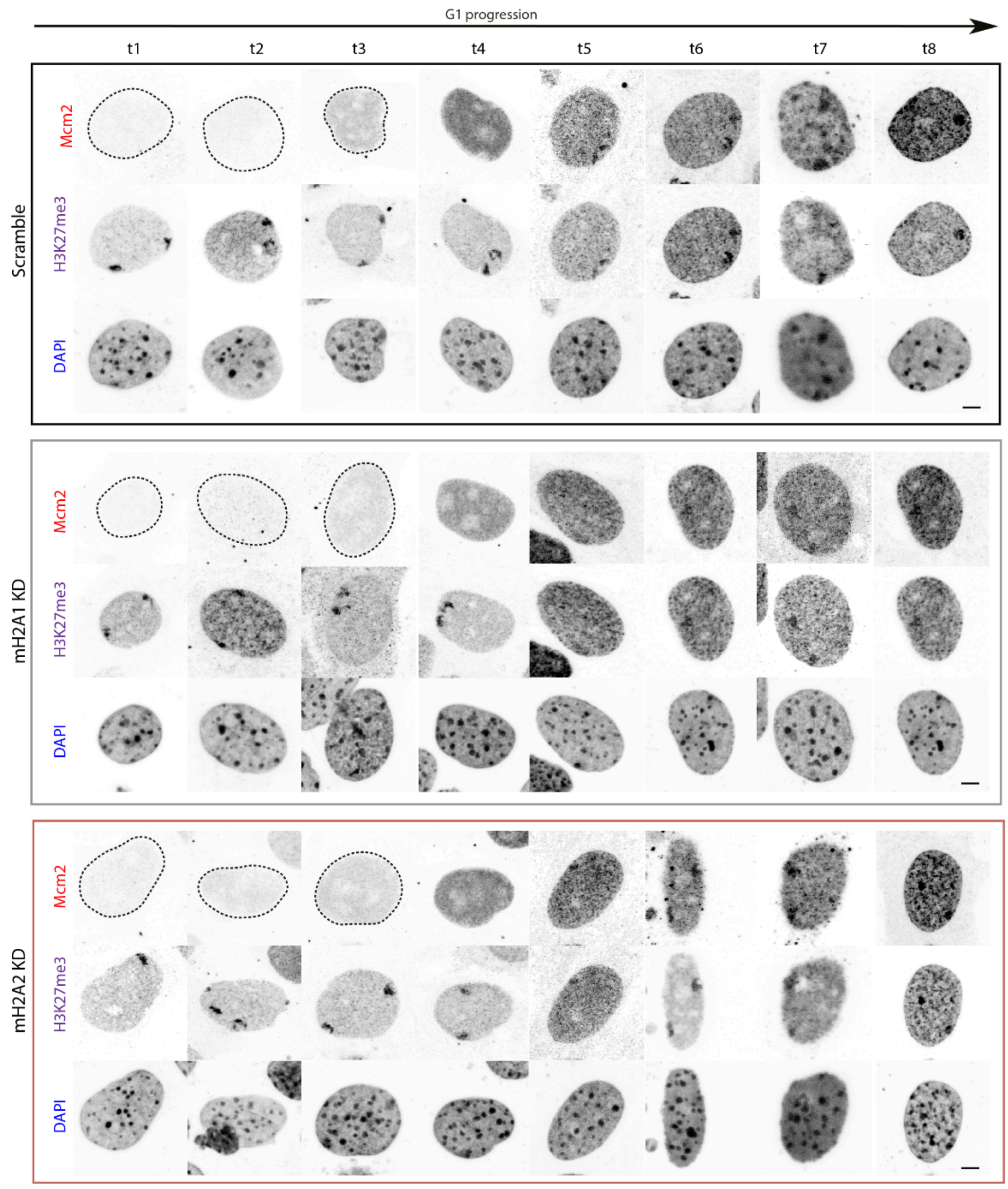

Figure S14. Representative images of G1 progression and Mcm2 loading for C2C12 knockdown cells synchronized using mitotic shake-off. A gallery of representative images for all the time points analyzed in Fig. 9 and Fig. S13A is shown. This gallery depicts the nuclear distribution and levels of the Mcm2 signal and its progressive increase over G1 (due to Mcm2 chromatin loading and assembly of pre-replication complexes). DAPI and H3K27me3 signals were used for nuclear and Xi segmentation respectively. Scale bars: 5 μm.

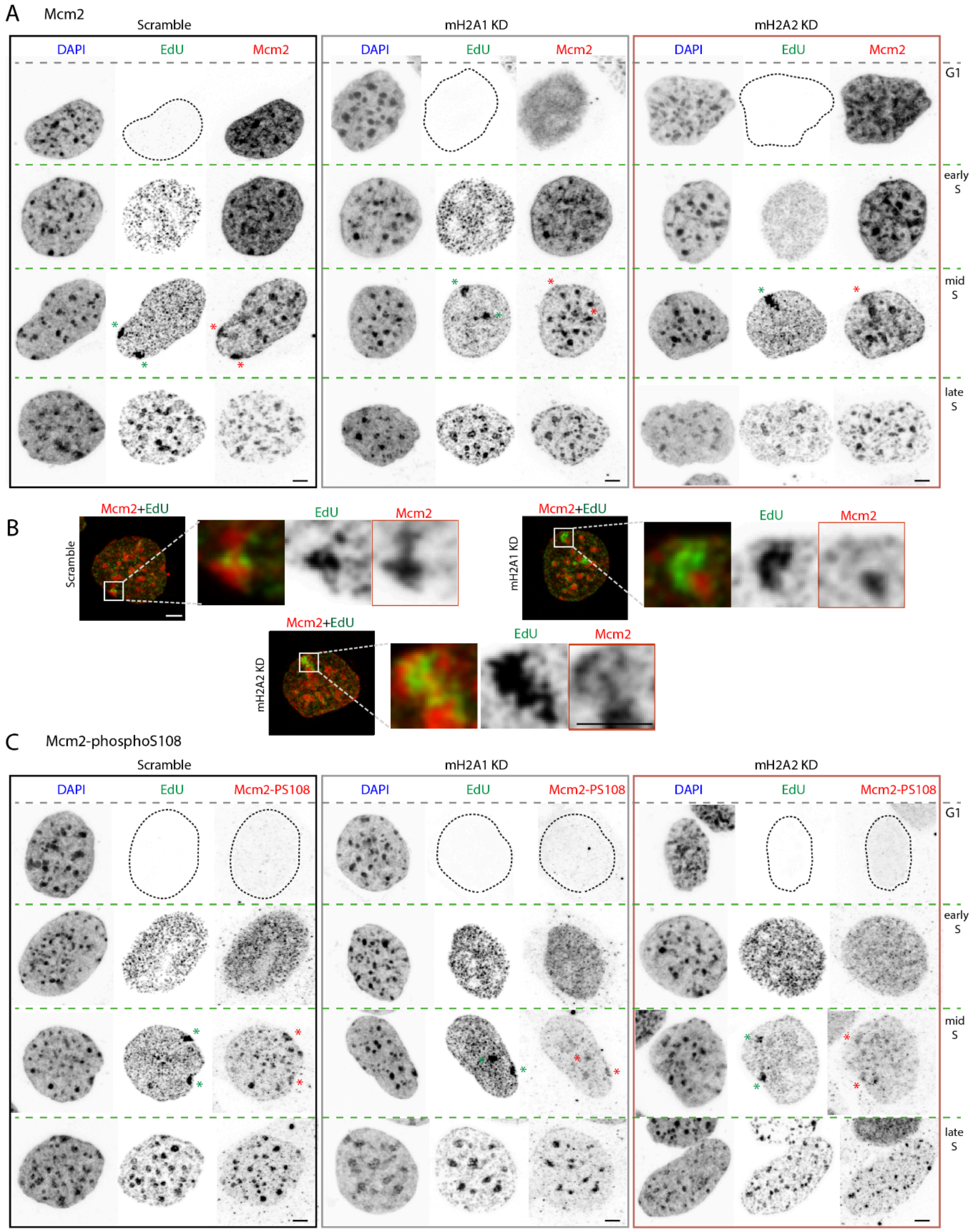

Figure S15. Representative images of Mcm2 and Mcm2-phosphoS108 in C2C12 knockdown cells without synchronization. (A) Representative confocal images for Mcm2 immunostaining with pre-extraction of the cells. Different images were selected to show different cell cycle stages (G1, early, mid, and late S-phase). **(B)** Additional examples for mid-S-phase cells when the Xi is replicated. Amplified regions (white boxes) show EdU and Mcm2 signals in the Xi. **(C)** Representative confocal images are shown as in panel (A) for Mcm2-phosphoS108 immunostaining. For all the images, green stars highlight the replicating Xi for EdU signal, and red stars for Mcm2 or Mcm2-phosphoS108. Scale bars: 5 µm.

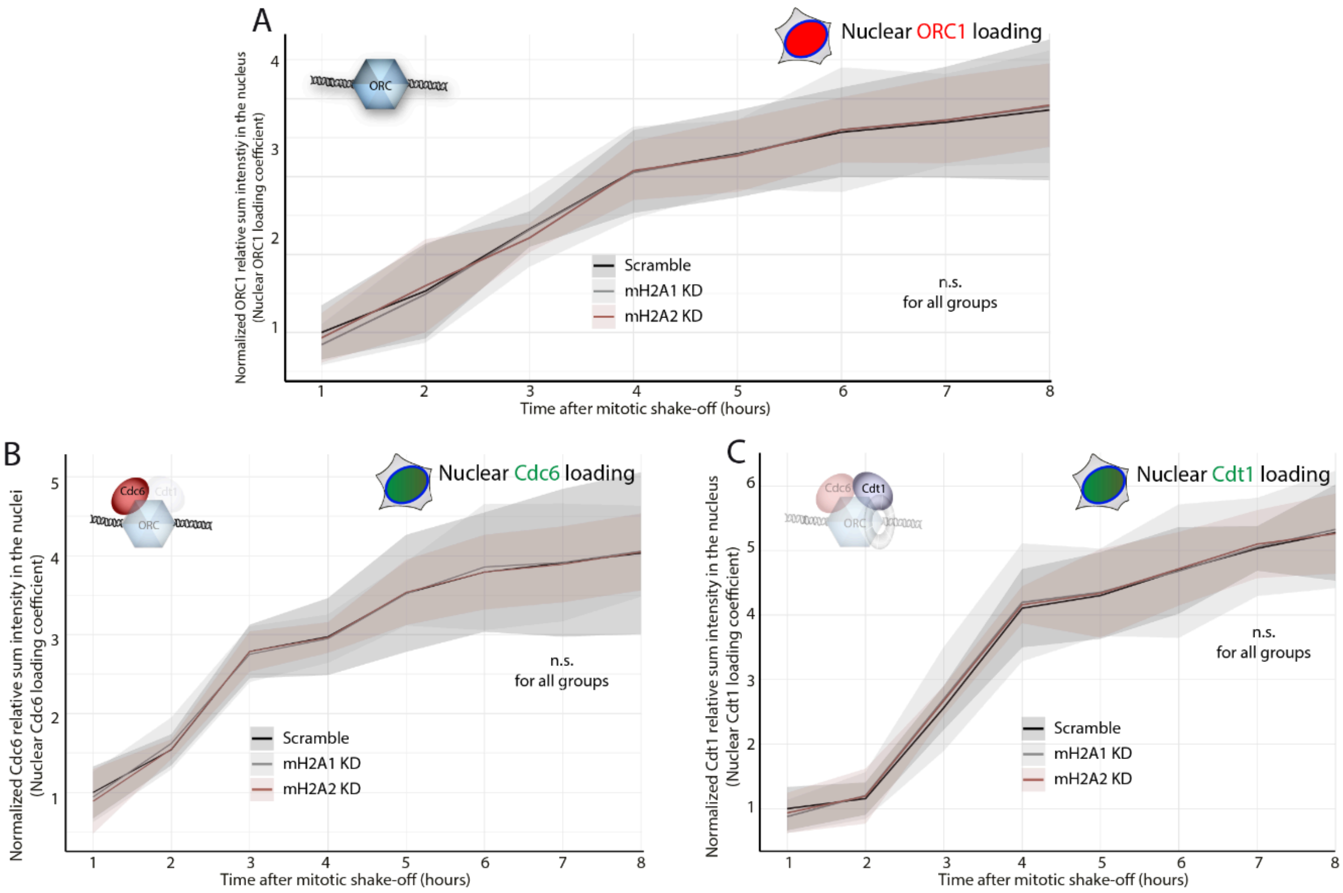

Figure S16. Global (nuclear) levels of ORC1/Cdc6/Cdt1 chromatin loading do not change upon macroH2A1 knockdown. (A) ORC1 nuclear loading coefficients for each time point over G1 were calculated by dividing ORC1 sum intensity by DAPI sum intensity. Then, nuclear loading coefficients were normalized by the average of control cells at time 1 and plotted for the three different cell lines, obtaining ORC1 G1 loading curves. N-numbers (cells): Scramble 42-49, mH2A1 KD 40-44, mH2A2 KD 42-43, four independent replicates. Representative images for all time points are shown in Fig. S17 and S18. The same experimental approach was followed for nuclear levels of Cdc6 (N-numbers (cells): Scramble 22-29, mH2A1 KD 22-24, mH2A2 KD 22-24) **(B)**, and Cdt1 (N-numbers (cells): Scramble 20-22, mH2A1 KD 19-23, mH2A2 KD 19-20) **(C)**, two independent replicates. Representative images are shown in Fig. S17 (Cdc6) and Fig. S18 (Cdt1). Line plots show normalized average fluorescence values, and error bands show the respective standard deviation. 95% confidence intervals are indicated in the plot.

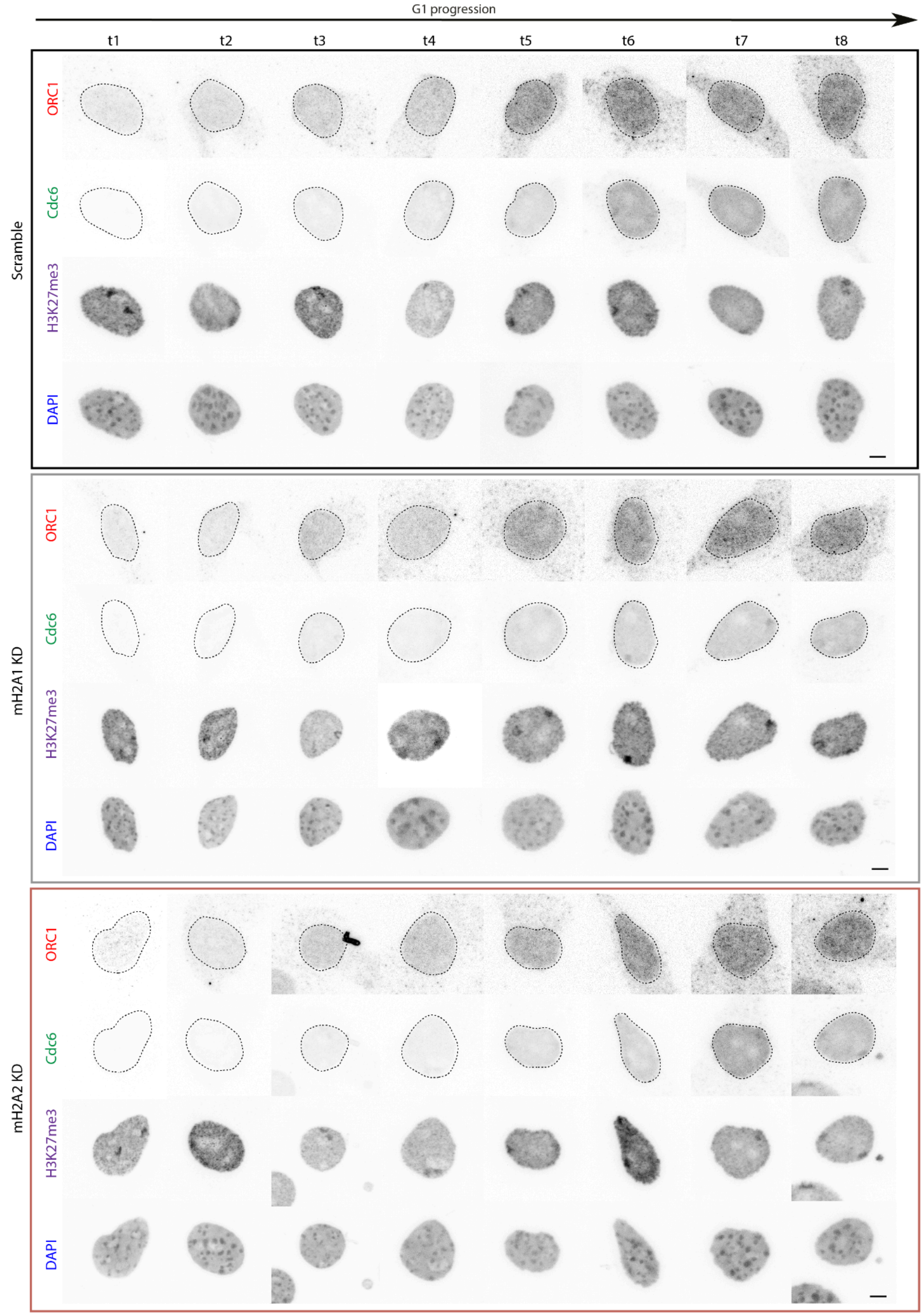

Figure S17. Representative images of G1 progression and ORC1/Cdc6 loading for C2C12 knockdown cells synchronized using mitotic shake-off. A gallery of representative images for all the time points analyzed in Fig. 9G-H and Fig. S16A-B is shown. This gallery depicts the nuclear distribution and levels of the ORC1 and Cdc6 signal and its progressive increase over G1 (due to chromatin loading and assembly of pre-replication complexes). DAPI and H3K27me3 signals were used for nuclear and Xi segmentation respectively. Scale bars: 5 µm.

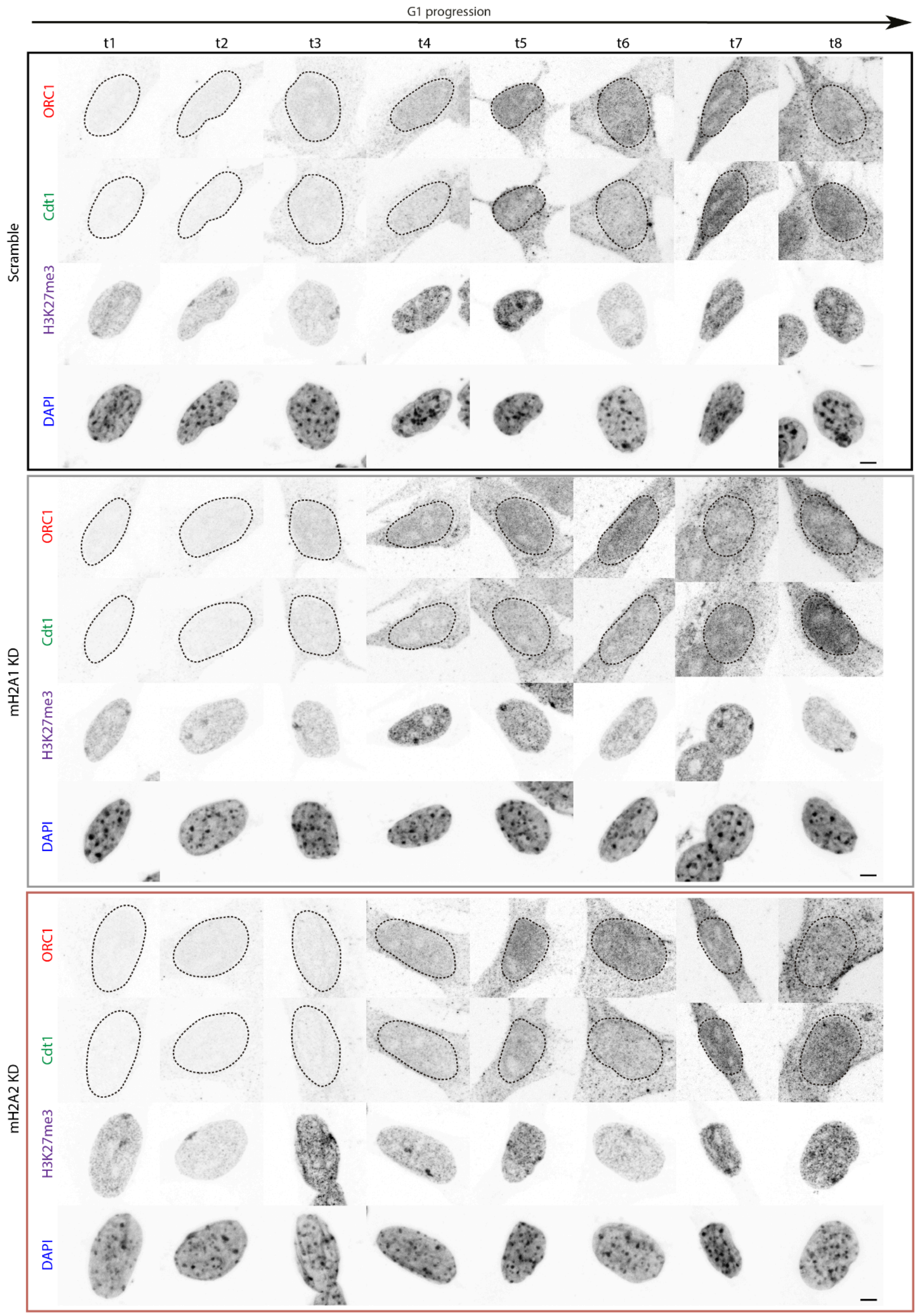

Figure S18. Representative images of G1 progression and ORC1/Cdt1 loading for C2C12 knockdown cells synchronized using mitotic shake-off. A gallery of representative images for all the time points analyzed in Fig. 9G-I and Fig. S16A and C is shown. This gallery depicts the nuclear distribution and levels of the ORC1 and Cdt1 signal and its progressive increase over G1 (due to chromatin loading and assembly of pre-replication complexes). DAPI and H3K27me3 signals were used for nuclear and Xi segmentation respectively. Scale bars: 5 μm.

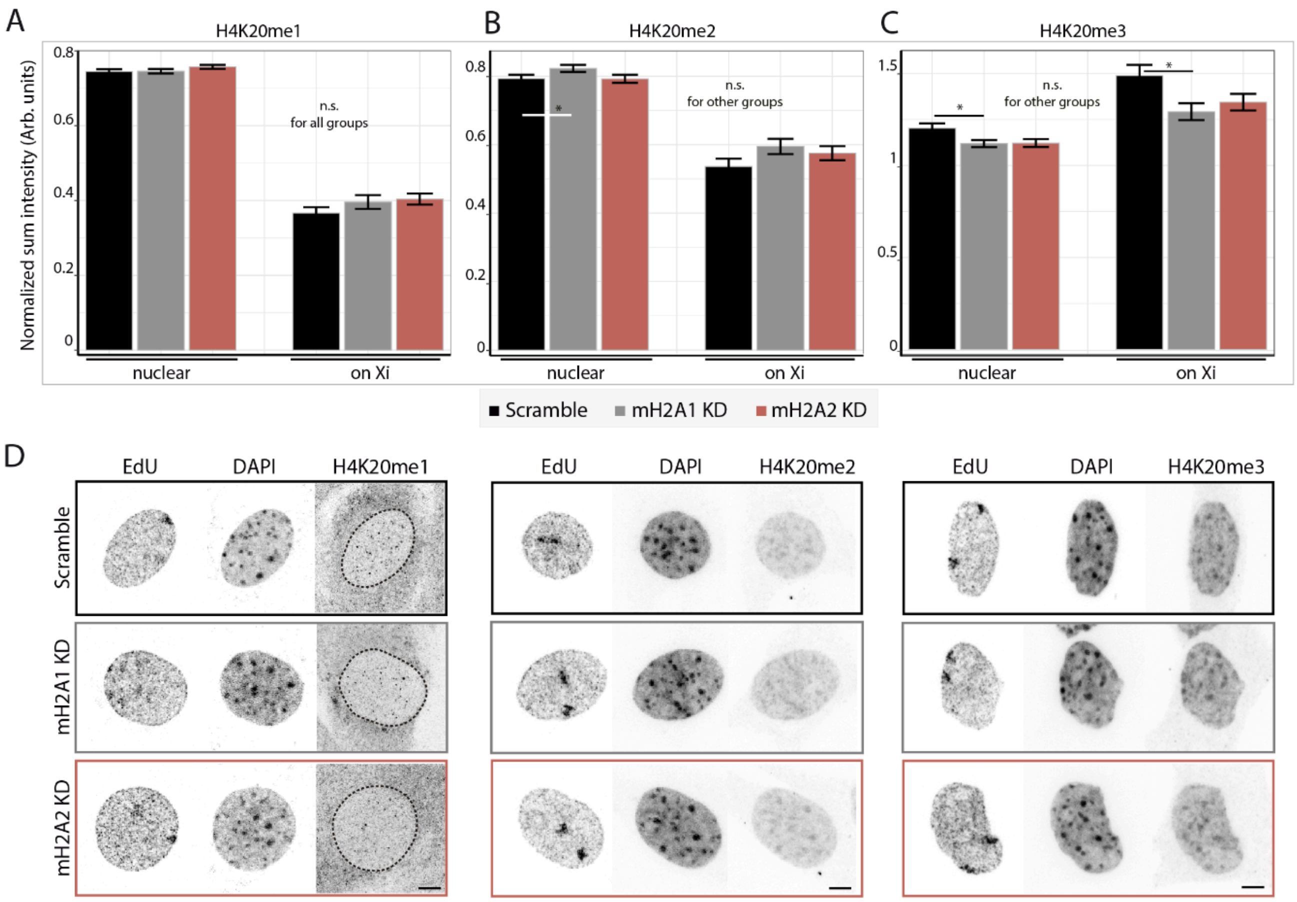

Figure S19. Quantification of H4K20 methylation levels in macroH2A depleted cells. (A) Boxplots showing the nuclear and the local levels of H4K20me1 in the Xi, followed by boxplots for H4K20me2 (B), and H4K20me3 (C). Both H4K20me1 and H4K20me2 remained unaffected in macroH2A1 and macroH2A2 deficient cells, while H4K20me3 showed differences between control and macroH2A1 knockdown cells. N-number (cells): Scramble 54, mH2A1 KD 52, mH2A2 KD 71 (H4K20me1); Scramble 75, mH2A1 KD 76, mH2A2 KD 82 (H4K20me2); Scramble 63, mH2A1 KD 72, mH2A2 KD 85 (H4K20me3). Barplots show the average value of the distribution and the whiskers represent the standard error with a 95% confidence interval. For boxplots, the box represents 50% of the data, starting in the first quartile (25%) and ending in the third (75%). The line inside represents the median. Representative images of H4K20 methylation are shown in (D). Scale bars: 5 µm.

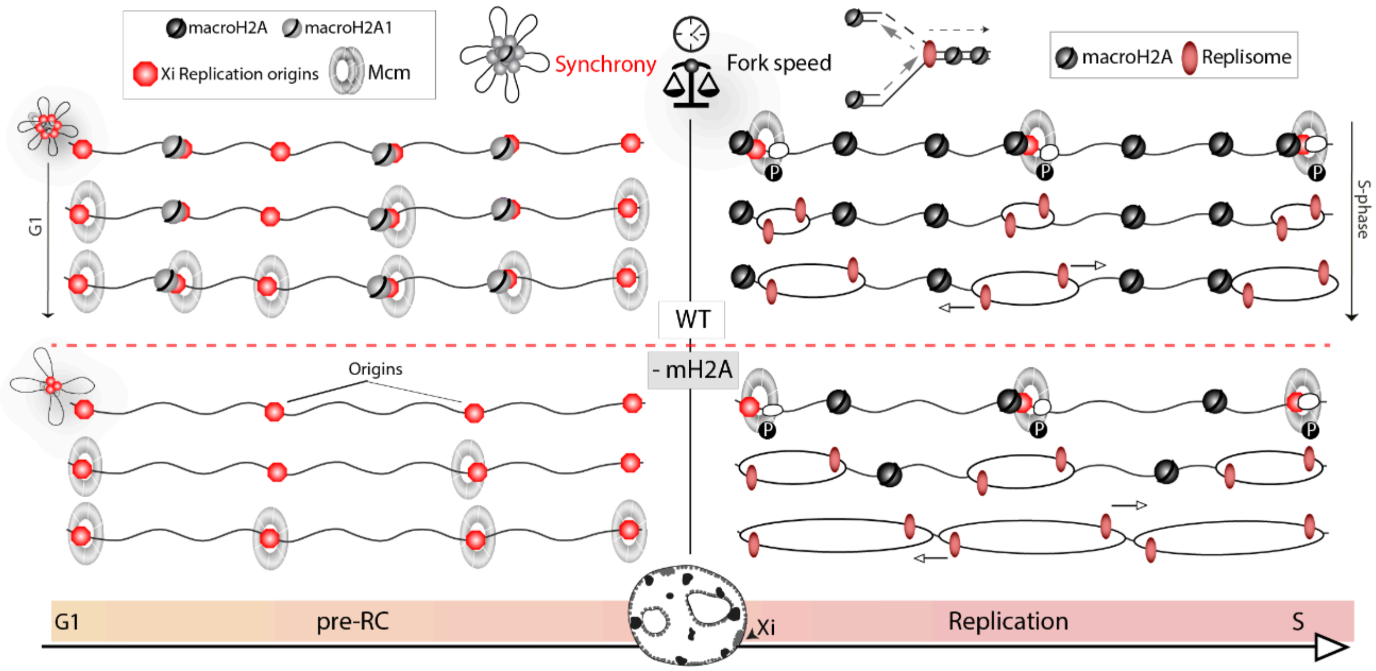

Figure S20. A full diagram of the model representing the isoform-specific role of macroH2A in Xi replication. On the left-hand side, the isoform-specific association of macroH2A1 with some of the Xi replication origins is shown to regulate the formation of chromatin loops and the accessibility of these origins to ORC and for pre-RCs assembly. This affects Mcm loading during G1, turning into less Mcm2 in the inactive X chromosome during S-phase, and consequently less Mcm2-phosphoS108. This decreases the number of active origins which negatively affects the replication rate, while macroH2A2 depletion has no effects on Xi synchronous replication. MacroH2A1 depletion also increases the transcription levels in the Xi, and it is related to advanced Xi replication timing. On the right-hand side, both macroH2A isoforms are shown to have an impact on replication fork speed: the higher stability of macroH2A-containing nucleosomes slows down the replication machinery. The average time of Xi replication is the combination of both, replication fork speed and replication synchrony, which are differently affected by macroH2A depletion. Even though both isoforms affect similar replication fork speeds, only macroH2A2 depletion reduces the time of Xi replication, since macroH2A1 negatively affects the number of active origins.

Supplementary Movies Legends

Supplementary Movies S1 – S3. Time-lapse movies of Xi replication dynamics in macroH2A-depleted cells. C2C12 stably expressing scramble shRNA (S1), shRNA against macroH2A1 (S2), shRNA against macroH2A2 (S3) transiently transfected with mRFP-PCNA (red), as a marker for active sites of DNA replication and MaSat-GFP (green) as a marker for late replicating constitutive heterochromatin. Shown are maximum intensity projections of 3D confocal stacks taken at 20-minute intervals. Cells were kept at 37 °C, 5 % CO_2, and 40 % humidity during the time-lapse. Left: mRFP-PCNA. Right: overlay of mRFP-PCNA (red) and MaSat-GFP (green). Yellow asterisks mark the Xi replication pattern. Scale bars: 5 µm.

Supplementary Movie S4. 3D rendering of C2C12 nuclei (Scramble/control cells) imaged with superresolution microscopy (3D SIM). Replicating DNA was labeled with EdU (in red), and the cell was fixed and stained with DAPI (gray). Stands out the highly synchronic Xi replication located in the nuclear periphery and visible as a replication cluster.

Movie S1 Movie S2 Movie S3 Movie S4

Supplementary Tables

Supplementary Table 1: Plasmid characteristics

Name	pc number	Fluorophore	Promoter	References
pSUPER retro puro Scr shRNA	2801	-	H1	(3) https://www.addgene.org/30520/
pSUPER retro puro macroH2A1 shRNA	2802	-	H1	(3) http://www.addgene.org/30517/
pSUPER retro puro macroH2A2 shRNA	2803	-	H1	(3) https://www.addgene.org/30518/
pMX-Tomato	2804	Tomato	H1	(3)
pEN-CAG-mRFP-PCNA	2729	mRFP	CAG	(4) https://www.addgene.org/166040/
pMasat-EGFP	1803	EGFP	CMV	(5)
pENeGFP-RPA34	0624	EGFP	CMV	(6)
pEGFP-N1	0713	EGFP	CMV	Clontech
pmacroH2A1.1-GFP	2188	EGFP	CMV	This study
pmacroH2A1.2-GFP	2189	EGFP	CMV	This study
pmacroH2A2-GFP	2191	EGFP	CMV	This study
pmacroH2A1.1-F192V-GFP	5114	EGFP	CMV	This study
pmacroH2A1.2-F192V-GFP	5115	EGFP	CMV	This study
pmacroH2A2-V192F-GFP	5116	EGFP	CMV	This study

* pc: plasmid collection number.

Supplementary Table 2: Primers and oligos

Name	Oligonucleotide sequence (5' → 3')	Application	Species	Reference
**Gapdh_fw**	CCAACATACAGGTT TCTCCAG	qPCR	*Mus musculus*	(7)
**Gapdh_rev**	CTGGAAAGCTGT GGCGTGATGG	qPCR	*Mus musculus*	
**qmH2A1_fw**	AGGATCCAAGGGAAAATTGG	qPCR	*Mus musculus*	(8)
**qmH2A1_rev**	AGGACAGTGAAGCCGTCTGT	qPCR	*Mus musculus*	
**qmH2A2_fw**	GGACCAAAGGCAAGTCAGAG	qPCR	*Mus musculus*	
**qmH2A2_rev**	TCCGAGGTGGAATTTGATGT	qPCR	*Mus musculus*	
**6MW**	CCGACTCGAGNNNNNNATGTGG	X-FISH probe generation	*Mus musculus*	(9)
**F192V_fw**	CCAAGAGCCTCGTGCTCGGCCAGA	Cloning	-	This study
**F192V_rev**	TAGAGAGGACAGTAAAGCCGTCTG	Cloning	-	This study
**V192F_fw**	AAGAGCCTTTTTCTGGGACAGAAG	Cloning	-	This study
**V192F_rev**	AGAAGACAGAATGGTGAATCCATC	Cloning	-	This study

Supplementary Table 3: Cell line characteristics

Name	Species	Type	Genotype	RRID*	Reference
C2C12	Mus musculus	Myoblasts	Wild type	CVCL_0188	(10)
C2C12 Scramble	Mus musculus	Myoblasts	Wild type	Children of CVCL_0188	This study
C2C12 mH2A1 KD	Mus musculus	Myoblasts	macroH2A1 stable knockdown	Children of CVCL_0188	This study
C2C12 mH2A2 KD	Mus musculus	Myoblasts	macroH2A2 stable knockdown	Children of CVCL_0188	This study
DF WT	Mus musculus	Skin dermal cells	Wild type	NA	(11)<br>(12)
DF mH2A1 KO	Mus musculus	Skin dermal cells	macroH2A1 knockout	NA	
DF mH2A2 KO	Mus musculus	Skin dermal cells	macroH2A2 knockout	NA	
HEK Plat-E	Human	embryonic kidney	EF1α_gag-pol_IRES_blast; EF1α_env_IRES_puro	Children of CVCL_M624	(13, 14)
MEF-1 (W9)	Mus musculus	embryonic fibroblast	Wild type	CVCL_4240	(15)

*Research Resource Identifiers (https://www.rrids.org/). DF = primary dermal fibroblasts. All cell lines used are female.

Supplementary Table 4: Primary and secondary antibodies

Reactivity	Host	Clonality	Dilution	Application	Cat / Clone #	Company / References
anti-PCNA	Mouse	Monoclonal	1:100	IF*	M0879/ PC10#	Dako, Hamburg, Germany (16)
anti-H3K27me3	Mouse	Monoclonal	1:100	IF	ab6002	Abcam, Cambridge, United Kingdom
anti-H3K27me3	Rabbit	Polyclonal	1:200	IF	07-449	Upstate, New York, USA
anti-macroH2A1	Rabbit	Polyclonal	1:500	IF/PLA, WB	07-219	Upstate, New York, USA
anti-macroH2A1	Chicken	Monoclonal	1:1,000	IF, WB	H032	(1)
anti-macroH2A1.2	Rabbit	Polyclonal	1:200	IF/PLA	#4827	Cell Signaling Technology, Inc
anti-macroH2A2	Rabbit	Polyclonal	1:500	WB	WA 3178703D	Invitrogen, USA
anti-macroH2A2	Rabbit	Polyclonal	1:400	IF/PLA	39593	Active motif, California, USA
anti-Histone H3	Rabbit	Polyclonal	1:10,000	WB	#: 2828613	Upstate, New York, USA
anti-H3K9ac	Mouse	Monoclonal	1:500	IF	61251/1B10#	Active motif, California, USA
anti-H4K8ac	Rabbit	Polyclonal	1:100	IF	06-760	Upstate, New York, USA
anti-H4K20me1	Mouse	Monoclonal	1:100	IF	sc-134221	Santa Cruz Biotechnology, Inc.
anti-H4K20me2	Rabbit	Polyclonal	1:200	IF	ab9052	Abcam, Cambridge, United Kingdom
anti-H4K20me3	Rabbit	Polyclonal	1:400	IF	ab9053	Abcam, Cambridge, United Kingdom
anti-BrdU	Rabbit	Polyclonal	1:500	IF	600-401-C29	Rockland Immunochemicals Inc., PA, USA
anti-BrdU	Rat	Monoclonal	1:100	IF	BU1/75 (ICR1)	Serotec
anti-Mcm2	Rabbit	Monoclonal	1:200, 1:8,000	IF/PLA, WB	EPR4120	Abcam, Cambridge, United Kingdom
anti-Mcm2	Mouse	Monoclonal	1:10	IF/PLA	-	Provided by R. Laskey
anti-Mcm2-phosphoS 108	Rabbit	Monoclonal	1:200	IF	EPR4121	Epitomics
anti-Mcm3	Rabbit	Polyclonal	1:1,000	WB	-	Provided by R. Knippers
anti-Mcm4	Rabbit	Polyclonal	1:1,000	WB	-	Provided by R. Knippers
anti-Mcm5	Rabbit	Polyclonal	1:1,000	WB	-	Provided by R. Knippers
anti-MIN	Rat	Monoclonal	1:500	WB	Clone 1E1	(17) (S. Bultmann)
anti-ORC1	Rat	Monoclonal	1:100	IF	sc-23887	Santa Cruz Biotechnology, Inc.
anti-Cdc6	Mouse	Monoclonal	1:100	IF	sc-9964	Santa Cruz Biotechnology, Inc.
anti-Cdt1	Mouse	Monoclonal	1:100	IF	sc-365305	Santa Cruz Biotechnology, Inc.
anti-GFP	Rat	Monoclonal	1:1,000	WB	Clone 3H9	Chromotek, Planegg-Martinsried, Germany

GFP binder	nanobody	-	1 mg/mL	coIP		(18)
anti-Tubulin (alpha)	mouse	Monoclonal	1:5,000	WB	clone DM1A	Sigma-Aldrich, USA
**Secondary antibodies:**						
anti-mouse IgG Cy3	Donkey	Polyclonal	1:800	IF	715-165-151	The Jackson Laboratory, Bar Harbor, ME, USA
anti-rabbit IgG Cy3	Donkey	Polyclonal	1:800	IF	711-165-152	The Jackson Laboratory, Bar Harbor, ME, USA
streptavidin Cy3	Goat	-	1:500	FISH	#17092758	Sigma-Aldrich, USA
anti-rat IgG AF488	Donkey	Polyclonal	1:800	IF	A11006	Invitrogen, Waltham, Massachusetts, USA
anti-mouse IgG AF488	Goat	Polyclonal	1:800	IF	2120125	Invitrogen, Waltham, Massachusetts, USA
anti-rabbit IgG AF488	Donkey	Polyclonal	1:800	IF	A11034	Invitrogen, Waltham, Massachusetts, USA
anti-rabbit IgG AF594	Goat	Polyclonal	1:500	IF	R37117	Thermo Fisher Scientific, Waltham, MA, USA
Anti-mouse IgG Cy5	Donkey	Polyclonal	1:300	IF	715-715-150	The Jackson Laboratory, Bar Harbor, ME, USA
anti-rat IgG HRP	Goat	Polyclonal	1:5,000	WB	112-035-068	The Jackson Laboratory, Bar Harbor, ME, USA
anti-rabbit IgG HRP	Goat	Polyclonal	1:10,000	WB	A-0545	Sigma-Aldrich, USA
anti-chicken IgG HRP	Rabbit	Polyclonal	1:10,000	WB	AP162P	Chemicon

*Methanol treatment required

Supplementary Table 5: Nucleotides and chemicals characteristics

Name	Application	Detection	Cat #	Company
Aphidicolin	Replisome disruption by polymerase inhibition	-	A0781-1MG	Sigma-Aldrich, St Louis, MO, USA
EdU (5-ethynyl-2’-deoxyuridine)	Labeling of nascent DNA in pulse (chase) experiments	ClickIT chemistry	E10415	Thermo Fisher Scientific, Waltham, MA, USA
BrdU (5-bromo-2’-deoxyuridine)	Labeling of nascent DNA and pulse (chase) experiments	Antibody detection	B5002	Sigma-Aldrich, St Louis, MO, USA
Thymidine	Labeling of nascent DNA in pulse (chase) experiments**	-	T9250	Sigma-Aldrich, St Louis, MO, USA
Biotin-16-dUTP	Labeling of FISH* probes	Streptavidin	-	Self-made (19)
dATP, dTTP, dCTP & dGTP	Generation of FISH probes	-	10297018	Thermo Fisher Scientific, Waltham, MA, USA

* fluorescence in situ hybridization, ** added only during the chase period in pulse-chase experiments.

Supplementary Table 6: Imaging Systems Characteristics

Microscope/ Company	Lasers/lamps	Filters (ex. & em. [nm])*	Objectives/ lenses	Detection system	Incubation system	Application
Ultra-View VoX spinning disk microscope/ PerkinElmer Life Sciences, UK	solid state diode lasers (405 nm, 488 nm, 561 nm, 640 nm)	405/488/568/640** 405: 415–475 488: 505–549 561: 580–650 640: 664–754	oil immersion 60x Plan-Apochromat (NA 1.45)	cooled 14-bit Hamamatsu® C9100-50 EMCCD	closed live-cell microscopy chamber (ACU control, Olympus) for time-lapse microscopy	time-lapse microscopy & confocal z-stack imaging
Widefield microscope Axiovert 200 /Zeiss, Germany	HBO100 Mercury lamp	488: 473-491 & 506-534 561: 550-580 & 590-650 640: 590-650 & 663-738	oil immersion 63x Plan-Apochromat (NA 1.4)	12-bit AxioCam mRM	-	DNA Halos imaging
Leica SP5 II confocal microscope /Wetzlar, Germany	405 nm diode 488 nm Argon, 561 nm DPSS, 633 nm HeNe	AOBS beam splitter	HCX PL APO 63x / 1.4-0.6 oil lambda blue & HCX PL APO 100x (NA 1.44) oil Corr CS	2 HyD Hybrid Detectors	-	confocal z-stack imaging
Amersham AI600 imager	Chemiluminescence, UV transillumination	-	-	-	-	Western blots and DNA agarose gels
Operetta high throughput imaging/ PerkinElmer Life Sciences, UK	Xenon fiber-optic light source, 300 W, 360 – 640 nm continuous spectrum LED light source for transmission mode	ex:360/400, 460/490, 560/580 em: 410/480, 500/550, 560/630	40x air objective, (NA 0.95)	14 bit Jenoptik firecamj203 Sony Chip ICX285 cooled 20°C below environment	-	high throughput, high content imaging, and image analysis

Supplementary Table 7: Genome-wide analysis of ChIP-seq

Dataset	Sample	Characteristics	Method	Cells	Webpage
**GSE142082**	GSM4219648	WT1_macroH2A1	ChIP-seq	MEF (female)	https://www.ncbi.nlm.nih.gov/geo/query/acc.cgi?acc=GSM4219648
	GSM4219649	WT1RS_macroH2A1	ChIP-seq	MEF (female)	https://www.ncbi.nlm.nih.gov/geo/query/acc.cgi?acc=GSM4219649
	GSM4219650	WT2RS_macroH2A1	ChIP-seq	MEF (female)	https://www.ncbi.nlm.nih.gov/geo/query/acc.cgi?acc=GSM4219650
	GSM4219654	Input_macroH2A1	ChIP-seq	MEF (female)	https://www.ncbi.nlm.nih.gov/geo/query/acc.cgi?acc=GSM4219654
	GSM4219651	WT6_macroH2A2	ChIP-seq	MEF (female)	https://www.ncbi.nlm.nih.gov/geo/query/acc.cgi?acc=GSM4219651
	GSM4219652	WT7_macroH2A2	ChIP-seq	MEF (female)	https://www.ncbi.nlm.nih.gov/geo/query/acc.cgi?acc=GSM4219652
	GSM4219653	WT8_macroH2A2	ChIP-seq	MEF (female)	https://www.ncbi.nlm.nih.gov/geo/query/acc.cgi?acc=GSM4219653
	GSM4219657	Input_macroH2A2	ChIP-seq	MEF (female)	https://www.ncbi.nlm.nih.gov/geo/query/acc.cgi?acc=GSM4219657
**GSE215884**	GSM6645038	anti-MacroH2A1.1	ChIP-seq	MEF (male)	https://www.ncbi.nlm.nih.gov/geo/query/acc.cgi?acc=GSM6645038
	GSM6645039	anti-MacroH2A1.2	ChIP-seq	MEF (male)	https://www.ncbi.nlm.nih.gov/geo/query/acc.cgi?acc=GSM6645039
	GSM6645040	anti-MacroH2A2	ChIP-seq	MEF (male)	https://www.ncbi.nlm.nih.gov/geo/query/acc.cgi?acc=GSM6645040

	GSM6645041	Input, MEFs	ChIP-seq	MEF (male)	https://www.ncbi.nlm.nih.gov/geo/query/acc.cgi?acc=GSM6645041
**GSE40813**	GSM1002446	Input control	ChIP-seq	MEF	https://www.ncbi.nlm.nih.gov/geo/query/acc.cgi?acc=GSM1002446
	GSM1002447	wt_macroH2A1	ChIP-seq	MEF (male)	https://www.ncbi.nlm.nih.gov/geo/query/acc.cgi?acc=GSM1002447
	GSM1064456	wt_macroH2A2	ChIP-seq	MEF (female)	https://www.ncbi.nlm.nih.gov/geo/query/acc.cgi?acc=GSM1064456

Supplementary Table 8: Statistics

Figure	Sample	n/replicates	Mean	StDev	p-Value
**1B**	S-phase (hours)<br>(1) Scramble<br>(2) mH2A1 KD<br>(3) mH2A2 KD<br>Doubling time (hours)<br>(1) Scramble<br>(2) mH2A1 KD<br>(3) mH2A2 KD	S-phase (hours)<br>673/3<br>551/3<br>449/3<br>Doubling time (hours)<br>11/3<br>11/3<br>11/3	S-phase (hours)<br>8.0<br>8.1<br>8.6<br>Doubling time (hours)<br>14.9<br>15.0<br>14.8	S-phase (hours)<br>1.3<br>1.2<br>0.6<br>Doubling time (hours)<br>1.3<br>2.2<br>1.5	S-phase (hours)<br>(1-2) 0.7<br>(1-3) 0.4<br>(2-3) 0.4<br>Doubling time (hours)<br>(1-2) 0.6522<br>(1-3) 0.847<br>(2-3) 0.7969
**1C**	(1) Scramble<br>(2) mH2A1 KD<br>(3) mH2A2 KD<br><br>(4) WT<br>(5) mH2A1 KO<br>(6) mH2A2 KO	>1524 cells/9<br>>1492 cells/7<br>>1578 cells/9<br><br>>500/4<br>>500/3<br>>500/3	20.09625909<br>21.51892678<br>11.17755586<br><br>18.55<br>21.05<br>11.10	4.43<br>6.33<br>4.05<br><br>4.8<br>2.9<br>2.2	(1-2) 0.6806<br>(1-3) 0.001851<br>(2-3) 0.007867<br><br>(4-6) 0.001851<br>(4-5) 0.6806<br>(5-6) 0.007867
**1F**	S-phase (hours)<br>(1) Scramble<br>(2) mH2A1 KD<br>(3) mH2A2 KD<br>Xi replication (min)<br>1) Scramble<br>(2) mH2A1 KD<br>(3) mH2A2 KD	S-phase (hours)<br>4/2<br>6/2<br>6/2<br>Xi replication (min)<br>32/2<br>26/2<br>18/2	S-phase (hours)<br>6.7<br>6.4<br>7.1<br>Xi replication (min)<br>79.4<br>80.0<br>52.2	S-phase (hours)<br>0.49<br>0.76<br>0.95<br>Xi replication (min)<br>17.2<br>15.0<br>17.0	S-phase (hours)<br>(1-2) 0.5147<br>(1-3) 0.8264<br>(2-3) 0.4159<br>Xi replication (min)<br>(1-2) 0.438<br>(1-3) 3.208e-06<br>(2-3) 7.655e-06
**2B**	(1) Scramble<br>(2) mH2A1 KD<br>(3) mH2A2 KD<br><br>(4) WT<br>(5) mH2A1 KO<br>(6) mH2A2 KO	174/8<br>150/8<br>174/8<br><br>22/2<br>32/2<br>26/1	1.012894218<br>1.436361337<br>1.374476542<br><br>1.00<br>1.57<br>1.46	0.023875453<br>0.121865881<br>0.111225433<br><br>0.30<br>0.33<br>0.48	(1-2) 0.0007781<br>(1-3) 0.0007853<br>(2-3) 0.3181<br><br>(1-2) 1.19329E-07<br>(1-3) 0.006819009<br>(2-3) 0.001548167
**2C**	(1) Scramble<br>(2) mH2A1 KD<br>(3) mH2A2 KD	28/2<br>23/2<br>26/2	1<br>1.065057649<br>1.050032846	0.145531393<br>0.130266667<br>0.221926091	(1-2) 0.1236<br>(1-3) 0.4545<br>(2-3) 0.4681
**2E**	(1) Scramble<br>(2) mH2A1 KD<br>(3) mH2A2 KD<br><br>t (min): 0, 1, 5, 10, 15, 20, 25, 30	18/2<br>11/2<br>12/2	(1) | (2) | (3)<br>1 | 1 | 1<br>1.106 | 1.159 | 1.463<br>1.596 | 1.921 | 2.020<br>1.866 | 2.238 | 2.137<br>1.983 | 2.461 | 2.392<br>2.149 | 2.593 | 2.564<br>2.148 | 2.784 | 2.665<br>2.194 | 2.847 | 2.793	(1) | (2) | (3)<br>0.35 | 0.54 | 0.31<br>0.45 | 0.61 | 0.20<br>0.46 | 0.63 | 0.27<br>0.48 | 0.59 | 0.24<br>0.46 | 0.62 | 0.22<br>0.47 | 0.59 | 0.19<br>0.43 | 0.67 | 0.17<br>0.43 | 0.79 | 0.21	t (30 min):<br><br>(1-2) 0.026241742<br>(1-3) 0.035841654<br><br>t (1 min):<br><br>(1-2) 0.730853045<br>(1-3) 0.645645134
**3B**	(1) Scramble<br>(2) mH2A1 KD<br>(3) mH2A2 KD<br><br>(4) WT<br>(5) mH2A1 KO<br>(6) mH2A2 KO	18/3<br>25/3<br>22/3<br><br>4/1<br>6/1<br>10/1	136.1<br>111.5<br>141.5<br><br>135.25<br>114<br>132.25	63.7<br>42.3<br>61.2<br><br>28.99<br>11.34<br>3.59	(1-2) 0.00026<br>(2-3) 0.00003<br>(1-3) 0.8371<br>(1-2) 0.02086<br>(2-3) 0.01164<br>(4-6) 0.4857<br>(4-5) 0.03429<br>(5-6) 0.02857

**3D**	H3K27me3-Xi<br>(1) Scramble<br>(2) mH2A1 KD<br>(3) mH2A2 KD<br>X-FISH<br>(1) Scramble<br>(2) mH2A1 KD<br>(3) mH2A2 KD	H3K27me3-Xi<br>34/2<br>30/2<br>23/2<br>X-FISH<br>14/1<br>7/1<br>8/1	H3K27me3-Xi<br>138.25<br>91.7<br>137.26<br>X-FISH<br>139.3571429<br>93.42857143<br>140.875	H3K27me3-Xi<br>21.90<br>23.00<br>19.7<br>X-FISH<br>19.77024626<br>19.73877016<br>15.3570598	H3K27me3-Xi<br>(1-2) 1.075e-08<br>(1-3) 0.7387<br>(2-3) 3.949e-072<br>X-FISH<br>(1-2) 0.0008313<br>(1-3) 0.937<br>(2-3) 0.0021
**4A**	Xi replication start<br>(1) Scramble<br>(2) mH2A1 KD<br>(3) mH2A2 KD<br>Xi replication time<br>(1) Scramble<br>(2) mH2A1 KD<br>(3) mH2A2 KD<br>Total S-phase duration<br>1) Scramble<br>(2) mH2A1 KD<br>(3) mH2A2 KD	Xi replication start<br>33/3<br>32/3<br>27/3<br>Xi replication time<br>16/3<br>23/3<br>25/3<br>Total S-phase duration<br>3/3<br>3/3<br>3/3	Xi replication start<br>210.3030303<br>180<br>215.5555556<br>Xi replication time<br>78.75<br>80<br>52.8<br>Total S-phase duration<br>430.2481063<br>431.8720611<br>431.7026701	Xi replication start<br>34.32244605<br>37.33026101<br>23.75084344<br>Xi replication time<br>28.72281323<br>17.05605731<br>15.14375559<br>Total S-phase duration<br>1.020426976<br>1.093889572<br>1.89788718	Xi replication start<br>(1-2) 0.0002452<br>(1-3) 0.1962<br>(2-3) 2.436e-05<br>Xi replication time<br>(1-2) 0.0002452<br>(1-3) 6.429e-05<br>(2-3) 2.436e-05
**5C**	DNA halo radius<br>(1) Scramble<br>(2) mH2A1 KD<br>(3) mH2A2 KD<br>DNA halo circularity<br>(1) Scramble<br>(2) mH2A1 KD<br>(3) mH2A2 KD	DNA halo radius<br>56/3<br>60/3<br>61/3<br>DNA halo circularity<br>56/3<br>60/3<br>61/3	DNA halo radius<br>2.07284760201<br>4.292315175525<br>2.37633982722<br>DNA halo circularity<br>0.788136955<br>0.632464286<br>0.760535714	DNA halo radius<br>0.477615865755<br>0.76446488172<br>0.64879150884<br>DNA halo circularity<br>0.106058534<br>0.107477292<br>0.128177532	DNA halo radius<br>(1-2) 4.814e-13<br>(1-3) 0.1428<br>(2-3) 3.177e-12<br>DNA halo circularity<br>(1-2) 0.00135<br>(1-3) 0.02156<br>(2-3) 0.001561
**5E**	(1) Scramble<br>(2) mH2A1 KD<br>(3) mH2A2 KD	81/2 Position:<br>82/2 X0<br>75/2 X1<br>X2.5<br>X5<br>X7.5<br>X10<br>X12.5<br>X15<br>X17.5<br>X20	(1) (2) (3)<br>0.80 0.74 0.88<br>0.82 0.88 0.71<br>0.49 0.78 0.41<br>0.27 0.63 0.28<br>0.24 0.49 0.24<br>0.19 0.39 0.21<br>0.17 0.31 0.19<br>0.16 0.25 0.18<br>0.16 0.19 0.18<br>0.16 0.15 0.17	(1) (2) (3)<br>0.13 0.14 0.09<br>0.13 0.10 0.16<br>0.17 0.12 0.14<br>0.08 0.20 0.11<br>0.06 0.22 0.07<br>0.04 0.20 0.06<br>0.04 0.16 0.06<br>0.04 0.14 0.05<br>0.04 0.11 0.04<br>0.05 0.07 0.04	X1:<br>(1-2) 0.003433<br>(2-3) 1.301e-11<br>(1-3) 5.617e-06<br>X2.5:<br>(1-2) 2.2e-16<br>(1-3) 0.000487<br>(2-3) 2.2e-16<br>X5:<br>(1-2) 2.2e-16<br>(1-3) 0.688<br>(2-3) 2.2e-16<br>X10:<br>(1-2) 6.073e-07<br>(1-3) 0.3844<br>(2-3) 3.878e-07<br>X20:<br>(1-2) 0.04648<br>(1-3) 0.5287<br>(2-3) 0.02929
**5F**	(1) Scramble<br>(2) mH2A1 KD<br>(3) mH2A2 KD	64/2<br>58/2<br>62/2	0.02024605<br>0.03220907<br>0.01899243	0.00375884<br>0.00676063<br>0.00468727	(1-2) 2.2e-16<br>(1-3) 0.1036<br>(2-3) 2.2e-16
**7C**	(1) Scramble<br>(2) mH2A1 KD<br>(3) mH2A2 KD	27/2<br>34/2<br>33/2	19.81481481<br>2<br>18.24242424	10.83809889<br>1.255291829<br>7.750122189	(1-2) 2.055e-11<br>(1-3) 0.8175<br>(2-3) 1.707e-12
**7D**	(1) Scramble - nuclear<br>(1´) Scramble - Xi<br>(2) mH2A1 KD - nuclear<br>(2´) mH2A1 KD - Xi<br>(3) mH2A2 KD - nuclear<br>(3´) mH2A2 KD - Xi	21/2<br><br>23/2<br><br>23/2	1<br>2.31370574<br>0.129932697<br>0.224915113<br>0.966197151<br>2.098745666	0.533732885<br>1.38606246<br>0.105993577<br>0.757809265<br>0.218106112<br>1.058758633	(1-2) 1.915e-08<br>(1-2´) 5.636e-07<br>(1´-3) 0.0009178<br>(1´-3´) 0.2304<br>(2-2´) 1.351e-06<br>(3-3´) 4.857e-06
**7E**	(1) Neg. control (Scramble)<br>(1´) Neg. control (MEF)<br>(2) mH2A1 - Scramble<br>(2´) mH2A1 - MEF	(1) 7356/2<br>(1´) 3980/1<br>(2) 11707/2<br>(2´) 2816/1<br>(3) 4264/2	1.101563562<br>0.97788389<br>15.05296429<br>17.069627<br>14.89469981	0.882483887<br>0.840475245<br>13.18206606<br>5.05268452<br>6.859121913	(1-1´) 1.112e-13<br>(2-2´) 2.2e-16<br>(3-3´) 1.121e-09<br>(4-4´) 2.2e-16<br>(2´-3´) 2.2e-16

	(3) mH2A1.2 - Scramble<br>(3´) mH2A1.2 - MEF<br>(4) mH2A2 - Scramble<br>(4´) mH2A2 - MEF		(3´) 4761/2<br>(4) 11617/2<br>(4´) 6710/1	12.94021739<br>3.727875344<br>3.546430168			6.433199987<br>2.305187285<br>2.610206191			(1-3) 2.2e-16<br>(2´-4´) 2.2e-16<br>(2-4) 2.2e-16<br>(2-4) 2.2e-16<br>(1-4) 2.2e-16
**9C**	(1) Scramble<br>(2) mH2A1 KD<br>(3) mH2A2 KD	t (h)<br>1<br>2<br>3<br>4<br>5<br>6<br>7<br>8	24-28/2<br>15-19/2<br>15-20/2	(1)<br>1<br>1.26<br>1.49<br>2.19<br>2.36<br>2.76<br>4.32<br>4.44	(2)<br>0.95<br>1.12<br>1.34<br>1.44<br>1.87<br>2.05<br>2.58<br>2.77	(3)<br>0.91<br>1.35<br>1.62<br>2.13<br>2.42<br>3.35<br>4.09<br>4.24	(1)<br>0.27<br>0.19<br>0.20<br>0.40<br>0.53<br>0.74<br>1.12<br>1.96	(2)<br>0.29<br>0.35<br>0.30<br>0.25<br>0.56<br>0.42<br>0.88<br>0.81	(3)<br>0.34<br>0.13<br>0.26<br>0.30<br>0.70<br>0.51<br>0.76<br>1.24	t (4 hours)<br>(1-2) 0.02546<br>(1-3) 0.774<br>(2-3) 0.0090043<br><br>t (8 hours)<br>(1-2) 0.0003695<br>(1-3) 0.9563<br>(2-3) 0.000227
**9E**	Mcm2<br>(1) Scramble<br>(2) mH2A1 KD<br>(3) mH2A2 KD<br>Mcm2-phosphoS108<br>(1) Scramble<br>(2) mH2A1 KD<br>(3) mH2A2 KD		Mcm2<br>13/<br>16/<br>15/<br>Mcm2-phosphoS108<br>20/<br>20/<br>19/	Mcm2<br>1.5346112<br>1.14465353<br>1.51053619<br>Mcm2-phosphoS108<br>0.7883<br>0.4658<br>0.7946			Mcm2<br>0.2352<br>0.2746<br>0.2379<br>Mcm2-phosphoS108<br>0.2059<br>0.1247<br>0.3203			Mcm2<br>(1-2) 0.001038<br>(1-3) 0.7856<br>(2-3) 0.0006351<br>Mcm2-phosphoS108<br>(1-2) 6.178e-07<br>(1-3) 0.7895<br>(2-3) 0.0001573
**9G**	(1) Scramble<br>(2) mH2A1 KD<br>(3) mH2A2 KD	t (h)<br>1<br>2<br>3<br>4<br>5<br>6<br>7<br>8	42-49/4<br>39-49/4<br>41-44/4	(1)<br>1<br>1.53<br>2.08<br>2.57<br>2.90<br>3.16<br>3.40<br>3.68	(2)<br>0.92<br>1.50<br>2.05<br>2.64<br>2.88<br>3.18<br>3.37<br>3.72	(3)<br>0.97<br>1.56<br>2.01<br>2.66<br>2.82<br>3.19<br>3.33<br>3.61	(1)<br>0.28<br>0.69<br>0.83<br>0.74<br>0.76<br>0.60<br>0.73<br>0.41	(2)<br>0.36<br>0.42<br>0.80<br>0.65<br>0.40<br>0.44<br>0.44<br>0.91	(3)<br>0.27<br>0.29<br>0.60<br>0.48<br>0.40<br>0.42<br>0.30<br>0.52	t (4 hours)<br>(1-2) 0.3863<br>(1-3) 0.1535<br>(2-3) 0.8958<br><br>t (8 hours)<br>(1-2) 0.1271<br>(1-3) 0.211<br>(2-3) 0.8065
**9H**	(1) Scramble<br>(2) mH2A1 KD<br>(3) mH2A2 KD	t (h)<br>1<br>2<br>3<br>4<br>5<br>6<br>7<br>8	22-29/2<br>22-24/2<br>22-24/2	1<br>1.77<br>2.67<br>3.44<br>3.82<br>4.12<br>4.61<br>4.70	0.81<br>1.80<br>2.68<br>3.47<br>3.81<br>4.09<br>4.60<br>4.68	0.96<br>1.79<br>2.66<br>3.43<br>3.84<br>4.09<br>4.61<br>4.68	0.46<br>0.66<br>0.89<br>1.04<br>1.14<br>0.89<br>0.93<br>0.84	0.42<br>0.54<br>0.70<br>0.55<br>1.02<br>0.48<br>0.60<br>0.82	0.29<br>0.32<br>0.32<br>0.44<br>0.54<br>0.56<br>0.28<br>0.47	t (4 hours)<br>(1-2) 0.5937<br>(1-3) 0.5062<br>(2-3) 0.8782<br><br>t (8 hours)<br>(1-2) 0.9506<br>(1-3) 0.9418<br>(2-3) 0.7121
**9I**	(1) Scramble<br>(2) mH2A1 KD<br>(3) mH2A2 KD	t (h)<br>1<br>2<br>3<br>4<br>5<br>6<br>7<br>8	17-21/2<br>19-23/2<br>19-20/2	1<br>1.10<br>1.21<br>2.17<br>2.64<br>2.91<br>3.09<br>3.59	0.94<br>1.08<br>1.20<br>2.12<br>2.61<br>2.88<br>3.09<br>3.67	0.92<br>1.12<br>1.19<br>2.19<br>2.63<br>2.90<br>3.06<br>3.73	0.24<br>0.36<br>0.21<br>0.41<br>0.37<br>0.22<br>0.31<br>0.56	0.22<br>0.15<br>0.26<br>0.67<br>0.38<br>0.61<br>0.45<br>0.87	0.23<br>0.19<br>0.17<br>0.18<br>0.26<br>0.45<br>0.30<br>0.52	t (4 hours)<br>(1-2) 0.4777<br>(1-3) 0.2012<br>(2-3) 0.2211<br><br>t (8 hours)<br>(1-2) 0.7994<br>(1-3) 0.698<br>(2-3) 0.5648
**S1B**	macroH2A1 levels<br>(1) Scramble<br>(2) mH2A1 KD<br>(3) mH2A2 KD<br>macroH2A2 levels<br>(1) Scramble<br>(2) mH2A1 KD<br>(3) mH2A2 KD		macroH2A1 levels<br>NA/7<br>NA/7<br>NA/5<br>macroH2A2 levels<br>NA/6<br>NA/7<br>NA/6	macroH2A1 levels<br>1<br>0.2380<br>0.9733<br>macroH2A2 levels<br>1<br>1.0831<br>0.1870			macroH2A1 levels<br>0<br>0.0830<br>0.1740<br>macroH2A2 levels<br>1<br>0.0998<br>0.1215			macroH2A1 levels<br>(1-2) 0.001058<br>(1-3) 0.1724<br>(2-3) 0.0005828<br>macroH2A2 levels<br>(1-2) 0.09058<br>(1-3) 0.002778<br>(2-3) 0.004329
**S1D**	(1) Scramble<br>(2) mH2A1 KD<br>(3) mH2A2 KD		32/<br>43/<br>47/	1.1596483<br>0.6617645<br>1.1893396			0.4059139<br>0.5898538<br>0.1073395			(1-2) 4.093e-15<br>(2-3) 6.351e-10<br>(1-3) 0.6644
**S1F**	(1) Scramble<br>(2) mH2A1 KD		10/2<br>11/2	1<br>0.3583			0.1522<br>0.0501			(1-2) 5.67e-06<br>(1-3) 0.1083

<table>
<tr><td></td><td>(3) mH2A2 KD</td><td colspan="2">15/2</td><td colspan="2">0.9034</td><td colspan="2">0.1654</td><td colspan="2">(2-3) 4.487e-07</td></tr>
<tr><td>S2A</td><td>(1) Scramble
(2) mH2A1 KD
(3) mH2A2 KD

(4) WT
(5) mH2A1 KO
(6) mH2A2 KO</td><td colspan="2">86/2
161/2
126/2

35/2
49/2
51/2</td><td colspan="2">72
80
75

100
79.4
91.6</td><td colspan="2">16.9
8.3
20.2

0
3.0
3.9</td><td colspan="2">(1-2) 0.3005
(1-3) 0.3005
(2-3) 0.9012

(1-2) 0.02107
(1-3) 0.02107
(2-3) 0.3429</td></tr>
<tr><td>S2C</td><td>
(1) Scramble
(2) mH2A1 KD
(3) mH2A2 KD</td><td colspan="2">
141/4
129/4
167/4</td><td><u>Nuclear</u>
1.37
1.33
1.44</td><td><u>On Xi</u>
1.82
1.79
1.91</td><td><u>Nuclear</u>
0.29
0.28
0.35</td><td><u>On Xi</u>
0.64
0.60
0.60</td><td><u>Nuclear</u>
(1-2) 0.19
(1-3) 0.95
(2-3) 0.16</td><td><u>On Xi</u>
(1-2) 0.14
(1-3) 0.79
(2-3) 0.18</td></tr>
<tr><td>S2D</td><td>(1) Scramble
(2) mH2A1 KD
(3) mH2A2 KD</td><td colspan="2">429/4
351/4
451/4</td><td colspan="2">94.89
95.60
95.31</td><td colspan="2">1.2709
1.8476
2.4300</td><td colspan="2">(1-2) 0.6857
(1-3) 0.6857
(2-3) 0.9998</td></tr>
<tr><td>S2E</td><td><u>Xi DAPI sum int. values</u>
(1) Scramble
(2) mH2A1 KD
(3) mH2A2 KD
<u>Nuclear DAPI sum int. values</u>
(1) Scramble
(2) mH2A1 KD
(3) mH2A2 KD</td><td colspan="2">
65/2
59/2
63/2

65/2
59/2
63/2</td><td colspan="2">
44197.4375
44215.94828
44010.16129

856386.2683
866365.0385
859657.4528</td><td colspan="2">
6283.270364
8633.156152
7440.519095

137572.7625
125122.2124
133474.9179</td><td colspan="2">
(1-2) 0.5367
(1-3) 0.9242
(2-3) 0.7787

(1-2) 0.7954
(1-3) 0.6583
(2-3) 0.9361</td></tr>
<tr><td rowspan="2">S2F</td><td>
(1) Scramble
(2) mH2A1 KD
(3) mH2A2 KD</td><td>H3K9ac
80/2
70/2
62/2</td><td>H4K8ac
19/2
28/2
51/2</td><td><u>Nuclear</u>
H3K9ac
1.00
0.94
1.08
H4K8ac
1.00
0.92
0.96</td><td><u>On Xi</u>
H3K9ac
1.00
0.98
0.93
H4K8ac
1.00
0.86
0.92</td><td><u>Nuclear</u>
H3K9ac
0.55
0.09
1.09
H4K8ac
0.28
0.16
0.16</td><td><u>On Xi</u>
H3K9ac
0.38
0.19
0.21
H4K8ac
0.43
0.23
0.27</td><td><u>H3K9ac</u>
<u>Nuclear</u>
(1-2) 0.23
(1-3) 0.73
(2-3) 0.41
<u>H3K9ac</u>
<u>On Xi</u>
(1-2) 0.28
(1-3) 0.68
(2-3) 0.13</td><td><u>H4K8ac</u>
<u>Nuclear</u>
(1-2) 0.43
(1-3) 0.78
(2-3) 0.33
<u>H4K8ac</u>
<u>On Xi</u>
(1-2) 0.37
(1-3) 0.53
(2-3) 0.55</td></tr>
<tr><td>
(1) Scramble
(2) mH2A1 KD
(3) mH2A2 KD

(4) WT
(5) mH2A1 KO
(6) mH2A2 KO</td><td>H3K9ac
295/5
363/5
314/5

<u>Nuclear</u>
(4) 17/2
(5) 25/2
(6) 30/2
<u>On Xi</u>
(4) 15/2
(5) 12/2
(6) 13/2</td><td>H4K8a33
04/6
414/6
463/6

<u>Nuclear</u>
(4) 13/2
(5) 12/2
(6) 10/2
<u>On Xi</u>
(4) 17/2
(5) 12/2
(6) 18/2</td><td><u>Nuclear</u>
H3K9ac
1.00
1.00
1.03
H4K8ac
1.00
1.00
0.90

<u>Nuclear</u>
H3K9ac
1.00
1.00
0.94
H4K8ac
1.00
0.99
0.88</td><td><u>On Xi</u>
H3K9ac
1.0
1.1
1.03
H4K8ac
1.0
1.0
1.0

<u>On Xi</u>
H3K9ac
1.00
1.11
1.02
H4K8ac
1.00
0.99
0.91</td><td><u>Nuclear</u>
H3K9ac
0.03
0,03
0.03
H4K8ac
0.04
0.04
0.03

<u>Nuclear</u>
H3K9ac
0.09
0.08
0.11
H4K8ac
0.18
0.20
0.11</td><td><u>On Xi</u>
H3K9ac
0.07
0.04
0.03
H4K8ac
0.07
0.07
0.06

<u>On Xi</u>
H3K9ac
0.4
0.3
0.2
H4K8ac
0.39
0.50
0.29</td><td colspan="2">(1) | (2) | (3)
0.03 | 0,03 | 0.03
0.04 | 0.04 | 0.03
0.07 | 0.04 | 0.03

(4) | (5) | (6)
0.10 | 0.11 | 0.07
0.19 | 0.26 | 0.13
0.2 | 0.2 | 0.1</td></tr>
<tr><td>S5B</td><td><u>Diameter</u>
(1) Scramble
(2) Scramble w/
<u>Surface area</u>
(1) Scramble
(2) Scramble w/
<u>Flattening (z-axis)</u>
(1) Scramble
(2) Scramble w/</td><td colspan="2">
126/2
132/2

126/2
132/2

126/2
132/2</td><td colspan="2">
19.69808
22.40137

1366.215
2000.819

0.575602436
0.500147502</td><td colspan="2">
2.853629
2.513256

362.5866
427.1600

0.089216921
0.074458667</td><td colspan="2"><u>Diameter</u>
(1-2) 2.2e-16

<u>Surface area</u>
(1-2) 2.2e-16

<u>Flattening (z-axis)</u>
2.386e-11</td></tr>
<tr><td>S5D</td><td><u>Decondensation</u>
(1) Scramble</td><td colspan="2">
157/3</td><td colspan="2">
387.128</td><td colspan="2">
97.004</td><td colspan="2">
(1-2) 2.2e-16</td></tr>
</table>

	(2) Scramble w/	135/3	111.305	30.516	
**S5F**	Class 1<br>(1) Scramble<br>(2) Scramble w/<br>Class 2<br>(1) Scramble<br>(2) Scramble w/<br>Class 3<br>(1) Scramble<br>(2) Scramble w/<br>Class 4<br>(1) Scramble<br>(2) Scramble w/<br>Class 5<br>(1) Scramble<br>(2) Scramble w/<br>Class 6<br>(1) Scramble<br>(2) Scramble w/<br>Class 7<br>(1) Scramble<br>(2) Scramble w/	For all classes:<br>17/2<br>12/2	Class 1<br>0.300<br>0.463<br>Class 2<br>0.170<br>0.174<br>Class 3<br>0.220<br>0.130<br>Class 4<br>0.186<br>0.106<br>Class 5<br>0.084<br>0.069<br>Class 6<br>0.029<br>0.042<br>Class 7<br>0.011<br>0.016	Class 1<br>0.066<br>0.013<br>Class 2<br>0.019<br>0.014<br>Class 3<br>0.026<br>0.014<br>Class 4<br>0.026<br>0.008<br>Class 5<br>0.011<br>0.009<br>Class 6<br>0.006<br>0.006<br>Class 7<br>0.002<br>0.003	Class 1<br>(1-2) 3.85e-08<br><br>Class 3<br>(1-2) 3.85e-08<br><br>Class 4<br>(1-2) 3.85e-08
**S7A**	Data set name	Chromosome	Number of sites	Sites density (Mean value)	Sites density (Standard deviation value)
	DF (male) mH2A1	Autosomes<br>Chromosome X<br>Chromosome Y	155871<br>388<br>15	51.29<br>1.82<br>0.13	7.24<br>-<br>-
	MEF (female) mH2A1	Autosomes<br>Chromosome X<br>Chromosome Y	295945<br>17253<br>-	48.53<br>40.62<br>0.00	7.37<br>-<br>-
	MEF (male) mH2A1.1	Autosomes<br>Chromosome X<br>Chromosome Y	18217<br>311<br>14	50.93<br>12.37<br>1.04	7.18<br>-<br>-
	MEF (male) mH2A1.2	Autosomes<br>Chromosome X<br>Chromosome Y	16201<br>178<br>24	51.03<br>8.00<br>2.01	11.00<br>-<br>-
	DF (female) mH2A2	Autosomes<br>Chromosome X<br>Chromosome Y	150799<br>14771<br>-	45.96<br>64.70<br>0.00	7.59<br>-<br>-
	MEF (female) mH2A2	Autosomes<br>Chromosome X<br>Chromosome Y	256807<br>9462<br>-	50.58<br>26.72<br>0.00	6.62<br>-<br>-
	MEF (male) mH2A2	Autosomes<br>Chromosome X<br>Chromosome Y	43717<br>1085<br>-	49.30<br>17.56<br>0.33	7-73<br>-<br>-
**S8C**	(1) Scramble<br>(2) mH2A1 KD<br>(3) mH2A2 KD	2702/NA<br>2804/NA<br>15202/NA	0.930<br>0.774<br>0.894	0.033<br>0.159<br>0.066	2.2e-16 for all groups
**S13A**	(1) Scramble<br>(2) mH2A1 KD<br>(3) mH2A2 KD<br><br>t (h): 1, 2, 3, 4, 5, 6, 7, 8	24-28/<br>15-19/<br>15-20/	(1) / (2) / (3)<br>1 / 1.11 / 1.13<br>1.22 / 1.29 / 1.19<br>1.45 / 1.56 / 1.29<br>1.49 / 1.57 / 1.37<br>1.60 / 1.73 / 1.47<br>1.77 / 1.97 / 1.64<br>2.39 / 2.57 / 2.08<br>4.55 / 4.30 / 4.13	(1) / (2) / (3)<br>0.17 / 0.18 / 0.41<br>0.24 / 0.41 / 0.31<br>0.25 / 0.40 / 0.20<br>0.27 / 0.35 / 0.39<br>0.24 / 0.31 / 0.35<br>0.29 / 0.28 / 0.38<br>0.68 / 0.43 / 1.32<br>1.07 / 0.84 / 1.4	t (4 hours)<br>(1-2) 0.1617<br>(1-3) 0.1475<br>(2-3) 0.01231<br><br>t (8 hours)<br>(1-2) 0.000148<br>(1-3) 2.085e-05<br>(2-3) 0.5168

**S13 B-C**	Mcm2<br>(1) Scramble<br>(2) mH2A1 KD<br>(3) mH2A2 KD<br>Mcm2-phosphoS108<br>(1) Scramble<br>(2) mH2A1 KD<br>(3) mH2A2 KD		 7796/2<br>6121/2<br>7919/2<br><br>5870/2<br>6910/2<br>6466/2	 1.0023432<br>1.0012321<br>1.0123213			 2.73457345<br>2.41325435<br>2.84534534			Mcm2<br>(1-2) 0.1244<br>(1-3) 0.8102<br>(2-3) 0.07465<br>Mcm2-phosphoS108<br>(1-2) 0.6618<br>(1-3) 0.06689<br>(2-3) 0.1	
**S13D**	Cell cycle classification:<br>G1<br>Early S<br>Mid S<br>Late S		 145<br>75<br>61<br>44	 848184.5103<br>1127055.813<br>1378228.689<br>1756286.136			 237912.7482<br>163658.6411<br>403273.4059<br>393879.2405			 NA for all	
**S16A**	 (1) Scramble<br>(2) mH2A1 KD<br>(3) mH2A2 KD	t (h)<br>1<br>2<br>3<br>4<br>5<br>6<br>7<br>8	 42-49/4<br>39-49/4<br>41-44/4	 1<br>1.53<br>2.33<br>3.07<br>3.29<br>3.57<br>3.70<br>3.86	 0.84<br>1.49<br>2.32<br>3.05<br>3.28<br>3.60<br>3.73<br>3.90	 0.93<br>1.60<br>2.21<br>3.08<br>3.27<br>3.60<br>3.72<br>3.92	 0.35<br>0.62<br>0.23<br>0.54<br>0.56<br>0.58<br>0.72<br>0.91	 0.26<br>0.63<br>0.48<br>0.59<br>0.45<br>0.81<br>0.60<br>0.72	 0.32<br>0.60<br>0.19<br>0.38<br>0.47<br>0.42<br>0.56<br>0.54	t (4 hours)<br>(1-2) 0.7973<br>(1-3) 0.6181<br>(2-3) 0.3<br><br>t (8 hours)<br>(1-2) 0.767<br>(1-3) 0.946<br>(2-3) 0.6211	
**S16B**	 (1) Scramble<br>(2) mH2A1 KD<br>(3) mH2A2 KD	t (h)<br>1<br>2<br>3<br>4<br>5<br>6<br>7<br>8	 22-29/2<br>22-24/2<br>22-24/2	 1<br>1.54<br>2.79<br>2.97<br>3.52<br>3.79<br>3.91<br>4.03	 0.94<br>1.62<br>2.75<br>2.95<br>3.52<br>3.86<br>3.91<br>4.06	 0.89<br>1.55<br>2.79<br>2.96<br>3.53<br>3.79<br>3.89<br>4.05	 0.33<br>0.20<br>0.35<br>0.50<br>0.75<br>0.77<br>0.95<br>1.04	 0.33<br>0.34<br>0.36<br>0.31<br>0.41<br>0.82<br>0.76<br>0.59	 0.42<br>0.13<br>0.26<br>0.20<br>0.42<br>0.48<br>0.49<br>0.50	t (4 hours)<br>(1-2) 0.8151<br>(1-3) 0.9627<br>(2-3) 0.7984<br><br>t (8 hours)<br>(1-2) 0.7956<br>(1-3) 0.9706<br>(2-3) 0.9077	
**S16C**	 (1) Scramble<br>(2) mH2A1 KD<br>(3) mH2A2 KD	t (h)<br>1<br>2<br>3<br>4<br>5<br>6<br>7<br>8	 17-21/2<br>19-23/2<br>19-20/2	 1<br>1.16<br>2.56<br>4.11<br>4.30<br>4.69<br>5.03<br>5.28	 0.88<br>1.20<br>2.70<br>4.20<br>4.35<br>4.68<br>5.06<br>5.33	 0.93<br>1.19<br>2.67<br>4.16<br>4.34<br>4.72<br>5.10<br>5.26	 0.34<br>0.26<br>0.35<br>0.62<br>0.69<br>0.69<br>0.35<br>0.76	 0.26<br>0.37<br>0.82<br>0.94<br>0.69<br>1.06<br>0.78<br>0.93	 0.32<br>0.43<br>0.23<br>0.29<br>0.70<br>0.58<br>0.54<br>0.63	t (4 hours)<br>(1-2) 0.7381<br>(1-3) 0.718<br>(2-3) 0.8201<br><br>t (8 hours)<br>(1-2) 0.7994<br>(1-3) 0.9467<br>(2-3) 0.8831	
**S19A**	 (1) Scramble<br>(2) mH2A1 KD<br>(3) mH2A2 KD		 53/2<br>51/2<br>71/2	Nuclear<br>0.74<br>0.75<br>0.76	On Xi<br>0.37<br>0.40<br>0.40		Nuclear<br>0.05<br>0.04<br>0.04	On Xi<br>0.11<br>0.13<br>0.12		Nuclear<br>(1-2) 0.87<br>(1-3) 0.12<br>(2-3) 0.09	On Xi<br>(1-2) 0.25<br>(1-3) 0.07<br>(2-3) 0.61
**S19B**	 (1) Scramble<br>(2) mH2A1 KD<br>(3) mH2A2 KD		 74/2<br>75/2<br>81/2	Nuclear<br>0.79<br>0.82<br>0.79	On Xi<br>0.54<br>0.59<br>0.58		Nuclear<br>0.10<br>0.09<br>0.11	On Xi<br>0.20<br>0.19<br>0.19		Nuclear<br>(1-2) 0.045<br>(1-3) 0.83<br>(2-3) 0.06	On Xi<br>(1-2) 0.07<br>(1-3) 0.07<br>(2-3) 0.15
**S19C**	 (1) Scramble<br>(2) mH2A1 KD<br>(3) mH2A2 KD		 62/2<br>71/2<br>85/2	Nuclear<br>1.20<br>1.12<br>1.12	On Xi<br>1.49<br>1.29<br>1.34		Nuclear<br>0.21<br>0.16<br>0.20	On Xi<br>0.46<br>0.39<br>0.41		Nuclear<br>(1-2) 0.025<br>(1-3) 0.112<br>(2-3) 0.540	On Xi<br>(1-2)<br>0.018<br>(1-3)<br>0.115<br>(2-3)<br>0.426
***t-test instead of Wilcox test**											

NA: not applicable
n: number of cells of all replicates (if not stated otherwise), StDev: standard deviation, 95% CI: 95% confidence interval, p-value: calculated as stated in figure legends and material and methods section.
n.s., not significant, is given for p-values > or equal to 0.05; one star (*) is given for p-values < 0.05 and > or equal to 0.005; two stars (**) is given for values < 0.005 and > or equal to 0.0005; three stars (***) is given for values < 0.0005. The P-values from independent two-group comparisons are shown in the table. The numbers assigned to each group are in brackets. For example, in Figure 3C, 0.7387 is the p-value obtained after the comparison (1-3), 1 Scramble, and 3 mH2A2 KD.

Supplementary Table 9: Software and macros

Name	Version	Website	Company/Institution	Application
Volocity	6.3	-	PerkinElmer, USA	Acquiring live cell time lapses
ImageJ	1.53c	https://imagej.nih.gov/ij/	Wayne Rasband, National Institutes of Health, USA	Image processing and image analysis
RStudio	1.1.447-1.2.5033	https://rstudio.com/	RStudio	Statistical analysis and plotting
Harmony	3.5.1	https://www.perkinelmer.com/product/harmony-4-8-office-hh17000001	PerkinElmer, USA	High-content microscopy imaging and analysis
Benchling	Web-based	http://benchling.com	Benchling, CA, USA	Cloning and plasmids
Adobe Illustrator CS6	16	https://www.adobe.com/	Adobe, USA	Graphical sketch and figure arrangement
PyMOL	3.0	https://www.pymol.org/	Schrodinger	Generation of protein structure images
Python	3.12.4	https://www.python.org/	Python Software Foundation	Data analysis and plotting for AlphaFold modeling
AlphaFold-Multimer	2.3.2	-	-	Protein structure modeling

Supplementary references

1. Buschbeck,M., Uribesalgo,I., Wibowo,I., Rué,P., Martin,D., Gutierrez,A., Morey,L., Guigó,R., López-Schier,H. and Di Croce,L. (2009) The histone variant macroH2A is an epigenetic regulator of key developmental genes. *Nat. Struct. Mol. Biol.*, **16**, 1074–1079.
2. Mašata,M., Juda,P., Raška,O., Cardoso,M.C. and Raška,I. (2011) A fraction of MCM 2 proteins remain associated with replication foci during a major part of S phase. *Folia Biol (Praha)*, **57**, 3–11.
3. Pasque,V., Gillich,A., Garrett,N. and Gurdon,J.B. (2011) Histone variant macroH2A confers resistance to nuclear reprogramming. *EMBO J.*, **30**, 2373–2387.
4. Heinz,K.S., Casas-Delucchi,C.S., Török,T., Cmarko,D., Rapp,A., Raska,I. and Cardoso,M.C. (2018) Peripheral re-localization of constitutive heterochromatin advances its replication timing and impairs maintenance of silencing marks. *Nucleic Acids Res.*, **46**, 6112–6128.
5. Lindhout,B.I., Fransz,P., Tessadori,F., Meckel,T., Hooykaas,P.J.J. and van der Zaal,B.J. (2007) Live cell imaging of repetitive DNA sequences via GFP-tagged polydactyl zinc finger proteins. *Nucleic Acids Res.*, **35**, e107.
6. Sporbert,A., Gahl,A., Ankerhold,R., Leonhardt,H. and Cardoso,M.C. (2002) DNA polymerase clamp shows little turnover at established replication sites but sequential de novo assembly at adjacent origin clusters. *Mol. Cell*, **10**, 1355–1365.
7. Skene,P.J., Illingworth,R.S., Webb,S., Kerr,A.R.W., James,K.D., Turner,D.J., Andrews,R. and Bird,A.P. (2010) Neuronal MeCP2 is expressed at near histone-octamer levels and globally alters the chromatin state. *Mol. Cell*, **37**, 457–468.
8. Kapoor,A., Goldberg,M.S., Cumberland,L.K., Ratnakumar,K., Segura,M.F., Emanuel,P.O., Menendez,S., Vardabasso,C., Leroy,G., Vidal,C.I., *et al.* (2010) The histone variant macroH2A suppresses melanoma progression through regulation of CDK8. *Nature*, **468**, 1105–1109.
9. Telenius,H., Carter,N.P., Bebb,C.E., Nordenskjöld,M., Ponder,B.A. and Tunnacliffe,A. (1992) Degenerate oligonucleotide-primed PCR: general amplification of target DNA by a single degenerate primer. *Genomics*, **13**, 718–725.
10. Yaffe,D. and Saxel,O. (1977) Serial passaging and differentiation of myogenic cells isolated from dystrophic mouse muscle. *Nature*, **270**, 725–727.
11. Gaspar-Maia,A., Qadeer,Z.A., Hasson,D., Ratnakumar,K., Leu,N.A., Leroy,G., Liu,S., Costanzi,C., Valle-Garcia,D., Schaniel,C., *et al.* (2013) MacroH2A histone variants act as a barrier upon reprogramming towards pluripotency. *Nat. Commun.*, **4**, 1565.
12. Pehrson,J.R., Changolkar,L.N., Costanzi,C. and Leu,N.A. (2014) Mice without macroH2A histone variants. *Mol. Cell. Biol.*, **34**, 4523–4533.
13. Harrison,T., Graham,F. and Williams,J. (1977) Host-range mutants of adenovirus type 5 defective for growth in HeLa cells. *Virology*, **77**, 319–329.
14. Morita,S., Kojima,T. and Kitamura,T. (2000) Plat-E: an efficient and stable system for transient packaging of retroviruses. *Gene Ther.*, **7**, 1063–1066.
15. Peters,A.H., O'Carroll,D., Scherthan,H., Mechtler,K., Sauer,S., Schöfer,C., Weipoltshammer,K., Pagani,M., Lachner,M., Kohlmaier,A., *et al.* (2001) Loss of the Suv39h histone methyltransferases impairs mammalian heterochromatin and genome stability. *Cell*, **107**, 323–337.
16. Waseem,N.H. and Lane,D.P. (1990) Monoclonal antibody analysis of the proliferating cell nuclear antigen (PCNA). Structural conservation and the detection of a nucleolar form. *J. Cell Sci.*, **96 (Pt 1)**, 121–129.
17. Mulholland,C.B., Smets,M., Schmidtmann,E., Leidescher,S., Markaki,Y., Hofweber,M., Qin,W., Manzo,M., Kremmer,E., Thanisch,K., *et al.* (2015) A modular open platform for systematic functional studies under physiological conditions. *Nucleic Acids Res.*, **43**, e112.
18. Rothbauer,U., Zolghadr,K., Muyldermans,S., Schepers,A., Cardoso,M.C. and Leonhardt,H. (2008) A versatile nanotrap for biochemical and functional studies with fluorescent fusion proteins. *Mol. Cell. Proteomics*, **7**, 282–289.
19. Weber,P., Rausch,C., Scholl,A. and Cardoso,M.C. (2018) Repli-FISH (Fluorescence in Situ Hybridization): Application of 3D-(Immuno)-FISH for the Study of DNA Replication Timing of Genetic Repeat Elements. *OBM Genet.*, **3**.
